# Rapid pathogen identification using a novel microarray-based assay with purulent meningitis in cerebrospinal fluid

**DOI:** 10.1038/s41598-018-34051-0

**Published:** 2018-10-29

**Authors:** Yuting Hou, Xu Zhang, Xiaolin Hou, Ruofen Wu, Yanbai Wang, Xuexian He, Libin Wang, Zhenhai Wang

**Affiliations:** 1 Department of Cerebrospinal Fluid Laboratory, The General Hospital of Ningxia Medical University, Yinchuan, Ningxia, 750004 China; 2 Department of Beijing National Biochip Research Center sub-center in Ningxia, General Hospital of Ningxia Medical University, Yinchuan, Ningxia, 750004 China; 3 Ningxia Key Laboratory of Cerebrocranial Diseases, Ningxia Medical University, Yinchuan, Ningxia, 750004 China; 4 Neurology Center, The General Hospital of Ningxia Medical University, Yinchuan, Ningxia, 750004 China

## Abstract

In order to improve the diagnosis of pathogenic bacteria in cerebrospinal fluid (CSF) with purulent meningitis, we developed a DNA microarray technique for simultaneous detection and identification of seven target bacterium. DNA were extracted from 24 CSF samples with purulent meningitis (or suspected purulent meningitis). The specific genes of each pathogen were chosen as the amplification target, performed the polymerase chain reaction (PCR), labeled with a fluorescence dye, and hybridized to the oligonucleotide probes on the microarray. There is no significant cross-hybridization fluorescent signal occurred in untargeted bacteria. There were 87.5% (21/24) positive results in DNA microarray compared with the 58.3% (14/24) of the CSF culture test. Of which 58.3% (14/24) of the patients with culture-confirmed purulent meningitis, 37.5% (9/24) patients who were not confirmed by culture test but were demonstrated by the clinical diagnosis and DNA microarray. Multiple bacterial infections were detected in 5 cases by the microarray. In addition, the number of gene copies was carried out to determine the sensitivity of this technique, which was shown to be 3.5 × 10^1^ copies/μL. The results revealed that the microarray technique which target pathogens of the CSF specimen is better specificity, accuracy, and sensitivity than traditional culture method. The microarray method is an effective tool for rapidly detecting more target pathogens and identifying the subtypes of strains which can eliminate the impact of the different individuals with purulent meningitis for prompt diagnosis and treatment.

## Introduction

Meningitis, or inflammation of the meninges, is usually acute but can also be subacute and most frequently presents with headache, fever, and neck stiffness^1,2^. It was estimated that meningitis lead to 420,000 deaths in 2010 and killed 303,000 people in 2013 globally^3^. Meningitis can be pyogenic, and be called purulent meningitis (PM) or bacterial meningitis, is an emergency disease associated with high morbidity and mortality rates^4,5^. The PM can be caused by the different pathogenic bacteria^6^, included *S. pneumoniae*, *B Streptococcus*, *Listeria monocytogenes*, *Escherichia coli* and other bacteria^7–11^.

The routine laboratory diagnosis of PM depends on the cerebrospinal fluid (CSF) culture along with morphologic and chemical analysis of CSF^12,13^. Central nervous system (CNS) bacteria infections can cause elevated white blood cell (WBC) counts with 90% neutrophils^14^, elevated CSF protein concentration, lowered CSF glucose levels, half of the patients with CSF can be found bacterial pathogens after centrifugal smear by Gram stain^15^. However, these values, most notably that CSF WBC counts, can be normal even in the case of PM^16^. Hence, CSF culture remains critical to establishing the diagnosis of PM^17^. The most important troubling problem is that CSF culture usually need 5~7 days to obtain the results for in critically ill patient requiring immediate therapying. Moreover, the drug resistant bacteria raised an increasing serious public-health concern around the world^18^. Therefore, the rapid and accurate method for bacteria identification that is involved in an infectious disease should be focused by researcher. The molecular methods, especially the microarray technology present a new opportunity for fast and reliable diagnosis to detect the bacteria^19^.

In this study, a total of 5 pairs of primers and 156 oligonucleotide probes, according to the available sequence database, were used to develop a microarray which have a power ability to simultaneous determine targets bacteria, which are examples of highly life-threatening with central nervous system infection. The bacterial pathogens panel of the assay covered the following species: *Escherichia coli (E.coli), Neisseria meningitides (Nm)*, *Streptococcus pneumoniae (Sp), Haemophilus influenza (Hi), Staphylococcus aureus (Sa), Staphylococcus epidermidis (Se)*, and *Coagulase negative staphylococcus (CN-S)*. This study described the validation of microarray technique that included five genes from seven pathogens species for identifying bacterial pathogens by an isolated DNA sample from the CSF specimens with PM.

## Materials and Methods

### Inclusion and exclusion criteria of patient

Twenty-four cases of patients were diagnosed with purulent meningitis (or suspected purulent meningitis) and 20 cases of negative control (NC, Table S1) were diagnosed with non-bacterial infectious diseases were collected in General Hospital of Ningxia Medical University (Yinchuan, China) between May 2015 and May 2018. The suspected purulent meningitis consisted of 13 males and 11 females, ranging between 1–71 years old (average 28.8 ± 15.6 years). Diagnosis of purulent meningitis was made with reference to clinical and CSF examination and was verified by CSF cultur-based test in all patients. The diagnostic criteria for inclusion as suspected purulent meningitis met any of the follows: (1) Fever (>38.5 °C rectal or >38.0 °C axillary), intracranial hypertension symptoms (headache, vomiting, high tension in anterior fontanelle of infants, disturbance of consciousness), meningeal irritation signs (neck resistance, positive Kernig’s sign or Brudzinski’s sign and opisthotonos), and with macroscopic aspect of CSF turbid, cloudy or purulent; or with CSF pleocytosis mainly neutrophilic and white blood cells (WBCs) ≥100 cells/mm^3^. (2) Fever, intracranial hypertension symptom, meningeal irritation signs and the CSF WBCs counts slightly to moderately elevated, positive response to anti-bacterial treatment. (3) During the application of antibiotics, it appears fever, atypical cranial hypertension symptoms and slightly CSF WBCs elevated, and one of the following situations: (a) The IgM of the antispecific pathogen in CSF reaches the diagnostic criteria, or the IgG is 4 times higher, or the CSF smear finds the bacteria; (b) Have the history of invasive craniocerebral operation, or head trauma or lumbar puncture; (c) There is focal infection near the meninges, or have the CSF leakage, (d) The blood culture positive of newborn. Confirmed case (of purulent meningitis): Isolation or identification of the causal pathogen from the cerebrospinal fluid (CSF) of a suspected case by culture. The criteria for inclusion as negative control as follows: (1) Diagnosed with one of the disease including: Neuromyelitis optica, Anti-NMDA receptor encephalitis, Viral Meningitis, Epidemic encephalitis B, Cerebral infarction, Guillain-barre syndrome, Multiple sclerosis, Acute lymphoblastic leukemia and Central nervous system leukemia. (2) Negative cerebrospinal fluid (CSF) culture.

### The standard bacterial strains

The reference strains purchased from the American type culture collection (ATCC) and used in the study for evaluating the specificity of the oligonucleotide probes, included *Escherichia coli* (ATCC 25922), *Neisseria meningitides* (ATCC 13077), *Streptococcus pneumoniae* (ATCC 49619)*, Haemophilus influenza* (ATCC 49247)*, Staphylococcus aureus* (ATCC 25923), *Staphylococcus epidermidis* (ATCC 12228), *Proteus mirabilis* (ATCC 7002), *Klebsiella pneumoniae* (ATCC 13883), *Pseudomonas aeruginosa* (ATCC 9027) and *Coagulase-negative staphylococcus* (CNS) *S. haemolyticus* (ATCC 29970), *S. hominis* (ATCC 27844).

### Cerebrospinal fluid (CSF) preparation

A total of 44 clinical CSF samples were obtained by a lumbar puncture and were received fresh. Firstly the samples were performed CSF cytology examination, we put 0.5 mL cerebrospinal fluid into TPX Sample Chamber (Thermo Scientific, Inc., US). The cells would be centrifuged at 1,000 rpm for 5 minutes by Cytospin 4 Cytocentrifuge (Thermo Scientific, Inc., US) onto the slide. Then, the slide was airdried and stained with Wright Giemsa Strain (BA4017, BASO, Inc., ZhuHai, China), which exhibited the obviously increasing of the white blood cell counts ranged from 180 ~ 14,000 × 10^6^/liter and neutrophils accounted for more than 50%. The residual CSF samples were centrifuged immediately at 12,000 rpm for 10 minutes at room temperature, the supernatant was discarded and the pellet was batched and stored at −80 °C until use. The Ethics Commission (General Hospital of Ningxia Medical University, Yinchuan, China) approved all the clinical cases and all patients involved in the study provided informed consent.

### CSF culture testing

A collection of CSF culture testing^20^ were determined by the Vitek-2 automated system (bioMerieux, Marcy l’Etoile, France), which were examined by the clinical laboratory in General Hospital of Ningxia Medical University according to the National Clinical Test Regulation of Operation. Collection and transportation of specimens were strictly executed the sterile operating procedures.

### Design the probe and PCR primer

We obtained all the standard nomenclature of the target microorganism. Using target genes 16*S rRNA* (Highly conserved sequence in microbial evolution), *gyrB* (Better conserved sequence in distinguishing the subtypes of strains), *gsp* (the specific gene in *Staphylococcus aureus)* and *nuc* (the specific gene in *Staphylococcus aureus)* for probes designing to germplasm identification^21^. And then obtained the sequences of the target genes from the Ribosomal Database Project (RDP), Silva database, and GenBank database (Table 1). Next, we used the Muscle software for multiple sequence alignment and obtained the consensus sequences. Subsequently, those sequences were used to design the oligonucleotide probes which generally according to: (a) 40% <GC content <60%; (b) the maximum and minimum of Tm value difference control within +/−3 °C, (c) the probe will not form the hairpin and the reverse complementary sequence cannot be longer than 5 mer; (d) can not have more than 4 consecutive single-base repetition; (e) will not form dimers. The results of these probe sequences were confirmed by BLAST analysis to verify if the probe sequence would pairing with the other sequences in general DNA sequences. Last, we screening the probe sequences and obtained the specific probe. All probe sequences was syntheticed by Sangon Biotech (Shanghai) Co., Ltd.

**Table 1:** Target strains information and sequences gather information.

NO.	Target strains Standard Nomenclature	Lineage (Full)	Taxonomy ID	Sequence gather information			
				*16S rRNA*	*gyrB*	*gsp*	*nuc*
1	*Haemophilus influenzae*	cellular organisms; Bacteria; Proteobacteria; Gammaproteobacteria; Pasteurellales; Pasteurellaceae; Haemophilus	727	675	29		
2	*Escherichia coli*	cellular organisms; Bacteria; Proteobacteria; Gammaproteobacteria; Enterobacteriales; Enterobacteriaceae; Escherichia	562	3342	855		
3	*Staphylococcus aureus*	cellular organisms; Bacteria; Firmicutes; Bacilli; Bacillales; Staphylococcaceae; Staphylococcus	1280	623	169	49	78
4	*Neisseria meningitidis*	cellular organisms; Bacteria; Proteobacteria; Betaproteobacteria; Neisseriales; Neisseriaceae; Neisseria	487	1349	30		
5	*Staphylococcus epidermidis*	cellular organisms; Bacteria; Firmicutes; Bacilli; Bacillales; Staphylococcaceae; Staphylococcus	1282	331	32		
6	*Coagulase Negative Staphylococci*	cellular organisms; Bacteria; Firmicutes; Bacilli; Bacillales; Staphylococcaceae; Staphylococcus	1279	539	14		
7	*Streptococcus pneumoniae*	cellular organisms; Bacteria; Firmicutes; Bacilli; Lactobacillales; Streptococcaceae; Streptococcus	1313	1166	235		

### Oligonucleotide chip construction

**Figure 1:**
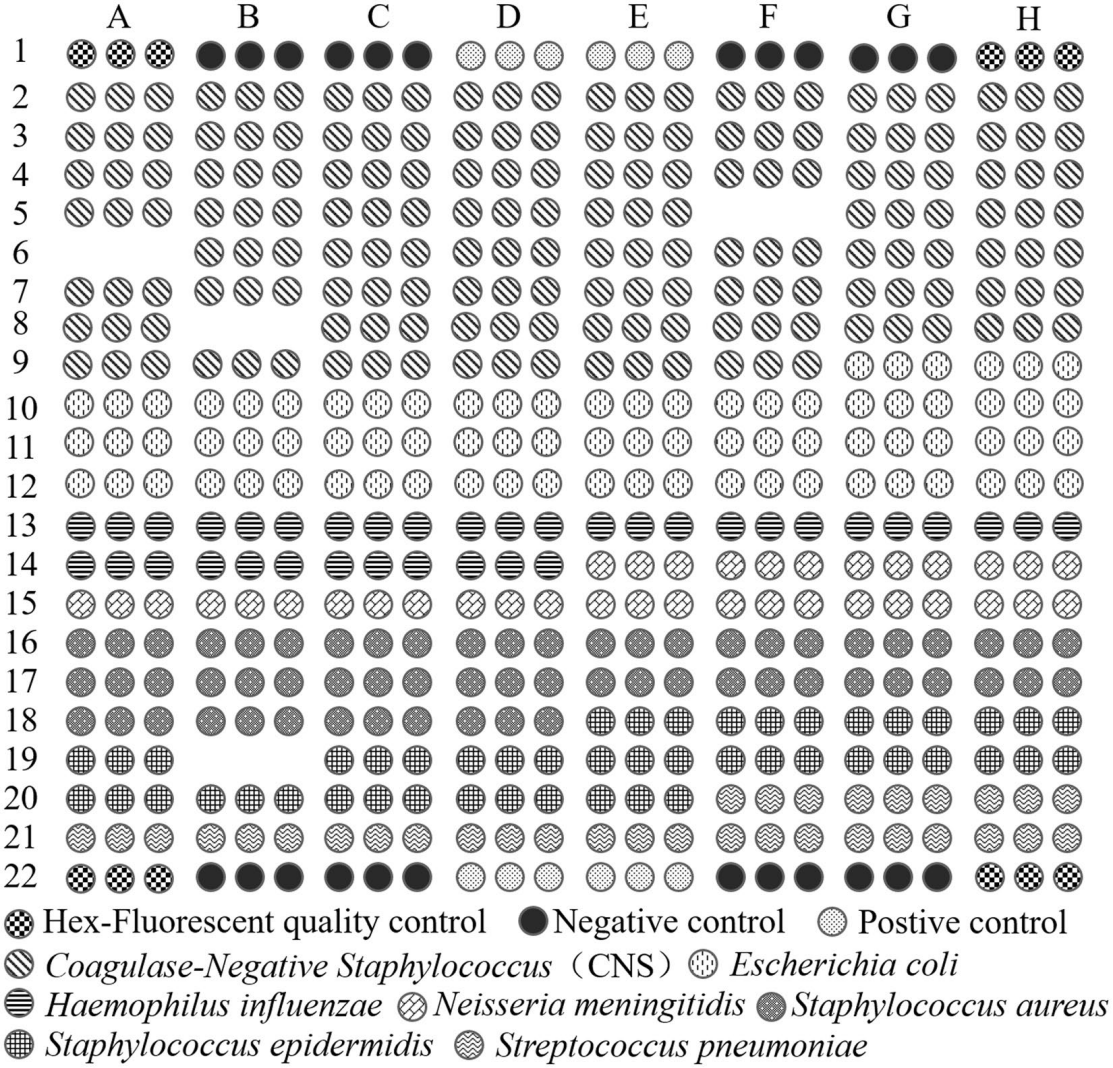
Oligonucleotide probes for array positioning.

Oligonucleotide probes were diluted in spotting buffer (440010-5, Beijing CapitalBio, Inc., China) to a concentration of 10 μmol/L and printed onto Optical aldehyde substrate using SmartArrayer-136 microarrayer system (Beijing CapitalBio, Inc., China)^22^. In total, 156 species-specific probes and 3 quality control probes were covalently immobilized on the slides via an amino group at their 5′ ends. Of which 59 *CNS*-specific, 26 *E.coli*-specific, 12 *H.influenza-*specific, 12 *N.meningitides-*specific, 20 *S.aureus-*specific, 16 *S.epidermidis-*specific and 11 *S.pneumoniae-*specific; Quality control probes including Hex-Fluorescent quality control to checkout the chip spot step (there was no signal if not spot well), Postive control to check out the sample hybridization step (there was no signal if no DNA hybridizing with the probes) and Negative control to ensure the microarray was not out of date and pollution (Fig. 1). All oligonucleotide probes were spotted as triplicate on the array. Every spot on the microarray with 150 µm diameter and 250 space between two spots. The microarray fixed method including: (a) put the chip in wet and lightproof box and place at 37 °C for 16 h. (b) Wash the chip with 0.2% SDS and purify water in turn. (c) put the chip in sodium borohydride solution for 5 min and wash it using purified water twice. Last, put the chip in the box and store at 4 °C until use.

### Extraction of total DNA from CSF and amplification of target genes

Total DNA was extracted from CSF using QIAamp DNA mini Kit (51304; QIAGEN, Inc., German). Quantitative detection of DNA was performed with a NanoDrop ND-2000 spectrophotometer (Thermo Fisher Scientific, Inc., Wilmington, DE, USA). PCR method were used to amplify target genes (16*S rRNA*, *gyrB*, *gsp* and *nuc*). The reaction system (total, 20 µL) of every primer (Table 2) containing: 1 µL DNA (100 ng), 0.5 µL sense primer, 0.5 µL reverse primer, 8 µL ddH_2_O and 10 µL EX taq version 2.0 Mix (CatRR003, TAKARA Inc., Japanese). Reaction conditions were as follows: one cycle of 94 °C for 5 min, followed by 35 cycles of 94 °C for 5 min, TM for 30 sec and 72 °C for 90 sec; and final extension at 72 °C for 10 min. Following 1.2% agarose gel electrophoresis (100 V, 23 min, 1× TAE). The PCR production was stored at −80 °C until use.

**Table 2:** Target genes’ primer sequences and size of products (bp).

Genes	Primer names	Primer sequences (5′-3′)	Product (bp)	Target strains
*16S rRNA*	16s-F27	AGA GTT TGA TCC TGG CTC AG	1466	*S.pneumoniae, S.aureu, S.epidermidis, E.coli N.meningitides, H.influenza*
	16s-R1492	GGT TAC CTT GTT ACG ACT T		
*gyrB*	Spne-gyrB-64F	GAG GGC TTA GAG GCT GTT CG	1402	*S.pneumoniae*
	Spne-gyrB-1447R	CGC CAA ATC CTG TTC CCA T		
*gyrB*	rmyy-gyrB-2f	AAA AGA CCR GGT ATG TAT ATW GG	1200	*S.aureus, S.epidermidis, E.coli*
	rmyy-gyrB-2r	CCG GCA GAG TCM CCY TCK AC		
*gsp*	gsp-F	GGT ACT ACT AAA GAT TAT CAA GAC GGC T	147	*S.aureus*
	gsp-R	TTC TTC ACG ACT AAA TAA ACG CTC A		
*nuc*	nuc-F	GAA AGG GCA ATA CGC AAA GA	481	*S.aureus*
	nuc-R	AGC CAA GCC TTG ACG AAC TAA AGC		

### Preparation of labeled genes

Mixture the 5 PCR products equal concentration proportional and underwent fragmentation in a reaction system (total, 25 µL) containing: 5 µL PCR products (1,000 ng), 3 µL fluorescence labeling 9 N radom primer (Sangon Biotech, Int., Shanghai, China), 11 µL ddH_2_O, denaturation in 95 °C for 3 min and ice for 3 min; Then add 2.5 µL klenow buffer (5X), 1 µL klenow enzyme, 2.5 µL DNTP (2.5 mM). Reaction conditions were as follows: 37 °C for 1.5 h and 70 °C for 10 min using DNA Polymerase I Large Klenow Fragment (M0210L; New England Biolabs Inc., Ipswich, England).

### Hybridization, washing and scanning of the microarrays

The molecular hybridization instrument used in the present study was a CapitalBio Genchip Scan System, which included a BioMixer II GeneChip hybridization, SlibeWasherTM8 and GeneArray Luxscanner10K-A (CapitalBio, Inc., Beijing, China) with a purulent meningitis pathogenic bacterium detected array product at Beijing National Biochip Research Center sub-center in Ningxia^23,24^. Total of 15 µL fragmented gene products and 5 µL hybridization solution (CapitalBio, Inc., Beijing, China) were mixed and added to the genechip, followed by hybridization for 3 h at 55 °C. Then washing the genechip with solution 1 (2× SSC, 0.2% SDS) twice and solution 2 (0.2% SSC) three times. Scanning and imaging of the microarrays following washing with power 650.

### Data analysis

Obtained the image in the CapitalBio Luxscanner10K-A using software Luxscan 3.0. A spot show green fluorescence signal means the probe tested positive. At least one probe shows the signal means there was a target microorganism in the sample despite the plural probes detect the same microorganism (plural probes were using to distinguish the differert subtypes of the microorganism). Three repetitions must showed the same result or decided it’s a negative probe. All the probes for a specific bacterial target were required to be positive for that target to be classified as positively identified. Read the signal result of microarrays compare with the CSF culture testing.

## Results

### Evaluation of the specificity and sensitivity of the probes

**Figure 2:**
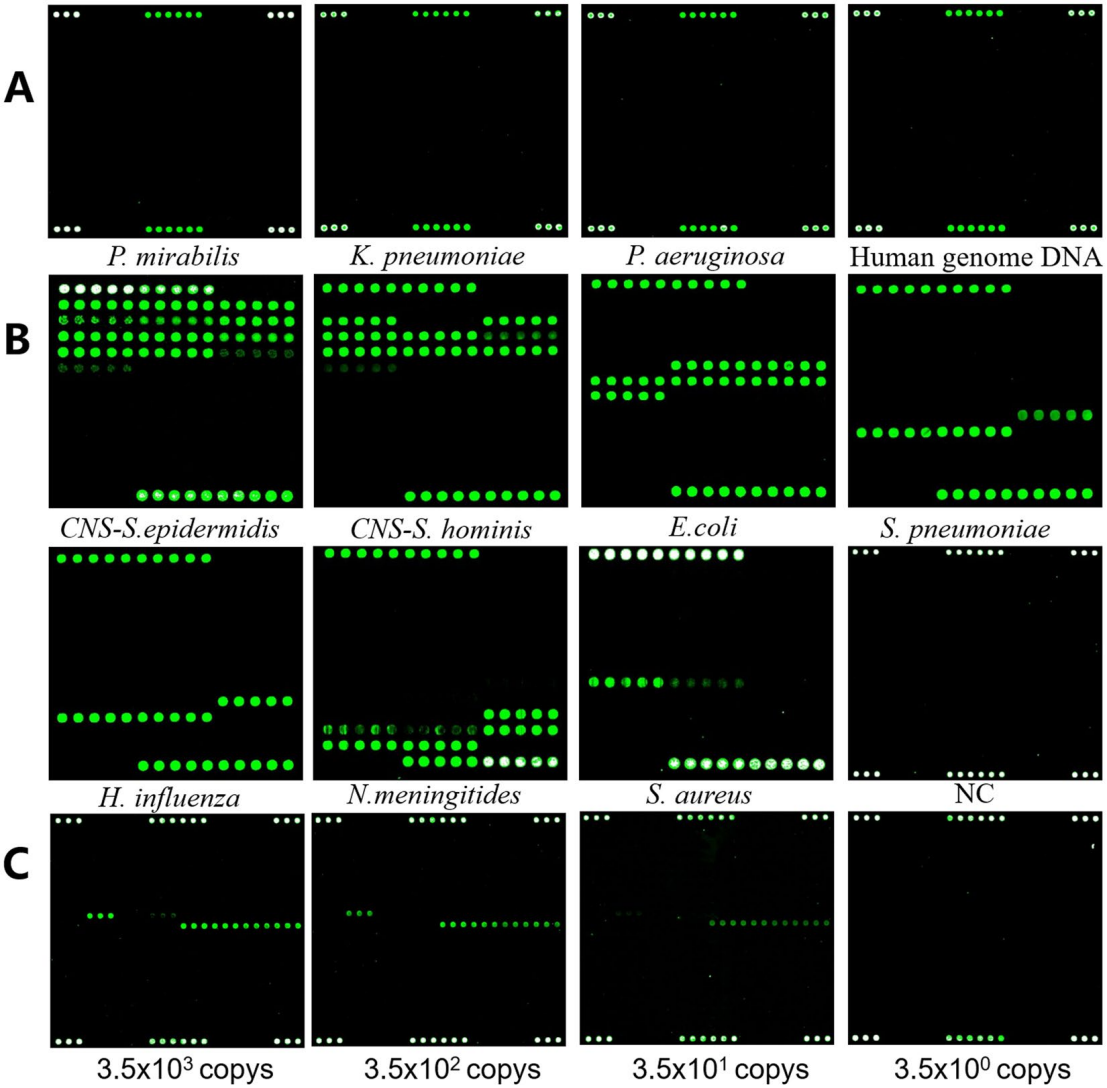
The specificity and sensitivity of the pathogen probes. (**A**) Microarray hybridized with the probe untarget bacteria and human genome DNA. (**B**) Microarray hybridized with the probe target bacteria. NC means microarray hybridized with ddH_2_O and positive control sequence (show the signal of PC probes). (**C**) Microarray hybridized with the *Escherichia coli* (ATCC 25922) which diluted for concentration gradient (the original DNA samples were extracted from CSF).

To evaluate the wet-lab specificity of the probes and avert any possible cross-hybridization that might lead to false positive. The standard strains *Staphylococcus epidermidis*, *Staphylococcus hominis*, *Streptococcus pneumoniae*, *Haemophilus influenza*, *Neisseria meningitides*, *Staphylococcus aureus* and untargeted bacteria such as *Proteus mirabilis*, *Klebsiella pneumoniae*, *Pseudomonas aeruginosa* and *Enterobacter cloacae* were subjected to PCR amplification and subsequent hybridization on the microarray to detect the specificity of the probes, In addition, we also detected the human genome. The results suggested that the fluorescent signal of standard strains were conformed with the location of the oligonucleotide probe (Fig. 2B). And there were no significant cross-hybridization fluorescent signal occurred in untargeted bacteria, even in human genome (Fig. 2A). The assay results showed high specificity of oligonucleotide probes in this microarray. In order to measure the sensitivity of the microarray, we calculated the copys of PCR products in 1 µL *E.coli* 16*S* DNA according to nucleic acid concentration and diluted the samples with copy numbers gradiently. The results showed positive correlation between copy numbers concentration and the power of fluorescent signal (Fig. 2C).

### The amplification of PCR

The primer locations were chosen to be relatively specific for bacterial genes. The specificity of the universal primers were assessed with DNA extracted from 6 standard bacterial strains and 2 specimens which was chosen randomly from all specimens. Every DNA sample (standard strains and specimens) prepared from the standard bacterial strains and CSF from patients were used for PCR amplification and following 1.2% agarose gel electrophoresis, the 5 pairs of specific primers for target strains yielded 400~1500 bp fragments (Table 2). No positive bands were seen in the negative controls.

**Figure 3:**
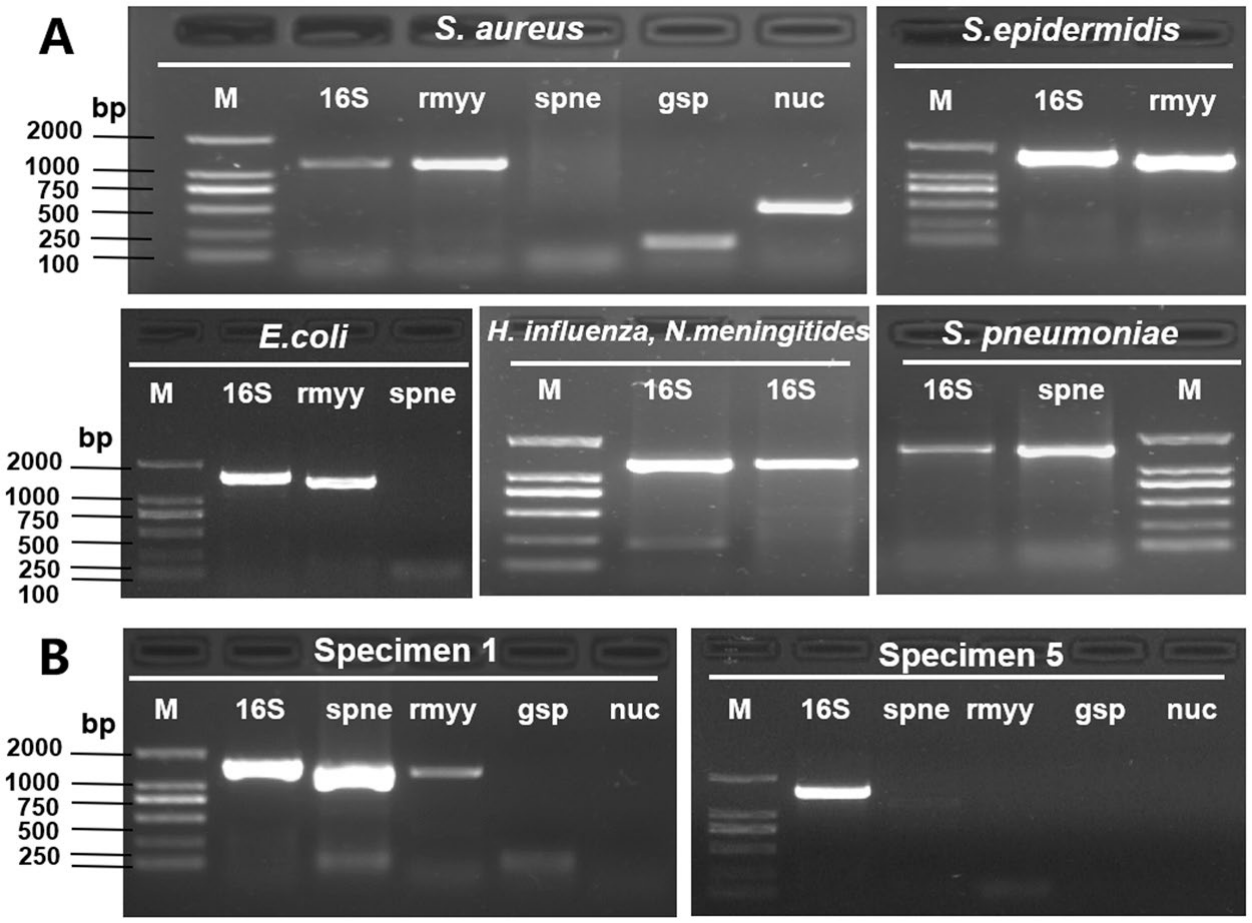
The gel images of PCR products using specific primers. (**A**) PCR products with 6 standard strains using specific primers. (**B**) PCR products with 2 specimens which was chosen randomly from all specimens.

The results showed specific primers were effective. Standard strains and specimens can be visible to the obvious bands. The DNA products can be used to hybridize the detective microarray (Fig. 3).

### The results of culture-based test

A total of the 24 suspected purulent meningitis cases were performed the culture-based test. Among these samples, 9 (37.5%) were negative, 6 (25%) were positive for *S.pneumoniae*, 2 (8.3%) were positive for both *S.aureus* and *E.coli*, one (4.2%) were positive for both *H.influenza and S.epidermidis*. In addition, *Citrobacter freundii* and *Listeria monocytogenes* were detected in one case respectively. The turnaround time of culture was 3~7 days (Table 3).

**Table 3:** Comparison between microarry, culture-based testing results.

Sample ID	Age/sex (years)	Symptom	The chemical analysis of CSF^a^			CSF cytology examination					The results of Blood culture testing	The results of CSF culture testing	The results of CSF microarry analysis	Diagnosis
			Pro (g/L)	Glu (mmol/L)	Cl (mmol/L)	WBC (/mm^3^)	L (%)	M (%)	N (%)	Bacteria^i^				
Specimen 1	41/F	Fever	4.48	2.0	112	967	14	17	69	NO	Negative	Negative	Negative	suspected case
Specimen 2	0.5/M	Fever, vomit	3.51	1.1	117	1,750	17	13	70	NO	Negative	Negative	*S.aureus*	suspected case
Specimen 3	43/M	Fever	3.63	3.3	120	1,760	8	22	70	NO	ND	Negative	*S.aureus*	suspected case
Specimen 4	13/F	Fever	2.93	1.3	120	2,480	5	12	83	NO	Negative	*S.aureus*	*S.aureus*	Purulent Meningitis
Specimen 5	44/F	Unconsciousness Fever	4.95	1.0	110	182	11	35	54	YES	*E.coli*	*E.coli*	*E.coli*	Purulent Meningitis
Specimen 6	38/F	Fever	2.46	1.9	116	825	7	15	78	NO	Negative	Negative	*S.aureus*	suspected case
Specimen 7	18/M	Headache, Fever Nausea, vomit	5.13	1.1	110	8,840	13	12	75	NO	Negative	*S.pneumoniae*	*S.pneumoniae, S.aureus*	Purulent Meningitis
Specimen 8	4/F	Fever Headache,vomit	0.57	1.5	112	2,250	8	8	84	NO	Negative	*Listeria monocytogenes*	Negative	Purulent Meningitis
Specimen 9	8/M	Fever	2.76	1,4	117	896	9	12	79	NO	Negative	Negative	CN-S	suspected case
Specimen 10	60/M	Unconsciousness	0.25	4.0	125	7	—	—	—	YES	ND	*Citrobacter freundii*	Negative	Purulent Meningitis
Specimen 11	0.58/M	Fever	8.82	1.0	101	280	5	7	88	YES	*S.pneumoniae*	*S.pneumoniae*	*S.pneumoniae, S.aureus*	Purulent Meningitis
Specimen 12	4/F	Fever Headache,	ND	ND	ND	4,250	2	3	97	YES	ND	ND	*S.pneumoniae, S.aureus*	Purulent Meningitis
Specimen 13	1/M	Fever, vomit	7.56	2.6	120	4,960	11	7	82	YES	Negative	*S.pneumoniae*	*S.pneumoniae, E.coli, S.aureus N.meningitides, CN-S*	Purulent Meningitis
Specimen 14	39/F	Fever Headache,vomit	4.68	1.1	88	915	12	11	77	NO	Negative	Negative	*S.aureus*	suspected case
Specimen 15	51/M	Encephalorrhagia Unconsciousness	2.09	3.0	117	2,280	11	14	75	NO	ND	*S.aureus*	*S.aureus*	Purulent Meningitis
Specimen 16	1/M	Fever, vomit	1.89	3.1	117	11,480	9	7	84	NO	Negative	*S.pneumoniae*	*S.pneumoniae, S.aureus*	Purulent Meningitis
Specimen 17	12/F	Postoperative Infection	4.95	1.2	113	190	15	37	72	NO	Negative	Negative	*E.coli*	suspected case
Specimen 18	9/F	Fever	3.59	1.1	110	400	26	15	59	YES	Negative	*S.pneumoniae*	*S.pneumoniae*	Purulent Meningitis
Specimen 19	40/F	Fever,Nausea, vomit, Unconsciousness	10.35	1.1	112	67	1	2	97	YES	*S.pneumoniae*	*S.pneumoniae*	*S.pneumoniae*	Purulent Meningitis,
Specimen 20	0.4/M	Fever,Nausea, vomit,	1.03	1.1	113	687	7	20	72	YES	*E.coli*	*E.coli*	*E.coli*	Purulent Meningitis
Specimen 21	12/F	Fever	4.97	1.6	115	834	12	18	86	NO	Negative	Negative	*CN-S*	suspected case
Specimen 22	0.25/M	Fever, Tic, Unconsciousness	3.61	1.0	111	607	13	32	54	YES	Negative	*H.influenza*	*H.influenza*	Purulent Meningitis
Specimen 23	9/M	Fever	4.33	1.0	117	709	18	20	49	NO	Negative	*S.epidermidis*	*S.epidermidis*	Purulent Meningitis
Specimen 24	14/M	Fever Headache,nausea	7.04	1.1	116	13,280	4	3	93	NO	ND	Negative	*N.meningitides*	Purulent Meningitis

Notes: CSF-cerebrospinal fluid; Pro-protein contents of cerebrospinal fluid; Glu-glucose contents of cerebrospinal fluid; Cl-chloride contents of cerebrospinal fluid; WBC-white blood cells ofcerebrospinal fluid; L-lymphocyte count of cerebrospinal fluid; M-monocyte count of cerebrospinal fluid; N-neutrophils count of cerebrospinal fluid; Bacteria^i^ -Cerebrospinal fluid cytology examination.

### Microarray analysis of clinical CSF

**Figure 4:**
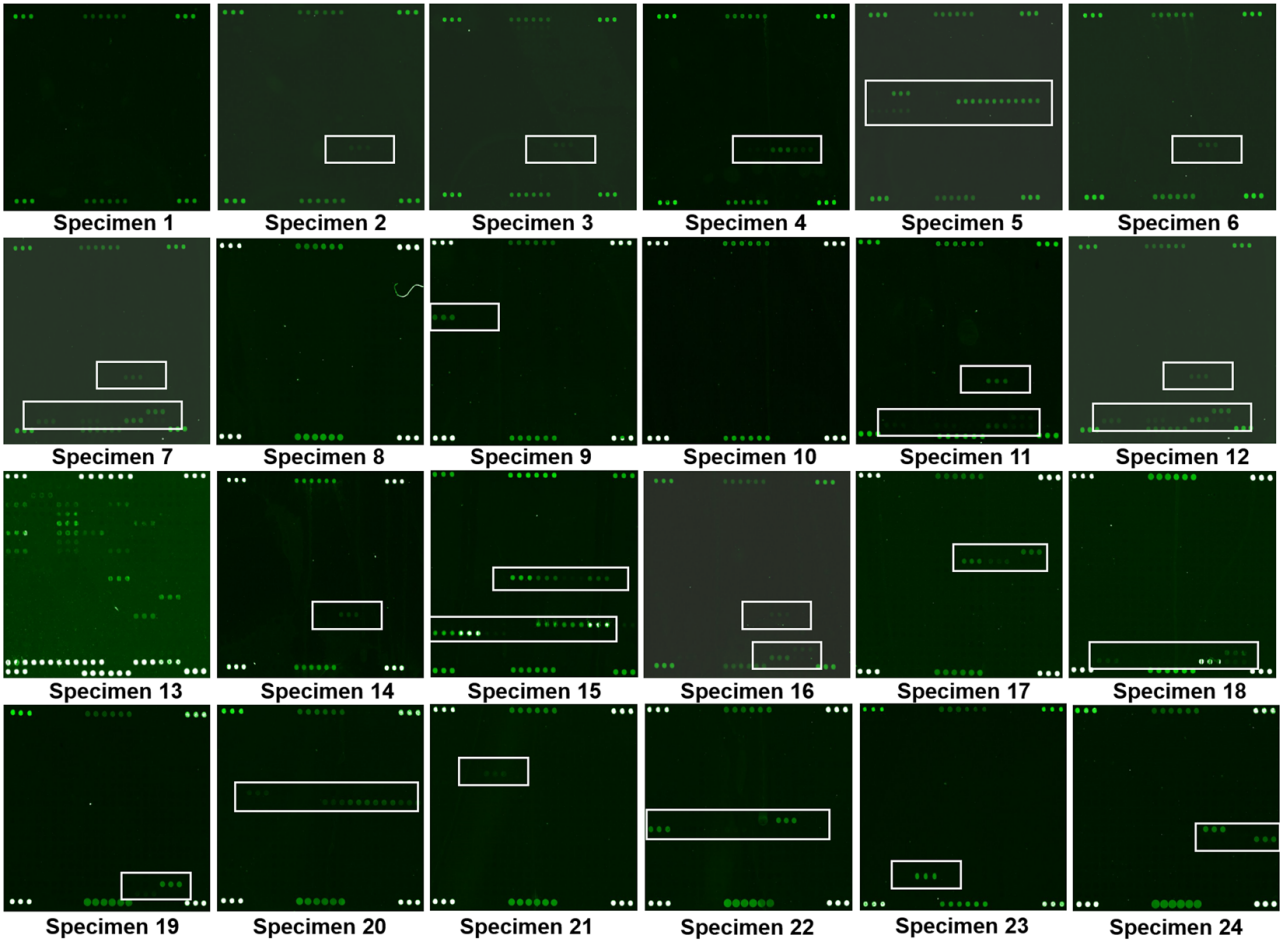
The results of microarray hybridization of 24 specimens from CSF. (**A**) There is a probe repeat for every 3 spots and “or” relation between all probes for the same bacteria (multiple probes were used for detecting bacteria subtypes). So the signals of three spots (or its multiples) means the sample containing this target bacteria.

After gene amplification, twenty-four CSF specimens from patients were hybridized with microarray. A total of 21 positive and 3 negative identifications were obtained. Seven target pathogens were detected in the CSF specimens, of which *S.aureus* and *S.pneumoniae* occurred with the highest incidences. *S.aureus* was found positive in 11 cases, *S.pneumoniae* was detected in 7 cases, *E.coli* was positive in 4 cases, *CN-S* was positive in 3 cases, *N.meningitides* was positive in 2 cases. One was positive both for *H.influenza* and *S.epidermidis*. According to the results of microarray, those of one case suggested that 5 pathogens might co-exist in specimen 13, and *S.aureus* was presented in 5 cases along with *S.pneumoniae*, all these results revealed that the patients represented the multiple bacterial infection (Fig. 4, Table 3).

We compared the microarray results with the CSF culture results, nine consistent results and fifteen inconsistent results were observed. Among the inconsistent results, eight specimens were positive by the microarray, compared with the negative results by the culture test (specimen 2, 3, 6, 9, 14, 17, 21, 24); five specimens were presented with mixed pathogens (specimen 7, 11, 12, 13, 16), compared with the single pathogen identification by culture test (specimen 12 did not perform the culture test); two specimens were reported the negative results by the microarray, compared with the positive results by the culture test because *Listeria monocytogenes* and *Citrobacter freundii* were out of this microarray detection range (specimen8,10) (Table 3).

There were 87.5% (21/24) positive results in DNA microarray compared with the 58.3% (14/24) of the culture test. Of which 58.3% (14/24) of the patients with culture-confirmed purulent meningitis, 37.5% (9/24) patients who were not confirmed by culture test but were demonstrated by the clinical diagnosis and DNA microarray.

## Discussion

The accurate identification of the causative bacteria with purulent meningitis will contribute clinicians to make effective therapeutic decisions. Conventional diagnosis of bacterial infection mainly relies on culture-based test from the CSF. However, this approach might have some disadvantages with regard to desired rapidity and sensitivity^25,26^. It usually need 3~5 days or in some cases up to a week to obtain a positive culture result, even spend longer time with slowly growing organisms or fastidious microorganisms. Additionally, a bacteria may have multifold of phenotype by CSF culture and make the wrong diagnosis. So the cultivation of bacteria is not always successful under laboratory conditions in fact. Polymerase chain reaction (PCR) assay which is based on nucleic acid detection, is well konwn to be more rapid and sensitive method than cultivate assay for bacterial pathogen detection^27,28^. However, one-to-one pattern is not suitable for the routine analysis in clinical laboratories. It seems that it is impractical to use of different primers for different species if the specimens contain one or more pathogens. Nowdays, the DNA microarray technique have been used more and more medical field because its advantages of specificity, veracity and high efficiency^29^. The purpose of this study was to develop a rapid and sensitive DNA microarray for helping diagnosis of purulent meningitis, which can simultaneously detect 7 species pathogen bacterium only in one single experiment, by targeting the species-specific sequences in the 16S rRNA, gyrB, gsp and nuc.

Currently, the 16S rRNA gene of almost all bacterial pathogens were found in body fluids have been sequenced, achieving a preliminary identification of bacterial species based on the conservative nature of the 16S rRNA gene^30–32^. Primers of which recognize conserved DNA sequences of bacterial genes that encode ribosomal RNA (16S rRNA) was the most common bacterial of classification system^33^. But 16S rRNA conservative sequence has a certain limitation that it is unable to distinguish between closely related bacterial species because of the resolution problems at the genus and/or species level. Recent studies have appeared gyrB gene to be more reliable and precise than the 16S rRNA gene at discriminating between related bacterial species^34,35^. In addition, except 16s rRNA and gyrB, *Staphylococcus aureus* have two unique functional genes *gsp* and *nuc* which was usually using for species identification^36^.

3.5 × 10^1^ copy numbers DNA can be used to hybridize the microarray and show the signal obviously, it means total 250 ng DNA from CSF can be prepared for detection, and it would be adequate for detection of bacteria in CSF specimens because 85% CSF samples with bacterial infection contained more than 1000 CFU/mL^37^. Most studies designed only one or two probe to test the target bacteria in CSF and work on without closely related bacterial species and complex envirment in CSF specimens^38^. So we designed the probes which detecting range containing all the sequences of the target genes in the Ribosomal Database Project (RDP), Silva database, and GenBank database for one bacteria and it was successful in identifying mixtures of organisms in polymicrobial meningitis.

We evaluated the utility of this DNA microarray in 24 CSF specimens. There was 87.5% (21/24) positive results in DNA microarray compared with the 58.3% (14/24) of the culture test. Of which 58.3% (14/24) of the patients with culture-confirmed purulent meningitis, 37.5% (9/24) patients who were not confirmed by culture test but were demonstrated by the clinical diagnosis and DNA microarray. The lower sensitivity of culture may be explained by previous antibiotic usage prior to lumbar puncture, especially the patients were given intravenously or intramuscularly^39^. Multiple bacterial infections were detected in 5 cases by the microarray compared with the single bacterial infection by culture test. Interestingly, the four CSF specimens were all reported the *S.pneumoniae* by the cultule assay. We guessed the *S.pneumoniae* suppressed non-competitive bacteria growth by the secretions when they were co-cultured. This result suggested the presence of multiple bacterial infections and mislead the therapeutic decision regarding to which the culture test should be improved and the potential availability of microarray. *S.aureus* and *S.pneumoniae* occurred with the highest incidences (Table 3 and Fig. 4). Specimen 6 and Specimen 17 were from the same patient whom diagnosed purulent meningitis and the first result by microarray test was *S.aureus*. Unfortunately, by using of drugs to cure, the second result by microarray test was *E.coli* (Fig. 4) after recurrence 4 months later. We think that microarray test can be better targeted bacteria pathogens even if the patients were given antibiotic drugs. The results of this study revealed that the microarray assay provided us a higher sensitive method and positive predictive value compared with the culture-based test for bacterial infection. Additionally, it just need 24 h to obtian the result by microarray assay.

In our study, although the lower sensitivity of CSF culture, the specimen 8 and specimen 10 were reported the *Listeria monocytogenes* and *Citrobacter freundii* by the culture test, respectively. In contrast, the DNA microarray showed negative results because of lacking species-specific probes for those two pathogens on the microarray (Table 3 and Fig. 4). It suggested that the species-specific probes were important for bacterial identification so that the microarray can’t perform its advantages in some cases. Therefore, we will update the probes of the chip in order to identify more bacteria pathogens. At present, it should be better to combine CSF cluture with micromarray as the stronger tool for diagnostic accuracy of pathogens with purulent meningitis. Undeniably, the microarray method will replace the CSF cluture method gradually when we have a large number of CSF samples to “drow” a map of pathogenic bacteria of purulent meningitis spectrum.

Our study proved that the microarray technique presented the advantage better than conventional culture-based test for detecting pathogenic bacteria in CSF specimens. We will acquire more CSF specimens and design new more specific probes of pathogenic bacteria in our next work project. And with the new portable chip testing equipment developing by Department of Beijing National Biochip Research Center sub-center in Ningbo, samples can be achieved in situ detection. At the same time, we will develop a new detective microarray based on the principle of microfluidic technology which can detect CSF specimen directly without DNA preparation, make further improvement of the detection speed. In conclusion, the microarray technique becomes a valuable tool for detecting meningitis bacteria pathogens in CSF samples, especially for detecting other clinical organisms, but this technique requires further development.

## Electronic supplementary material

supplementary information and data
