# Supplementary material for: Rapid pathogen identification using a novel microarray-based assay with purulent meningitis in cerebrospinal fluid
Source: Sci Rep. 2018 Oct 29;8:15965. doi: 10.1038/s41598-018-34051-0 (PMC6206030; doi:10.1038/s41598-018-34051-0)
Supplement: Supplementary file 1 — supplementary information and data [file 41598_2018_34051_MOESM1_ESM.pdf]

Title Page

Running title: DNA microarray identified pathogens of Purulent meningitis

Title: Rapid pathogen identification using a novel microarray-based assay with Purulent meningitis in Cerebrospinal Fluid

First Author and co-first author#: Yuting Hou，Xu Zhang#

Order of Authors: Yuting Hou [1,3], Xu Zhang[2,#], Xiaolin Hou[4], Ruofen Wu [1,3], Yanbai Wang [1,3], Xuexian He [1,3], Libin Wang[2], Zhenhai Wang[1,3,4]*

All Authors`s affiliations:

1 Department of Cerebrospinal Fluid Laboratory, The General Hospital of Ningxia Medical University, Yinchuan, Ningxia 750004, China

2 Department of Beijing National Biochip Research Center sub-center in Ningxia, General Hospital of Ningxia Medical University, Yinchuan, Ningxia 750004, China

3 Ningxia Key Laboratory of Cerebrocranial Diseases, Ningxia Medical University, Yinchuan, Ningxia 750004, China

4 Neurology Center, The General Hospital of Ningxia Medical University, Yinchuan, Ningxia 750004, China

Corresponding Author: Prof. Zhenhai Wang, M.D. Tel.: +86(0951)6744025

Fax: +86(0951)4082981; E-mail(for work): wangzhenhai1968@163.com

Address: Add. 804 Shengli Street, Xingqing Area, Yinchuan Ningxia, P.R.China

Submit email: xuzhang1012@163.com

Manuscript Region of Origin: CHINA

Number of Words/Characters in abstract: 194/1169.

The contribution of each author for the project and production of the article:

Zhenhai Wang: designed project, revised article, and coordinated all aspects of work.

Yuting Hou: participated in clinical experiment, steered chemotherapy and analyzed clinical material, wrote article of clinical part, Prepared figure 1.

Xu Zhang: designed genechip project, participated in genechip and molecular experiment, wrote article of genechip part, Prepared figure 2-4, table 3.

Xiaolin Hou: Designed and screened the probes, Tested the specificity and accuracy of probes, Prepared table 1-2.

Ruofen Wu: collected clinical cases, sorted the dates of clinical.

Yanbai Wang: Standard strains micromarry experiment, DNA Extraction.

Xuexian He: analyzed molecular material, prepared reagent and instrument.

Libin Wang: Optimized the conditions and parameters of microarray preparation.

Acknowledgements: This study was funded by the National Natural Science Foundation of China (No. 31660030,81160151) . This work does not directly or indirectly have any formal relationships with business groups.

Specimen 1 (genechip)

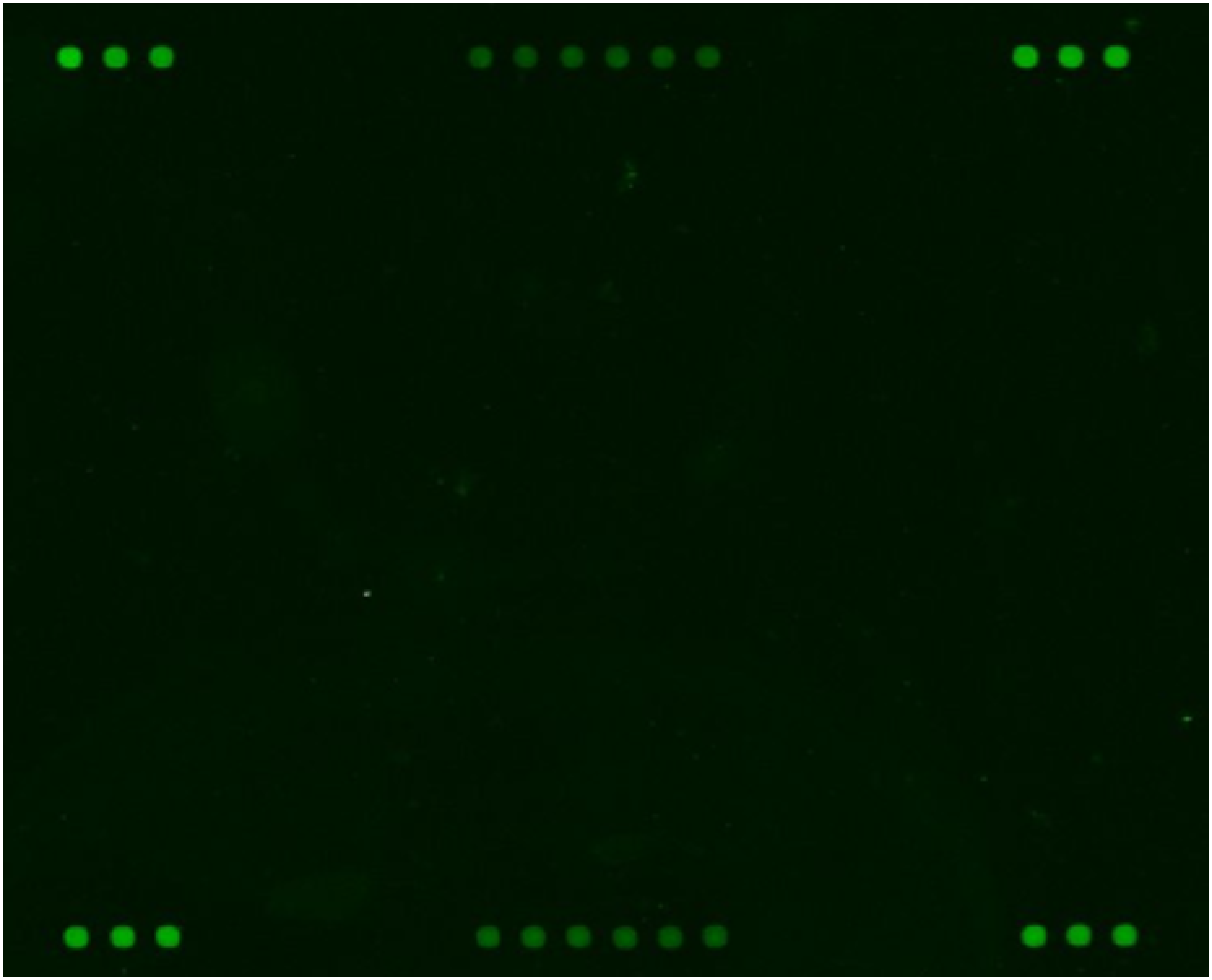

Specimen 2 (genechip)

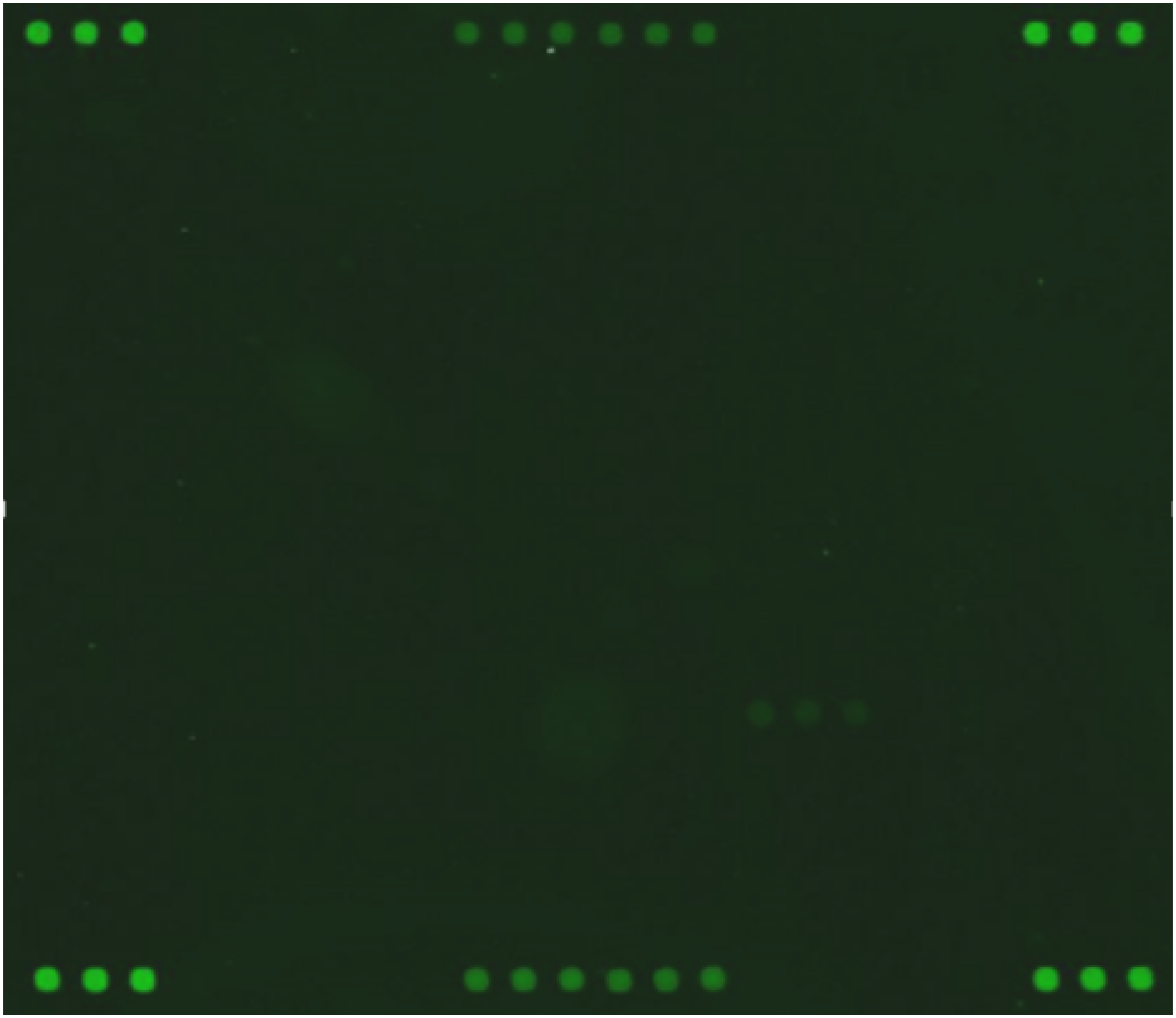

Specimen 3 (genechip)

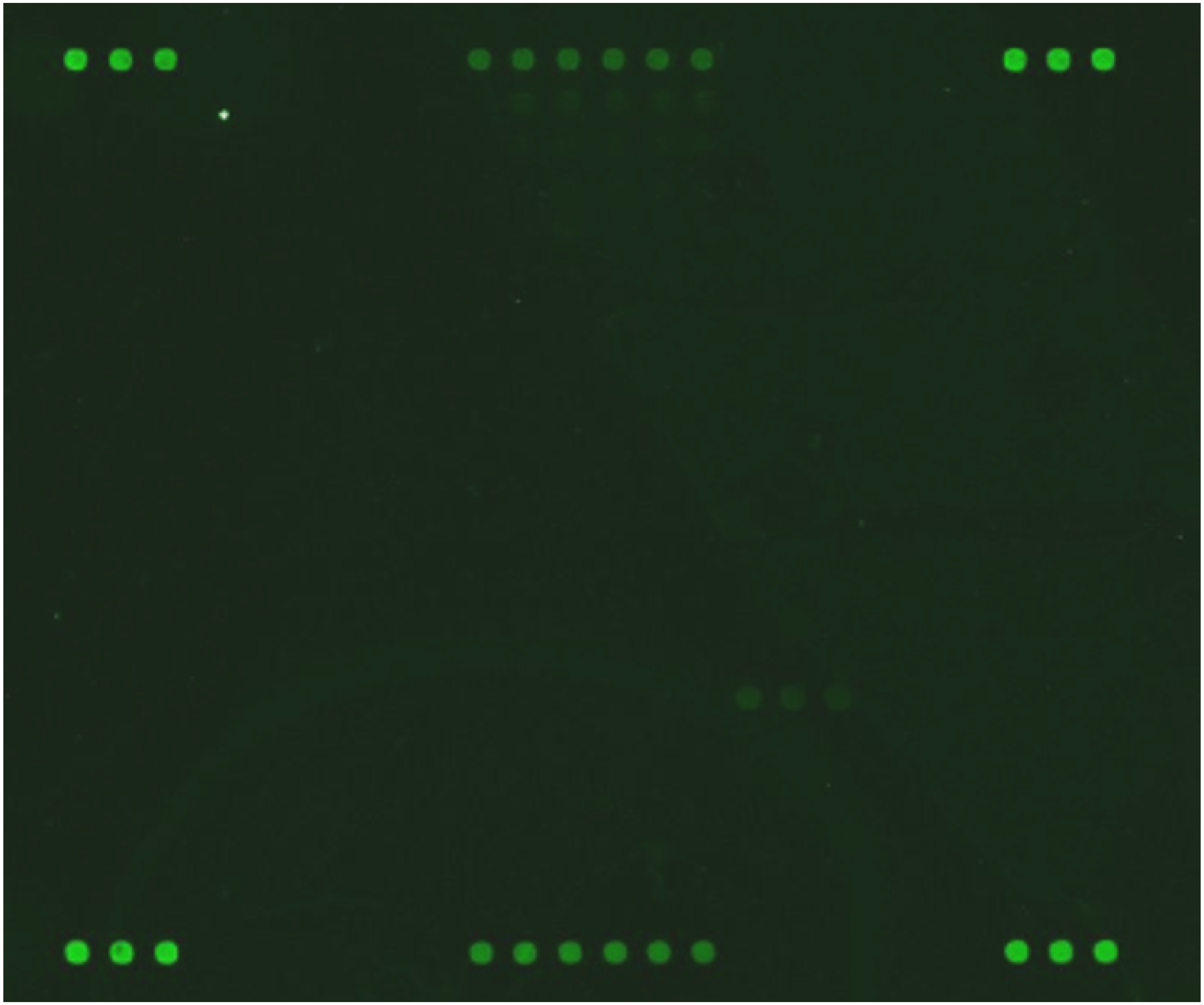

Specimen 4 (genechip)

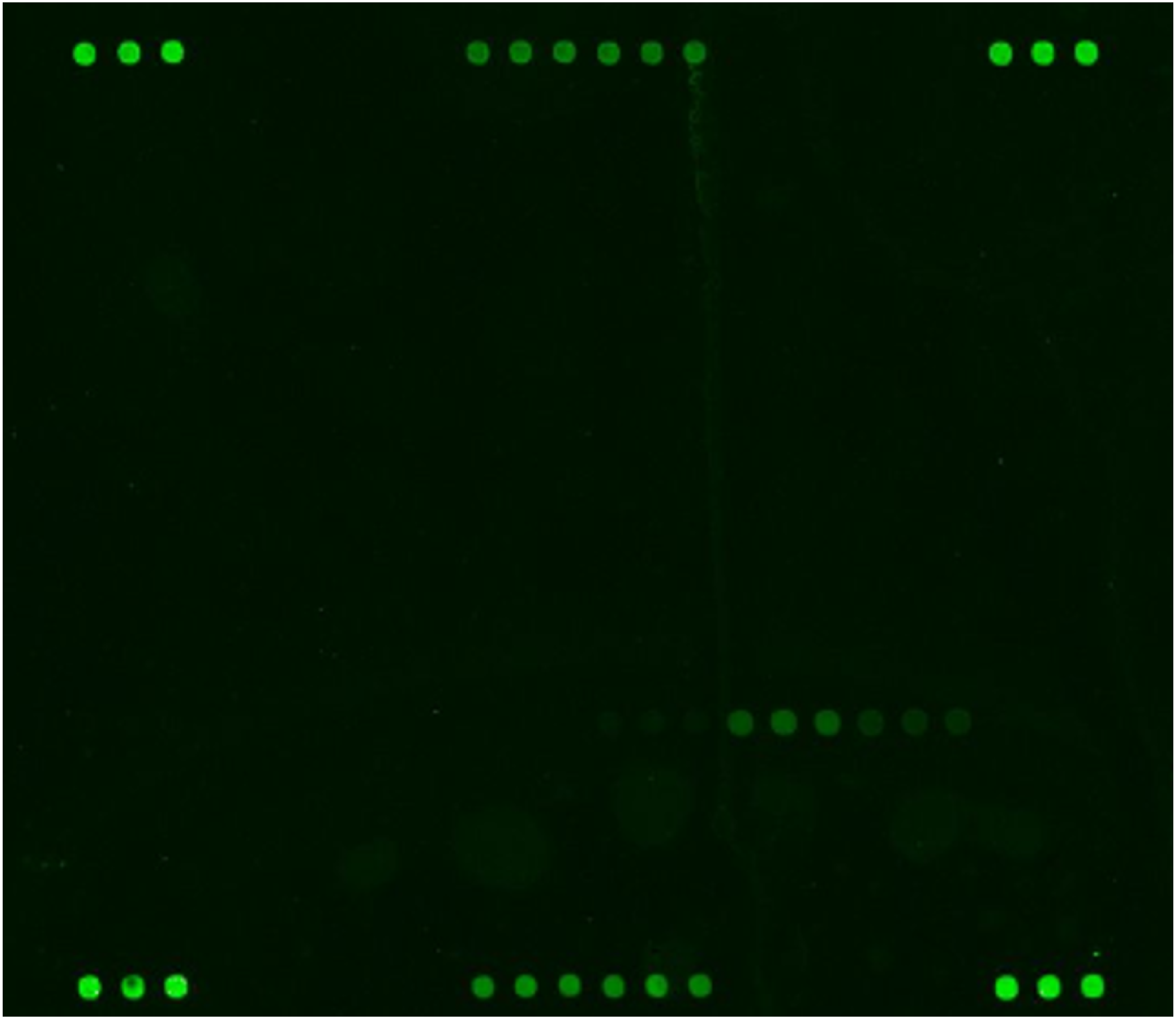

Specimen 5 (genechip)

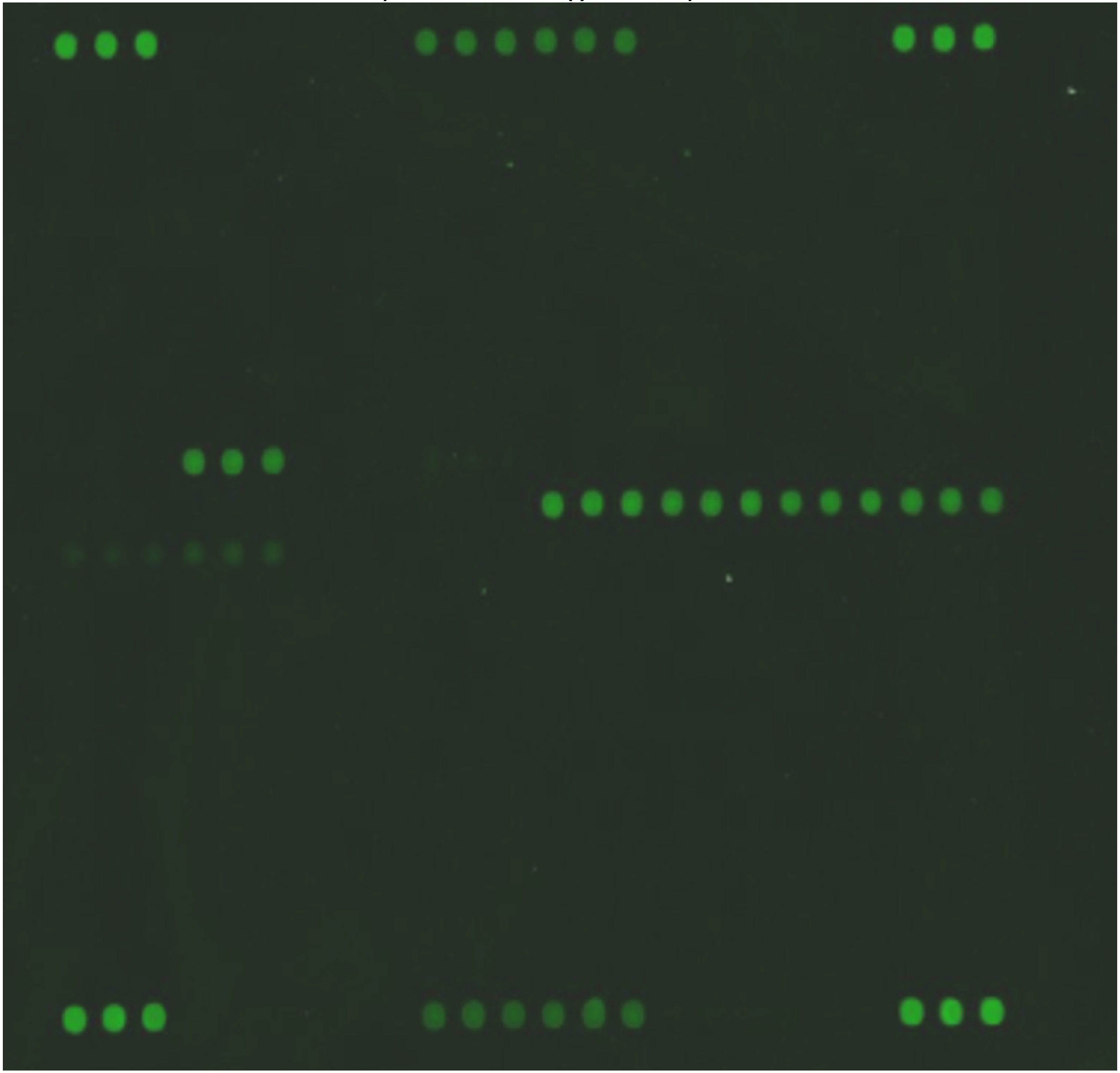

Specimen 6 (genechip)

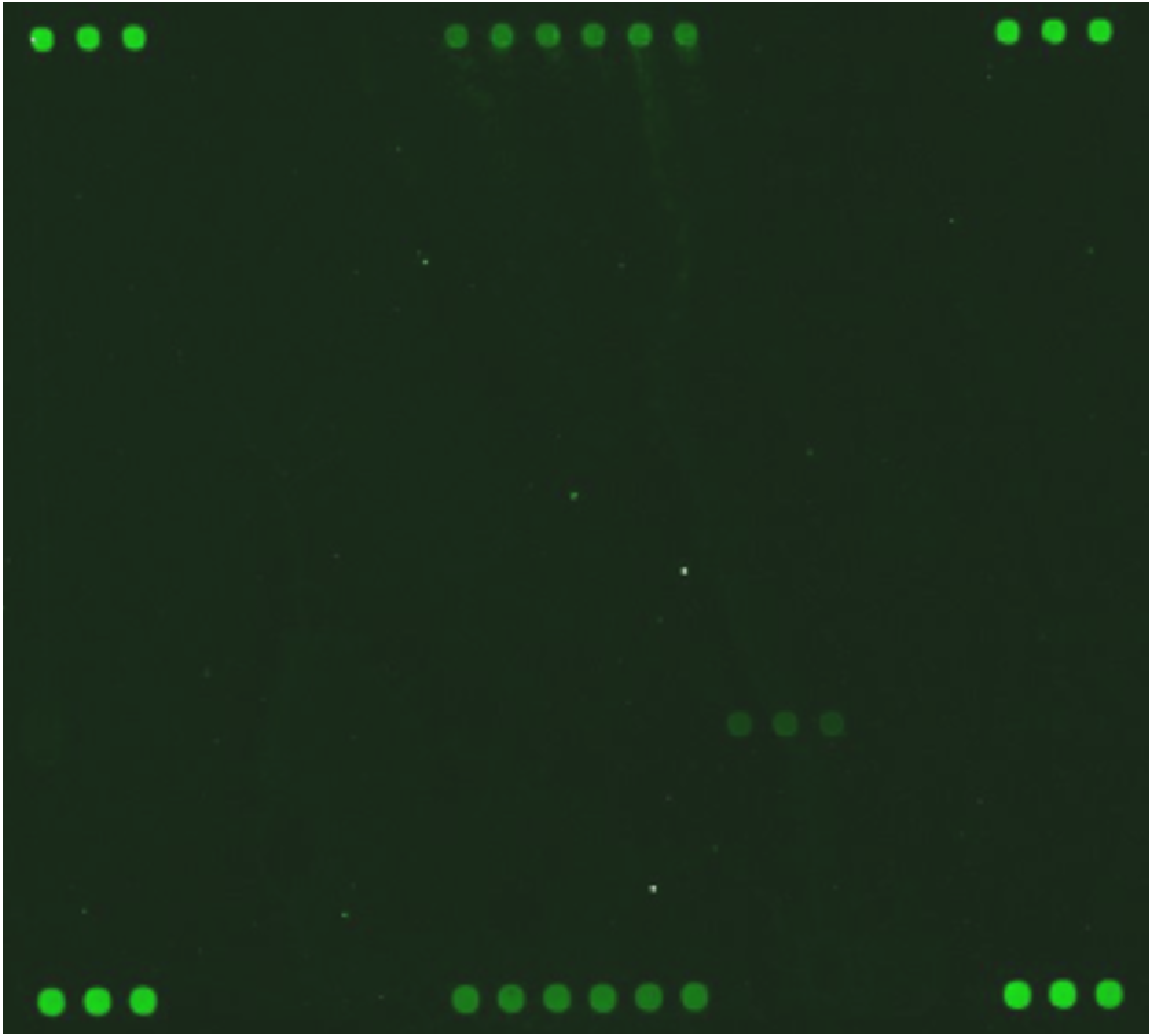

Specimen 7 (genechip)

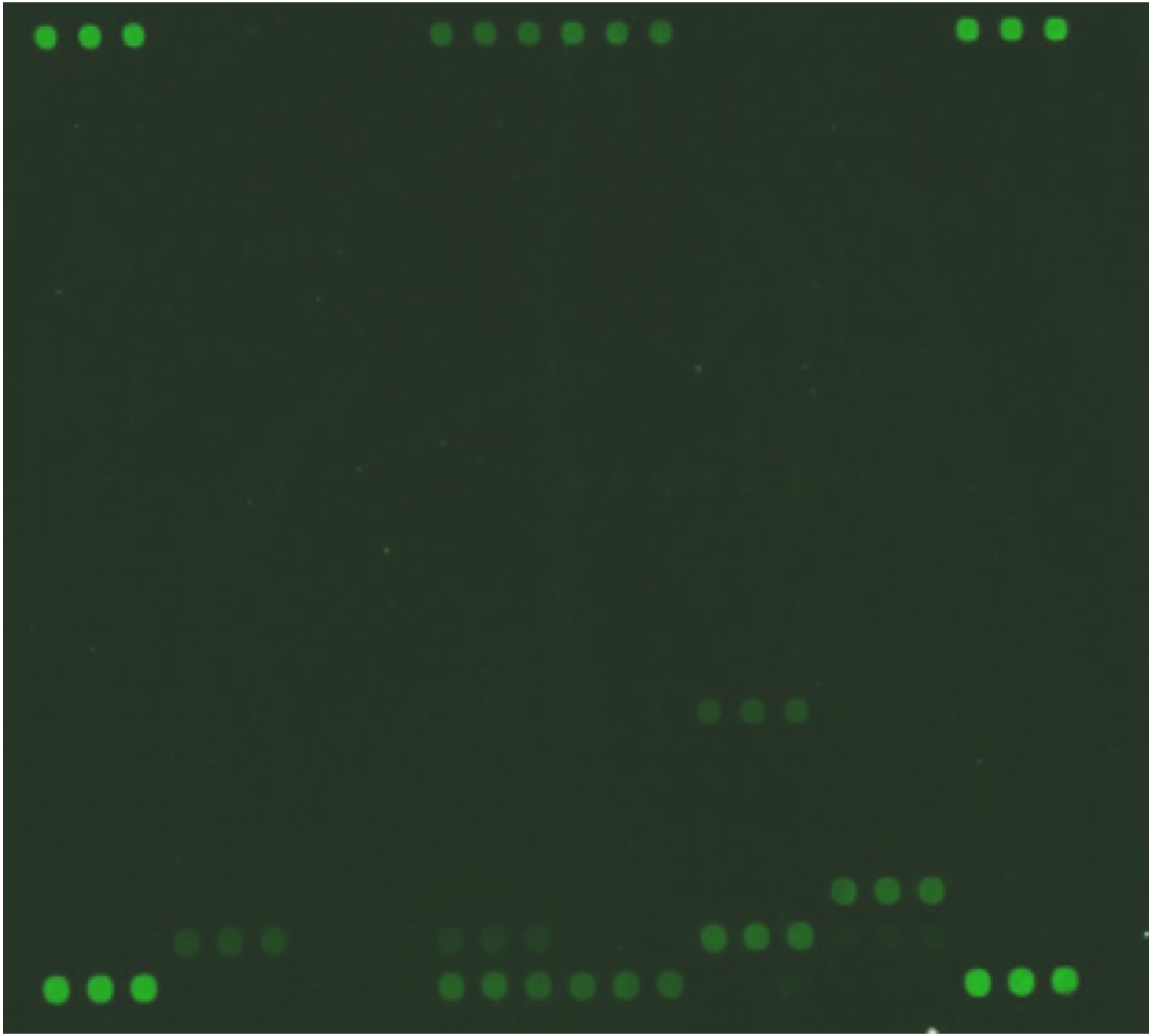

Specimen 8 (genechip)

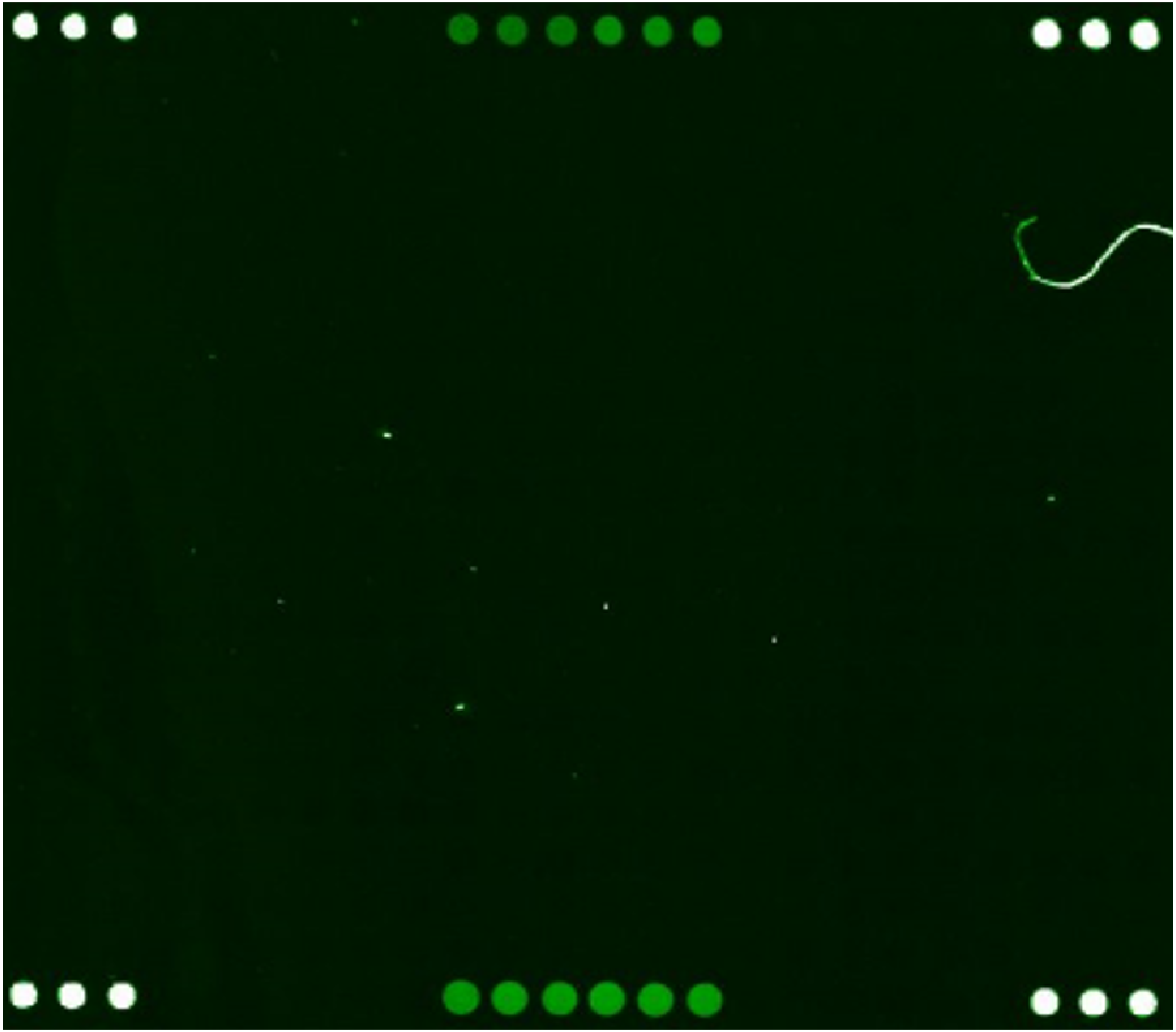

Specimen 9 (genechip)

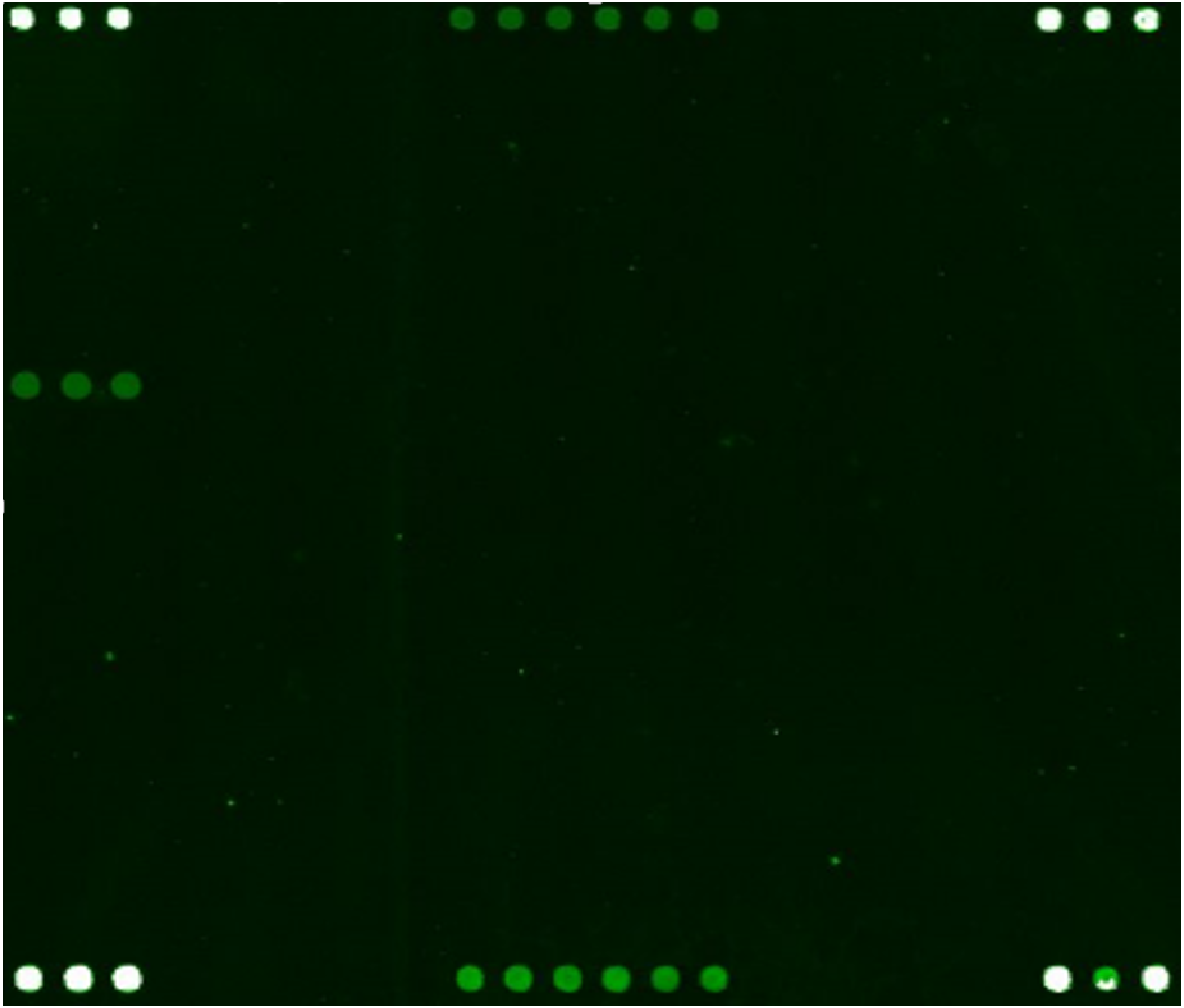

Specimen 10 (genechip)

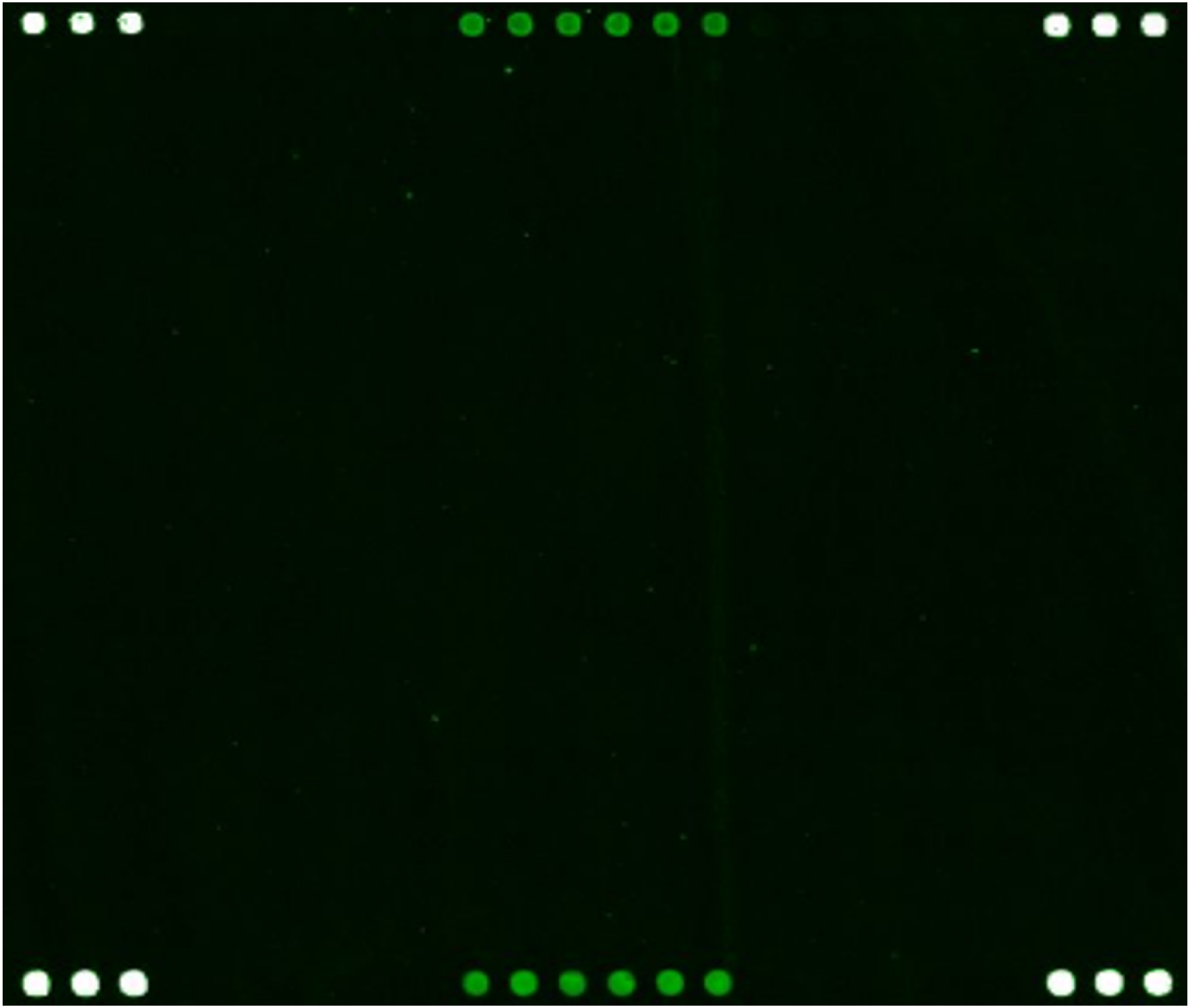

Specimen 11 (genechip)

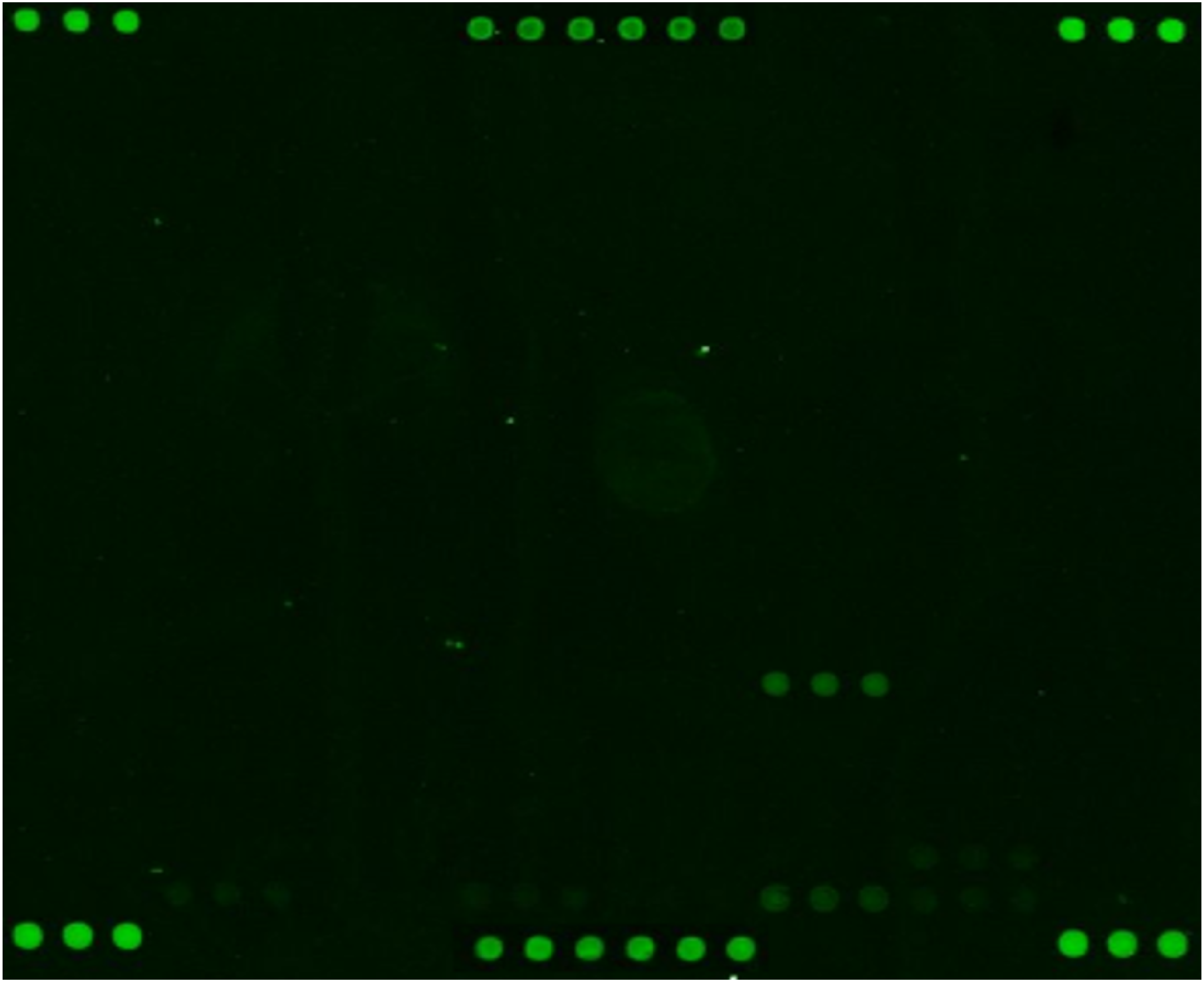

Specimen 12 (genechip)

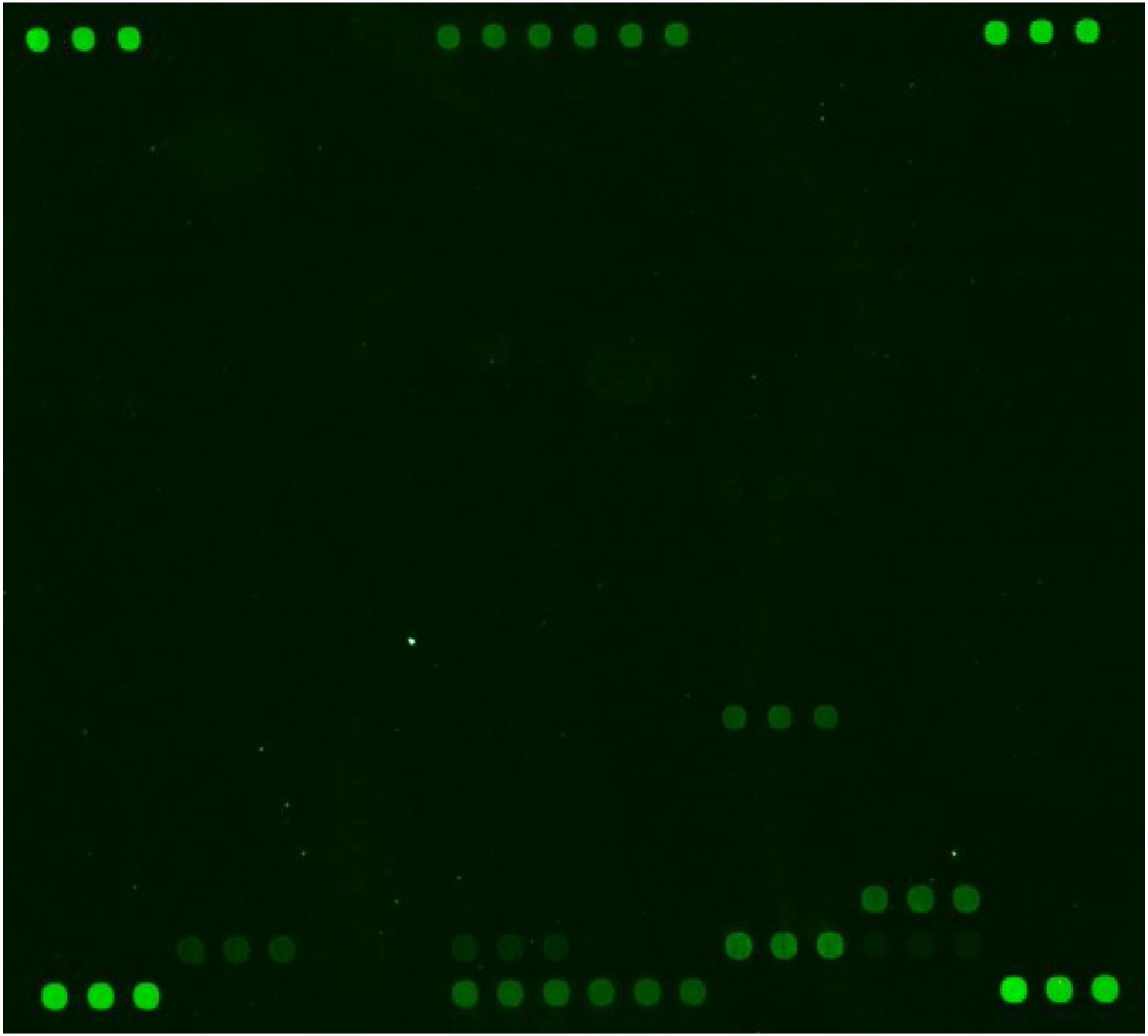

Specimen 13 (genechip)

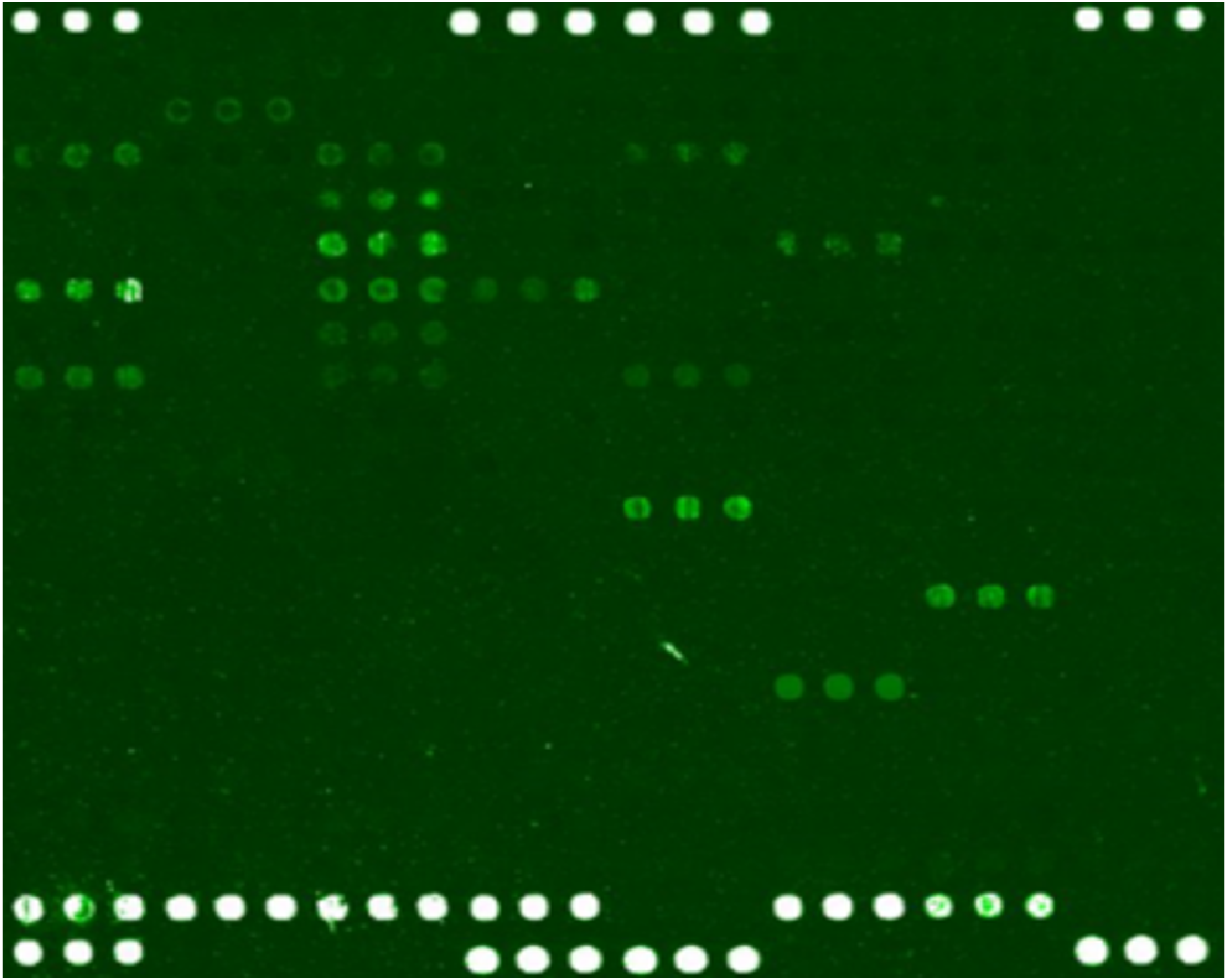

Specimen 14 (genechip)

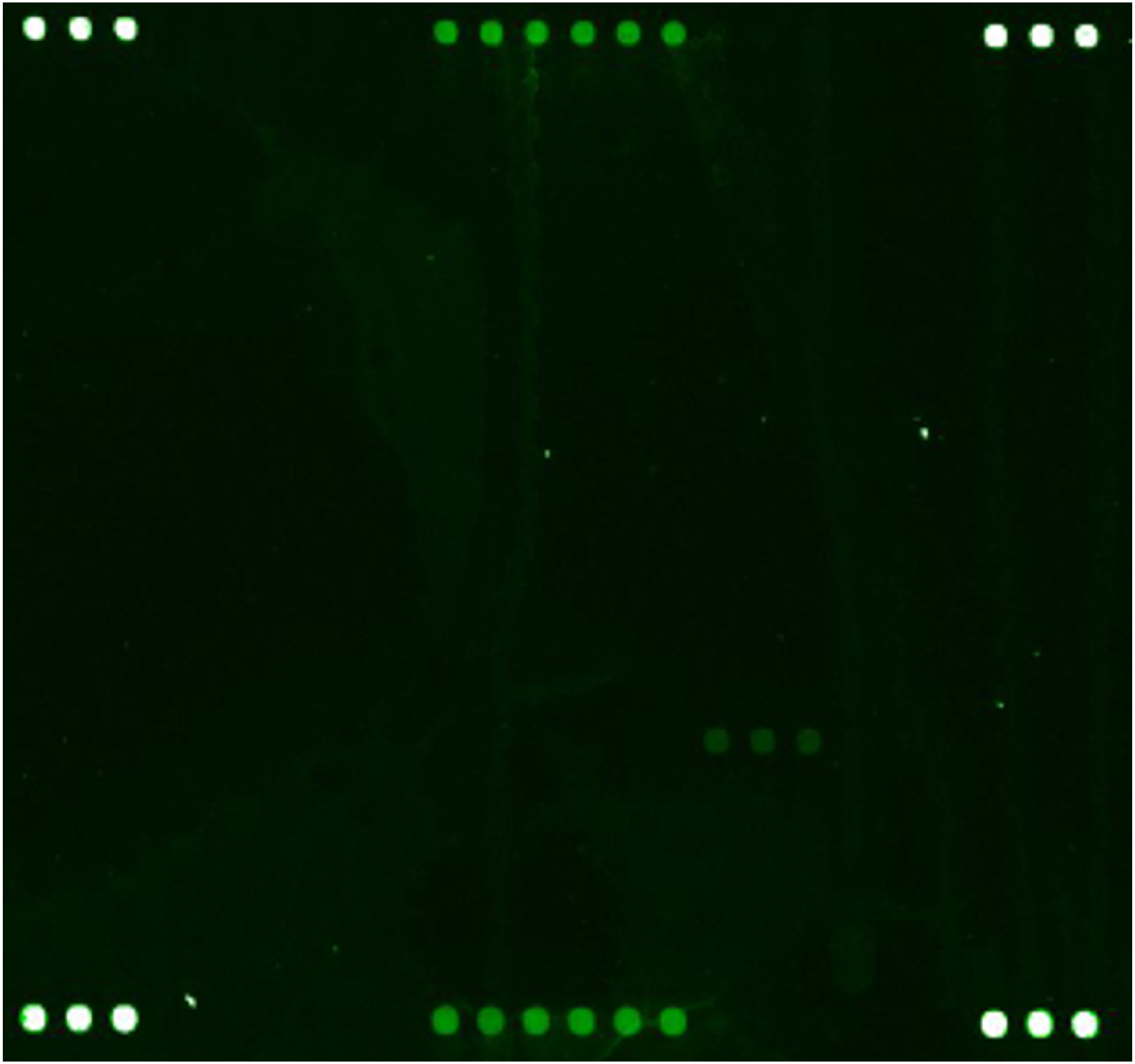

Specimen 15 (genechip)

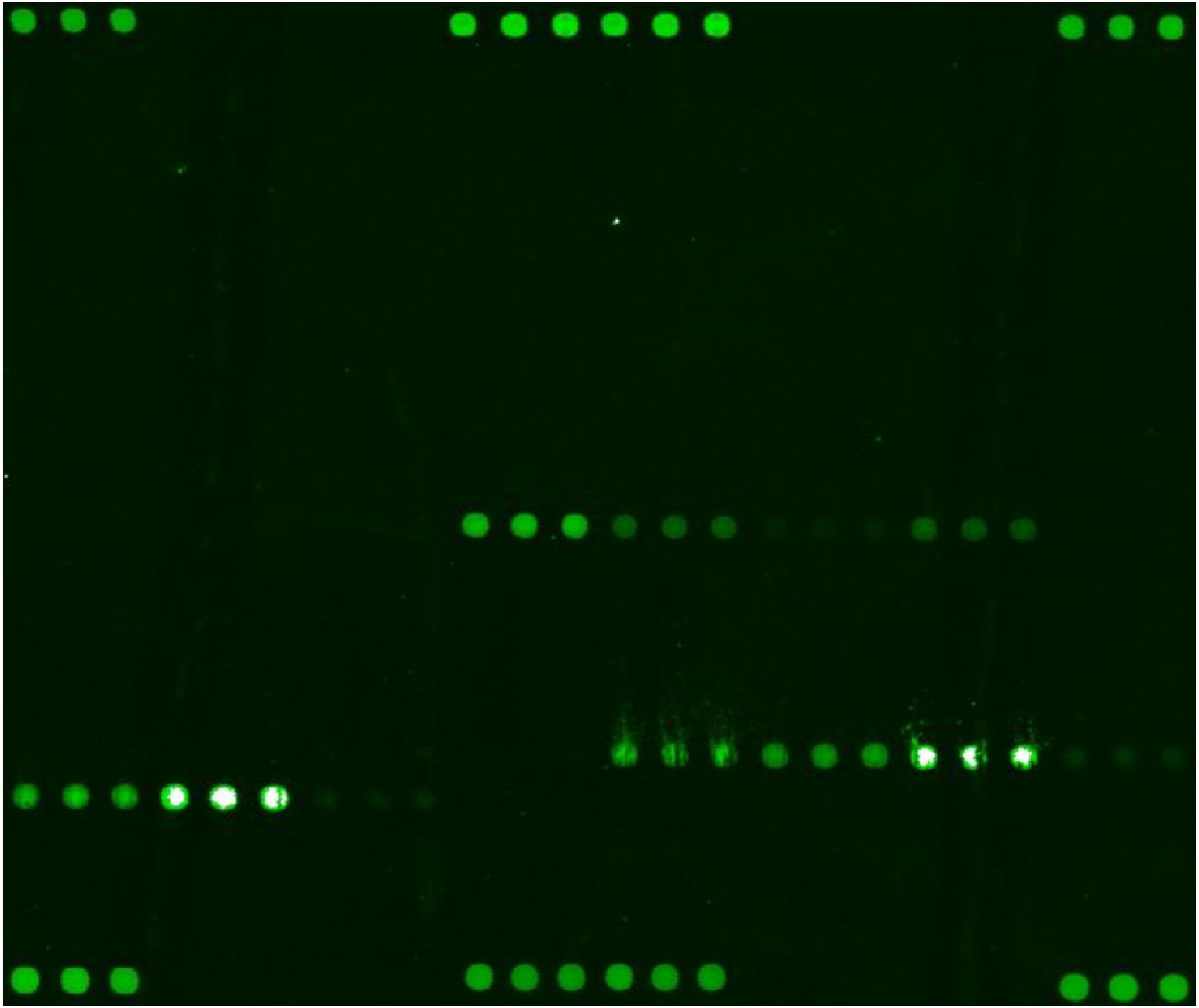

Specimen 16 (genechip)

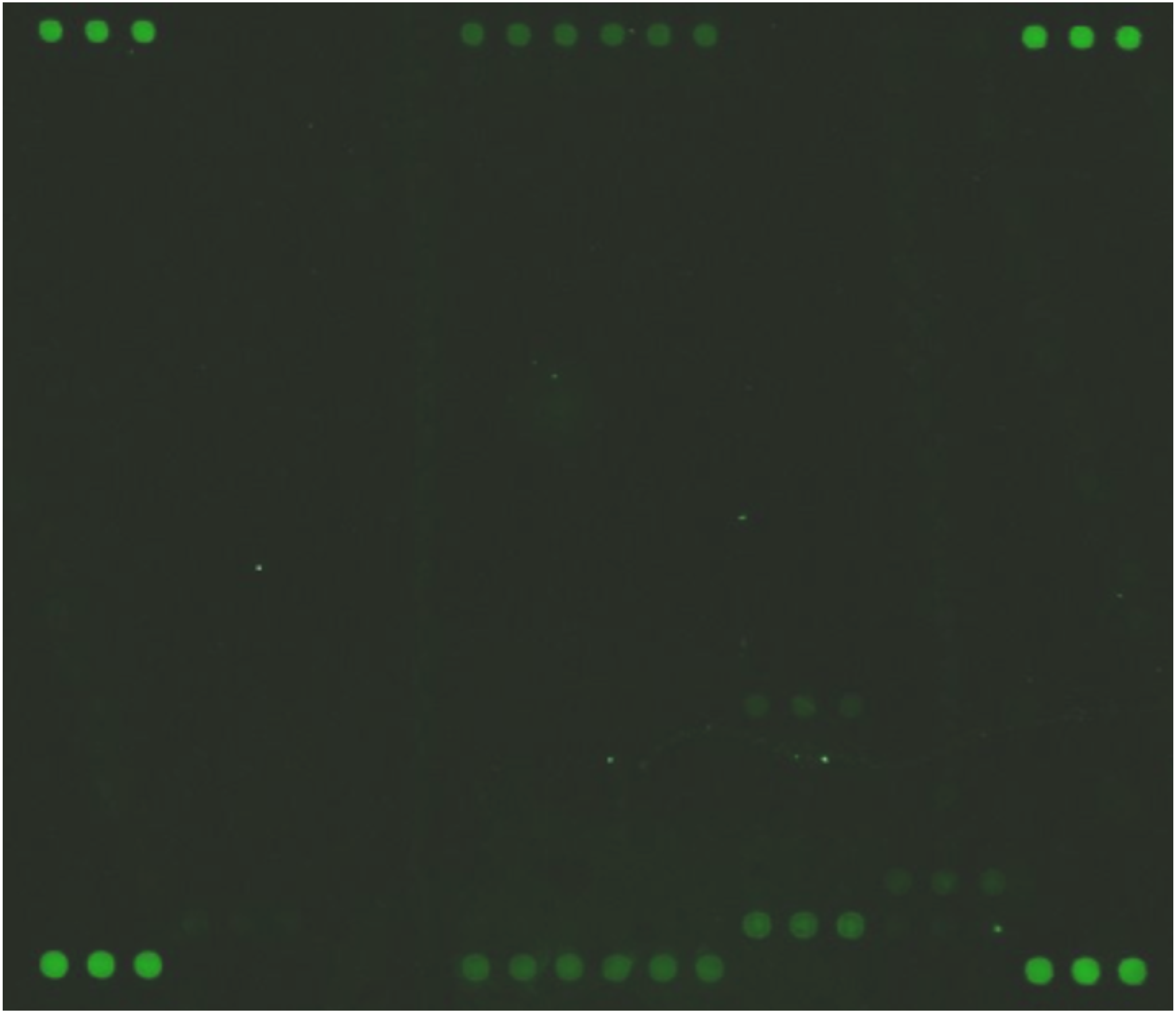

Specimen 17 (genechip)

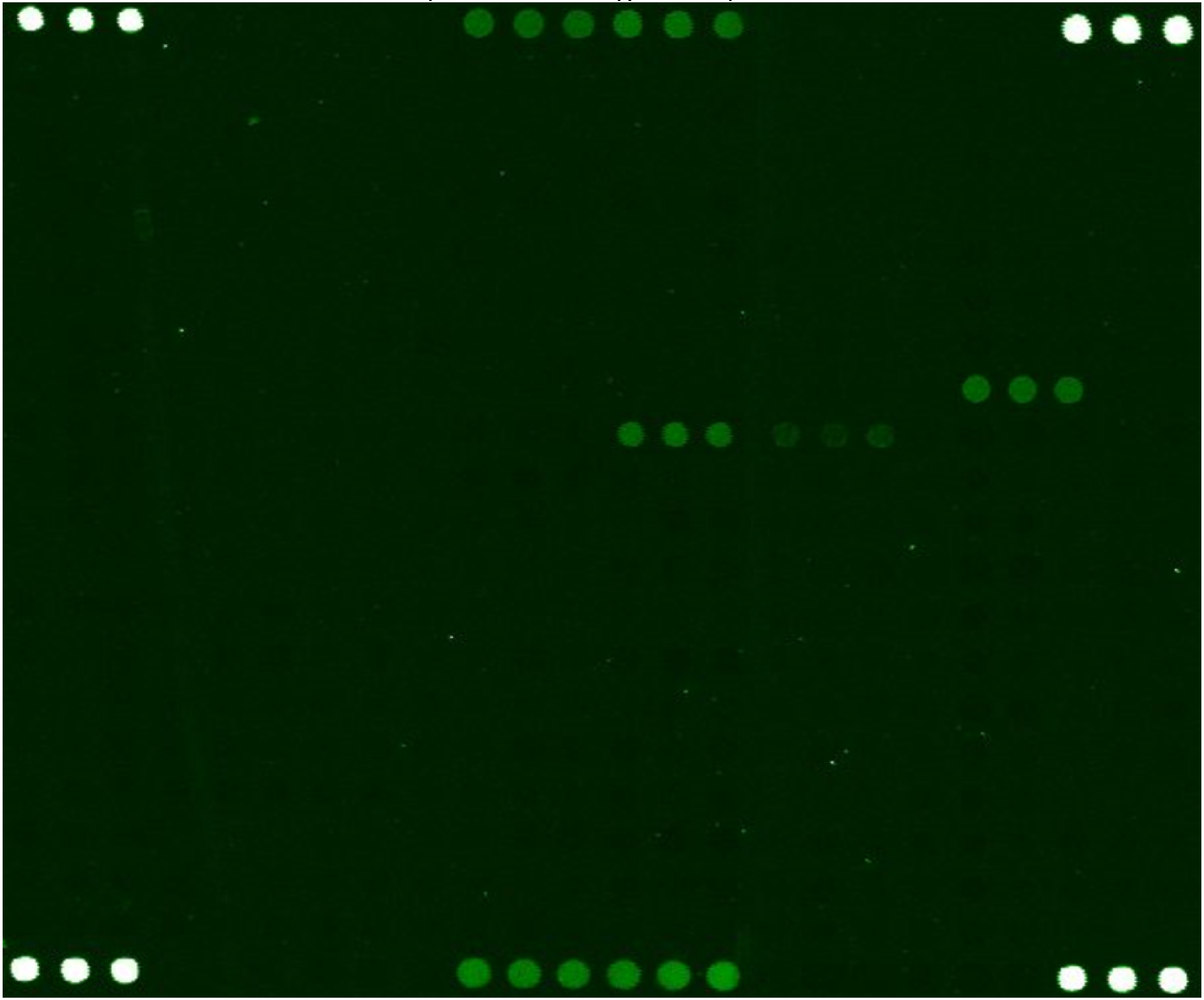

Specimen 18 (genechip)

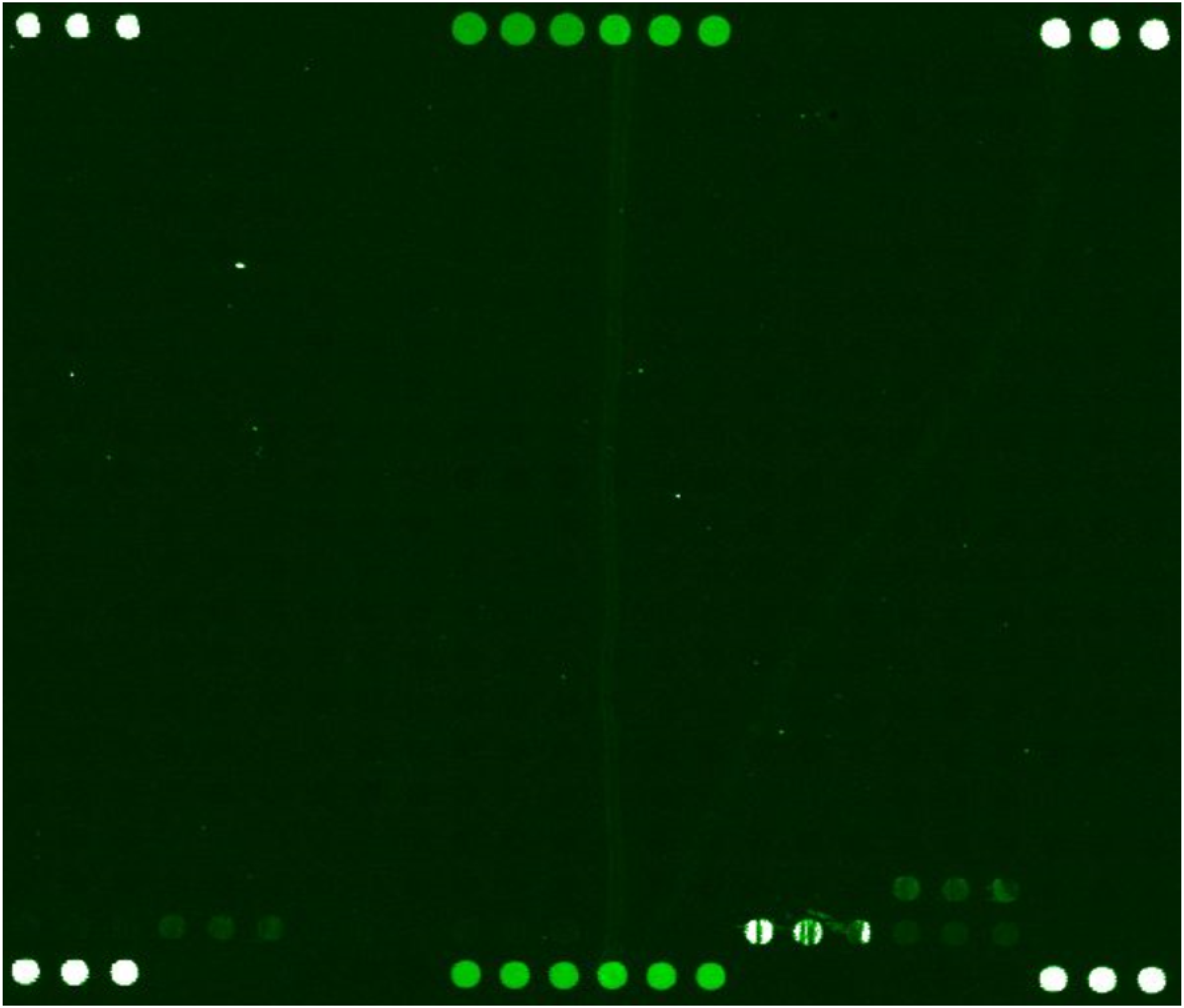

Specimen 19 (genechip)

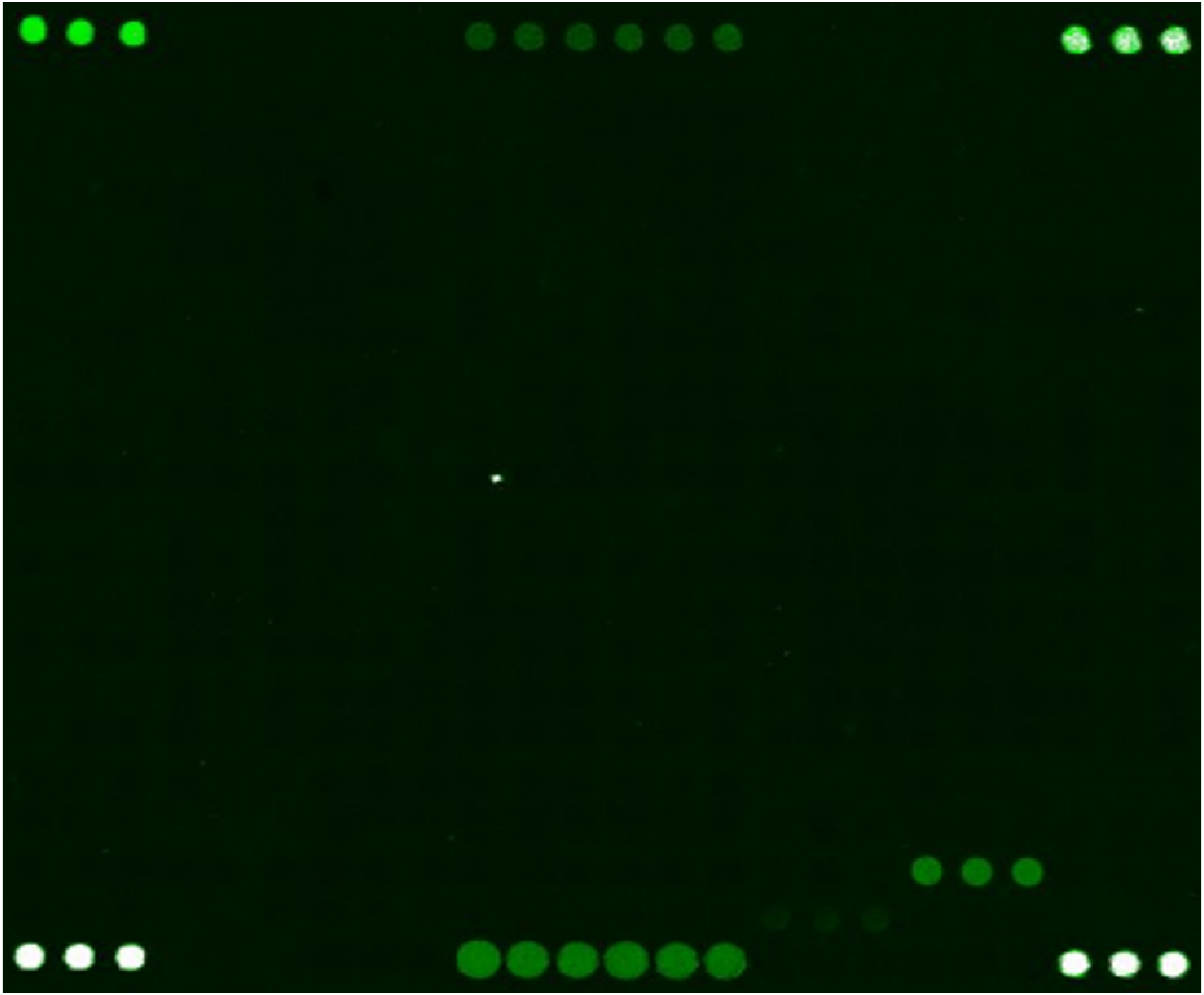

Specimen 20 (genechip)

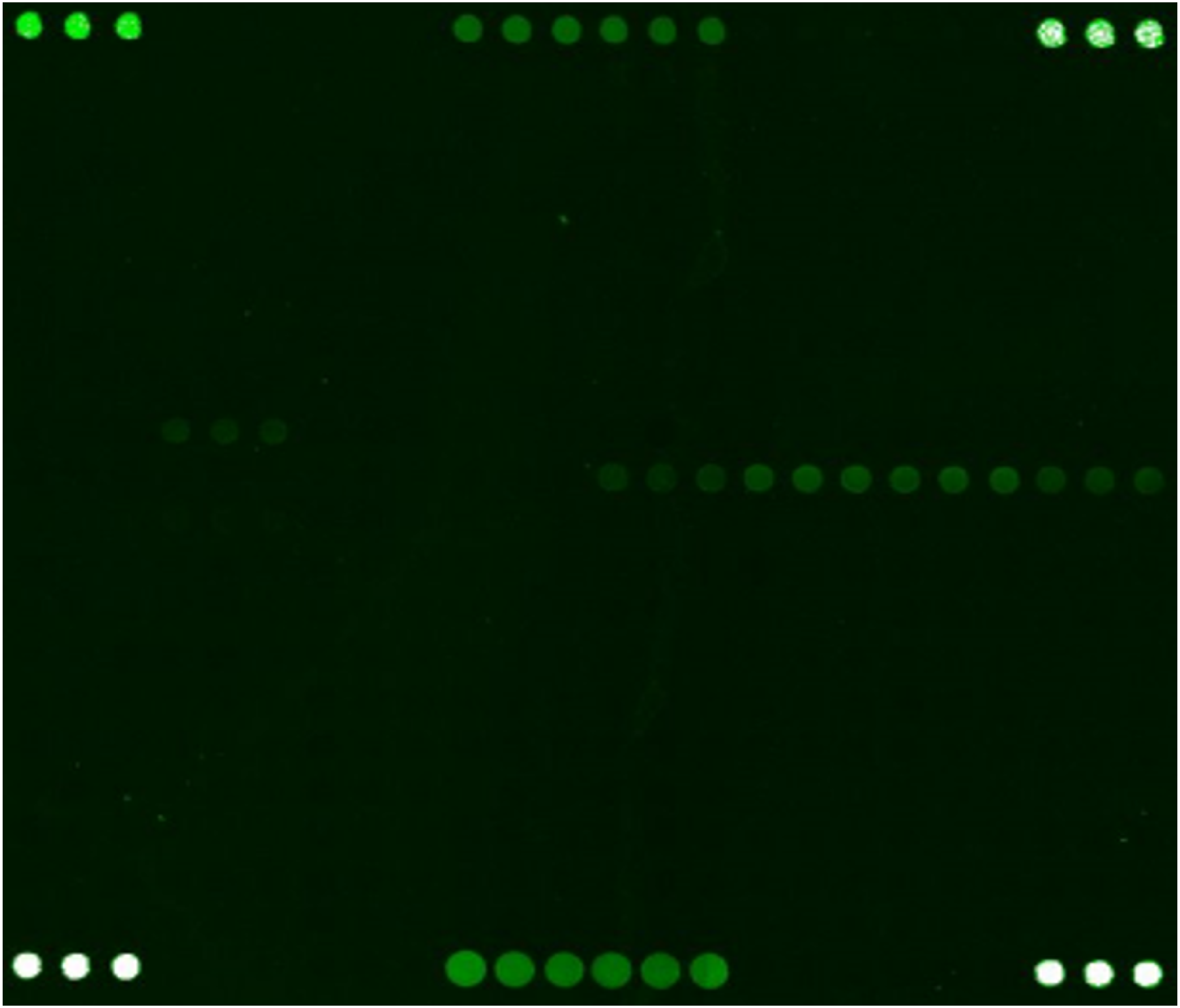

Specimen 21 (genechip)

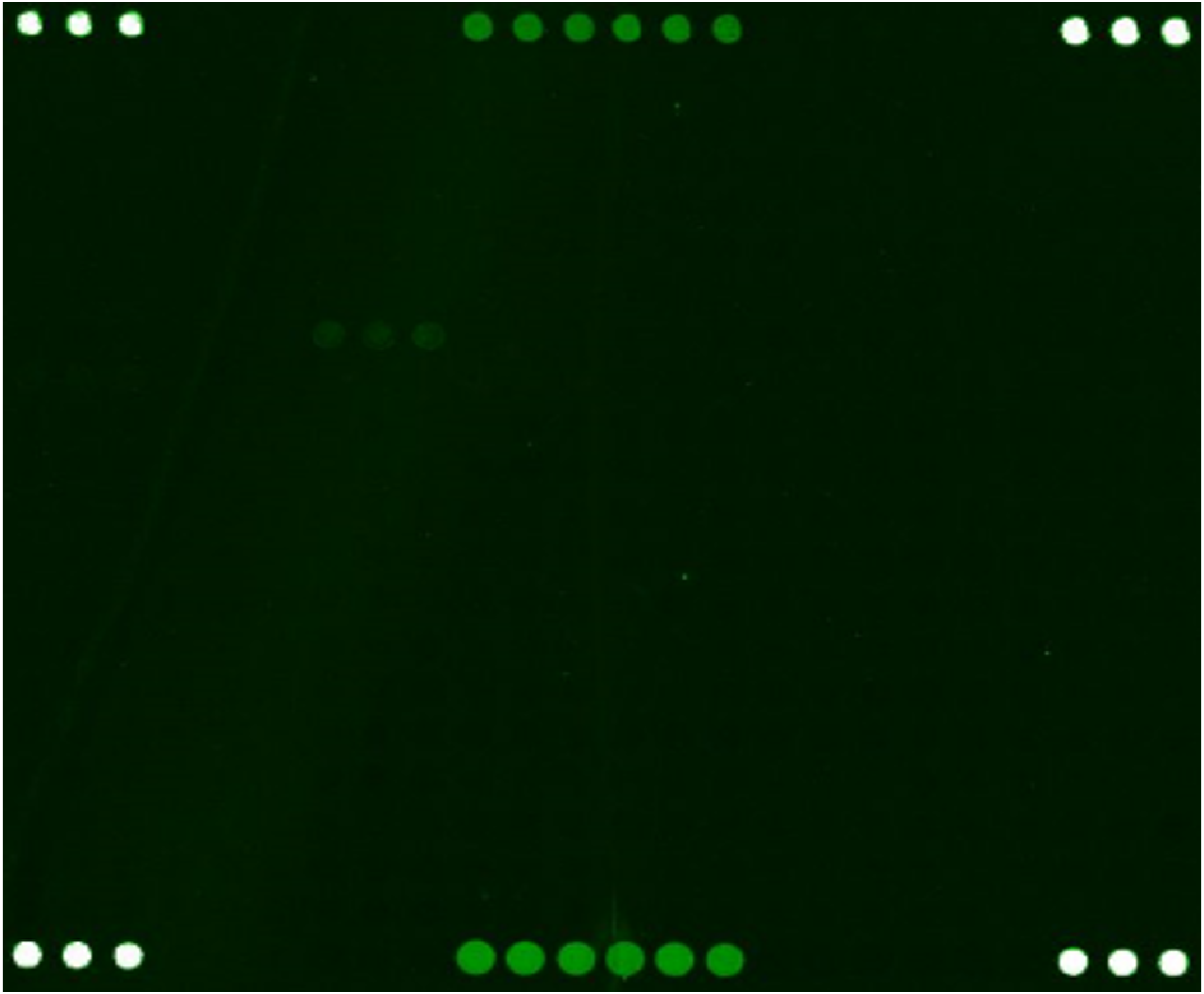

Specimen 22 (genechip)

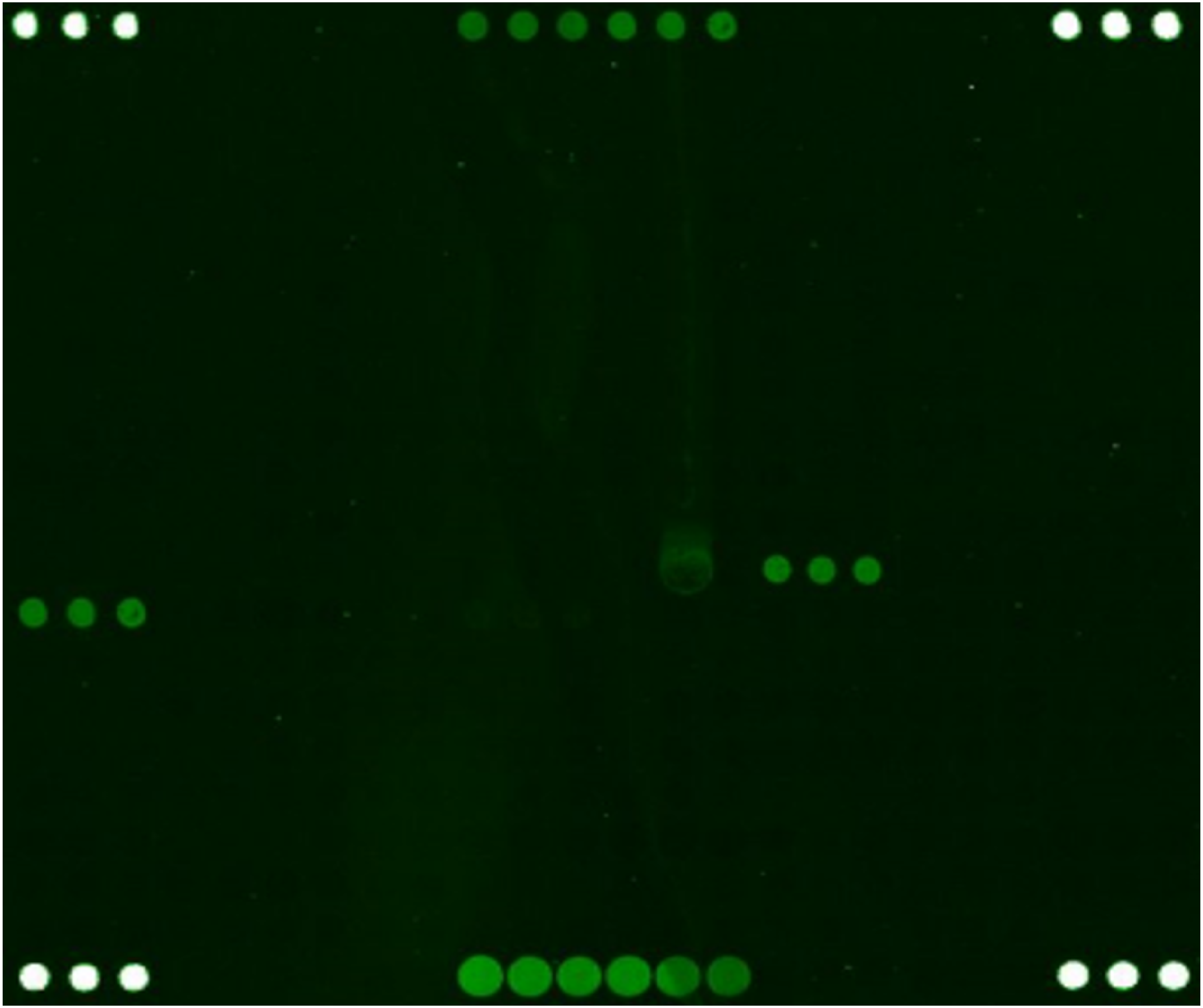

Specimen 23 (genechip)

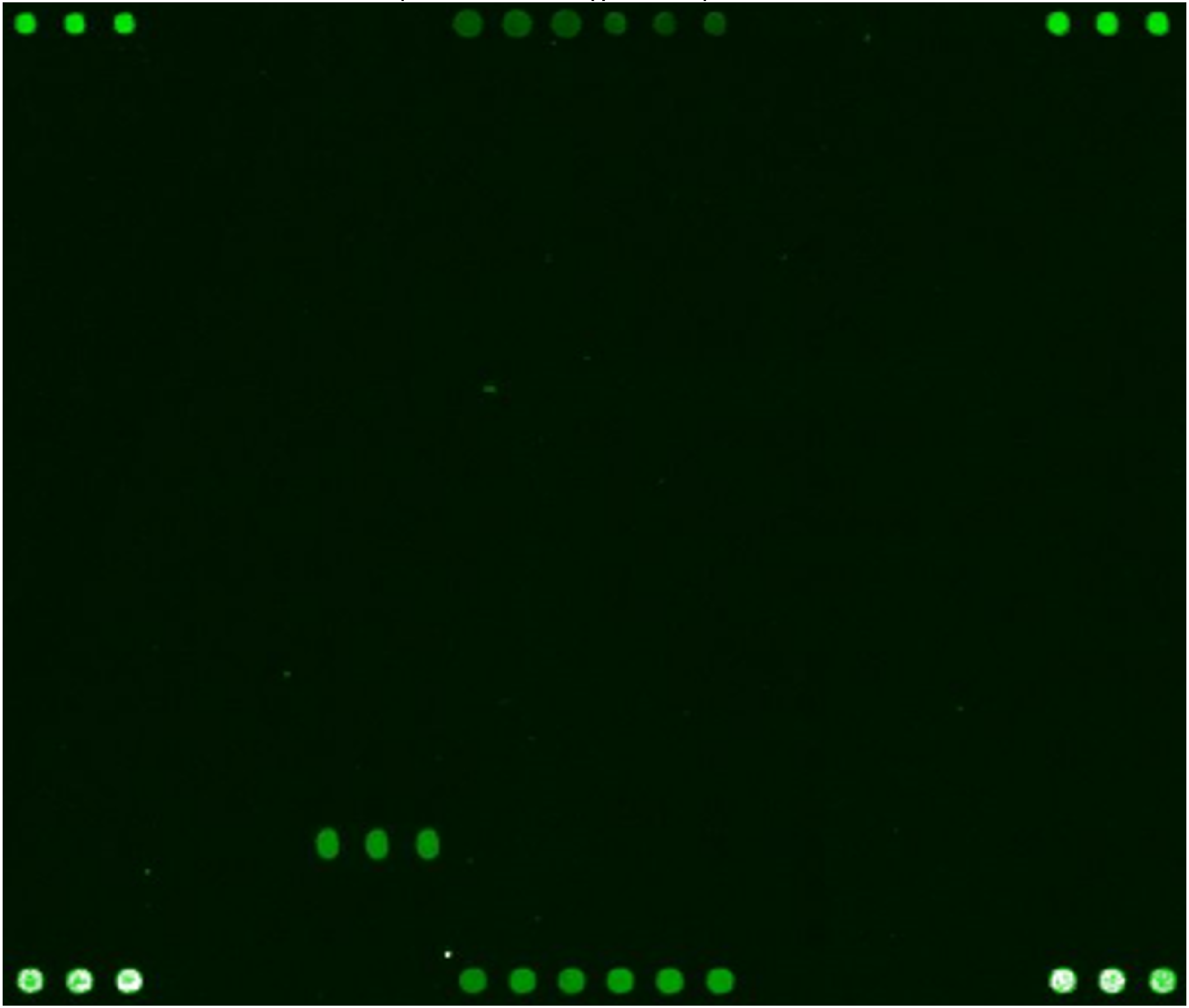

Specimen 24 (genechip)

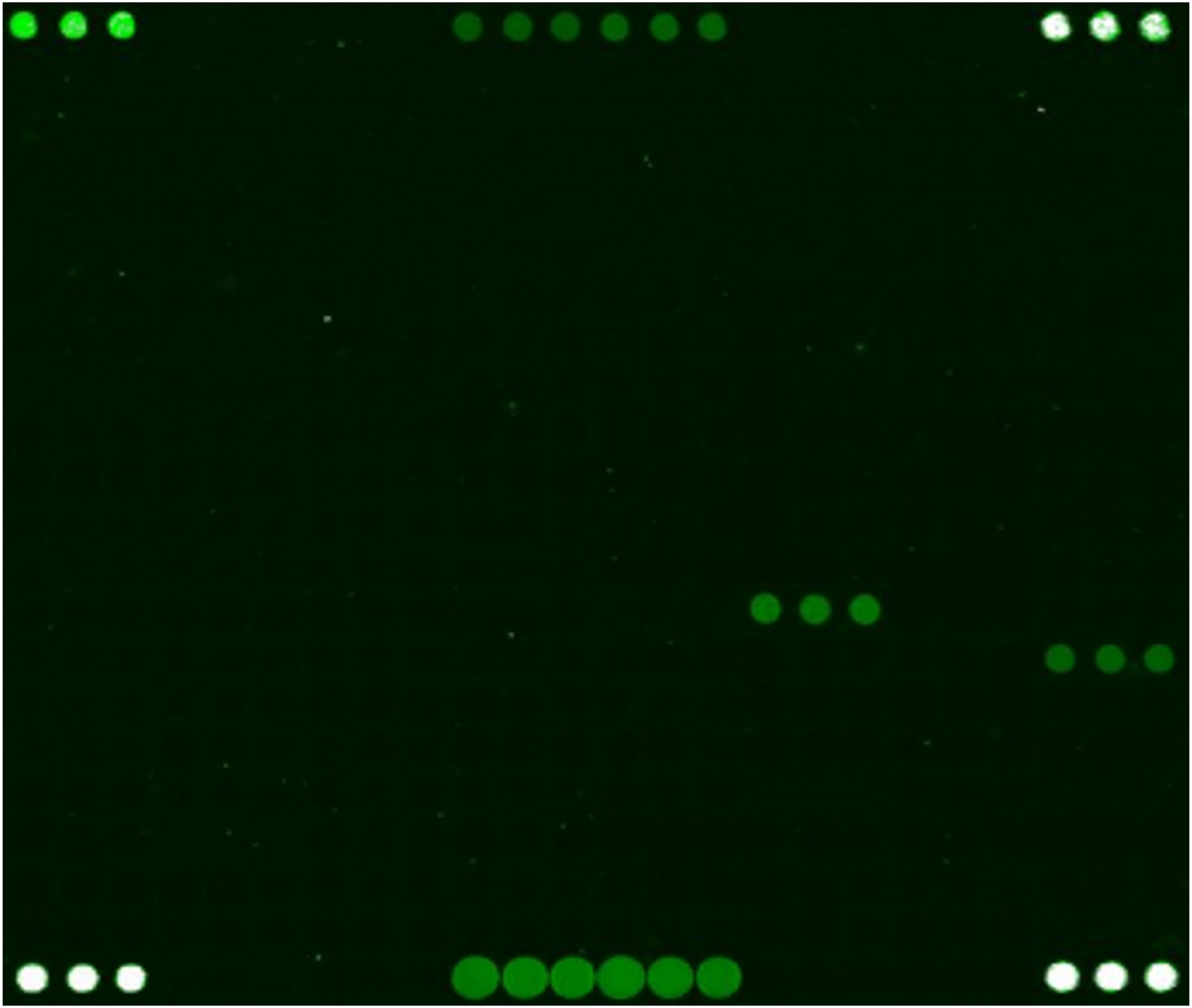

H.influenza standard strain 16s（2）N.meningitides 16s（3）and a specimen（4,5,6,7）

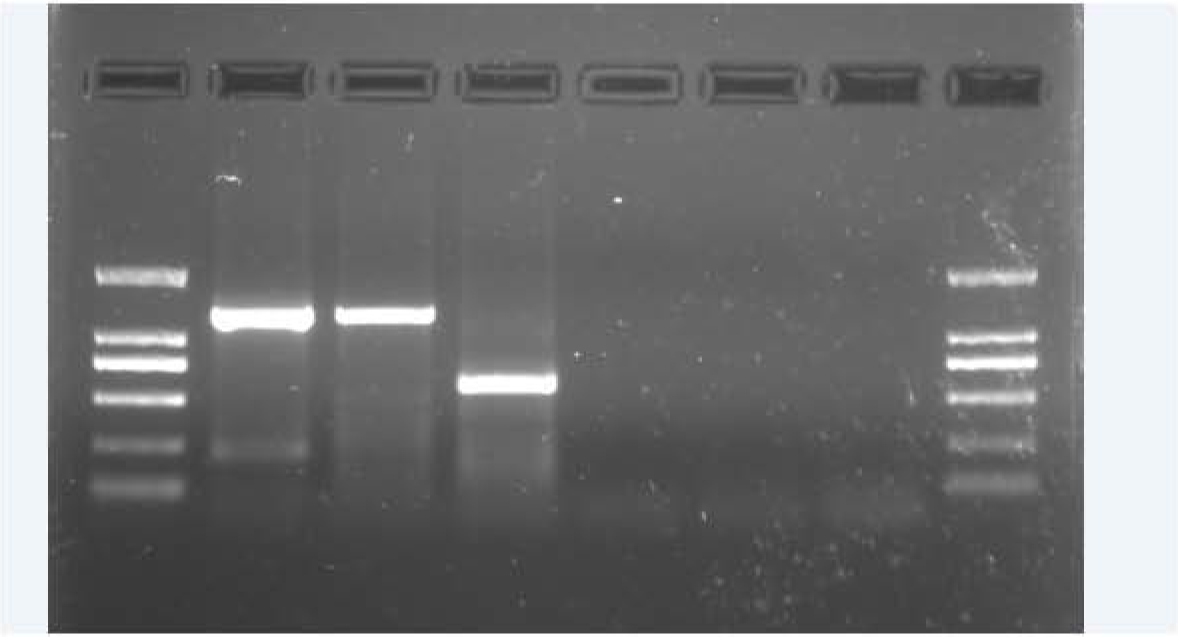

S.aureus standard strain

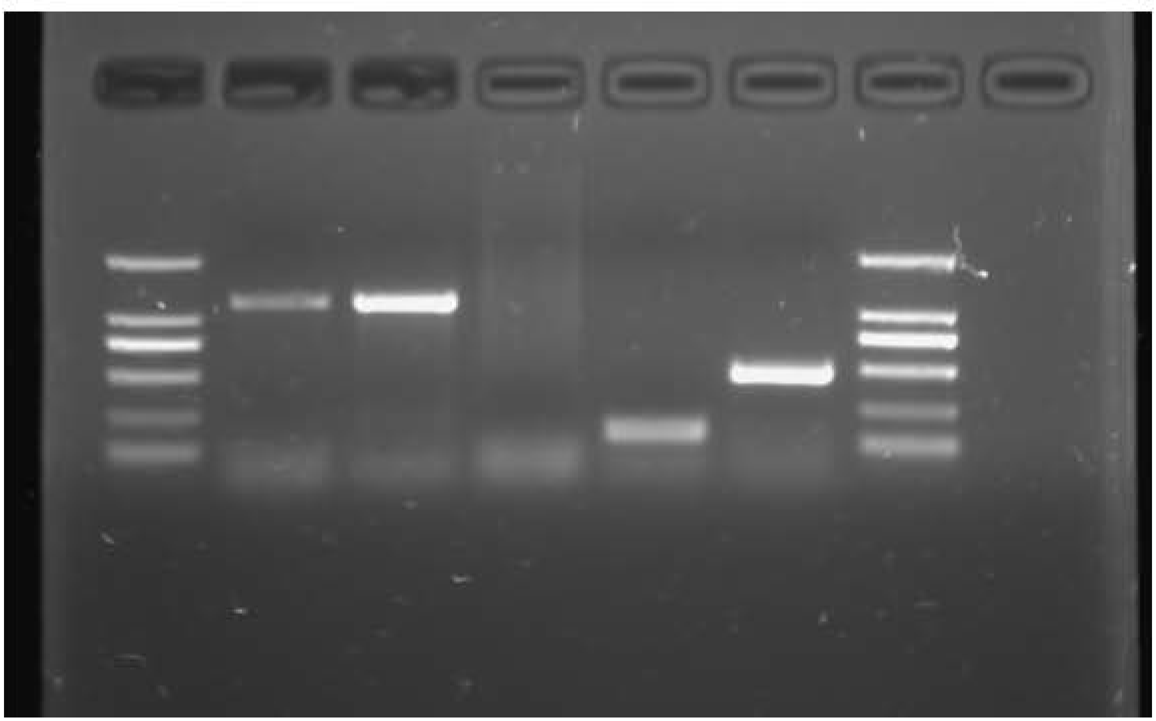

S.coli standard strain

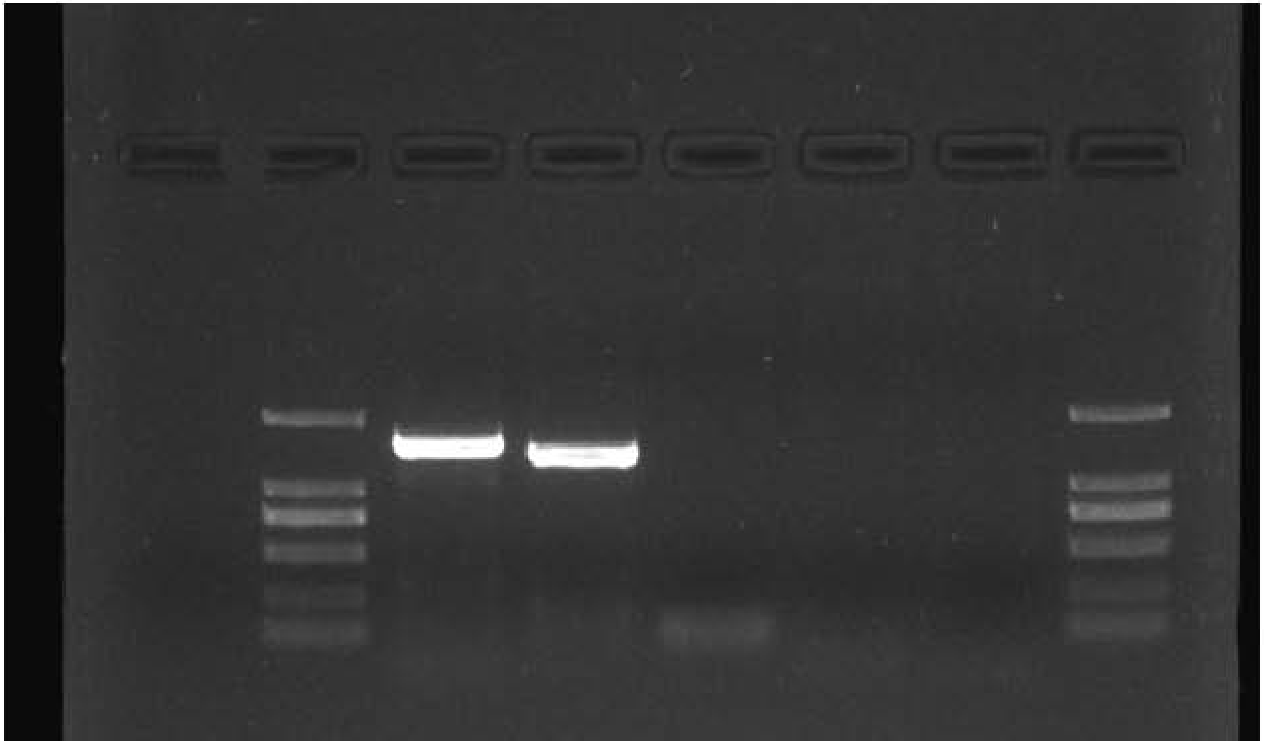

S.epidermidis standard strain

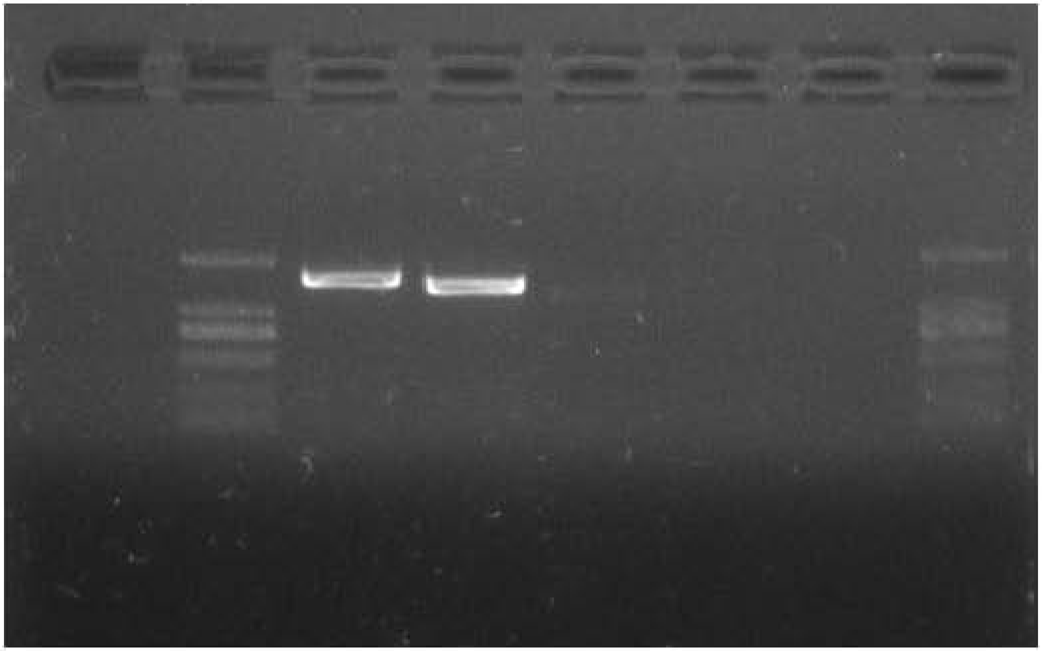

S.pneumoniae standard strain(left)and other specimen(right)

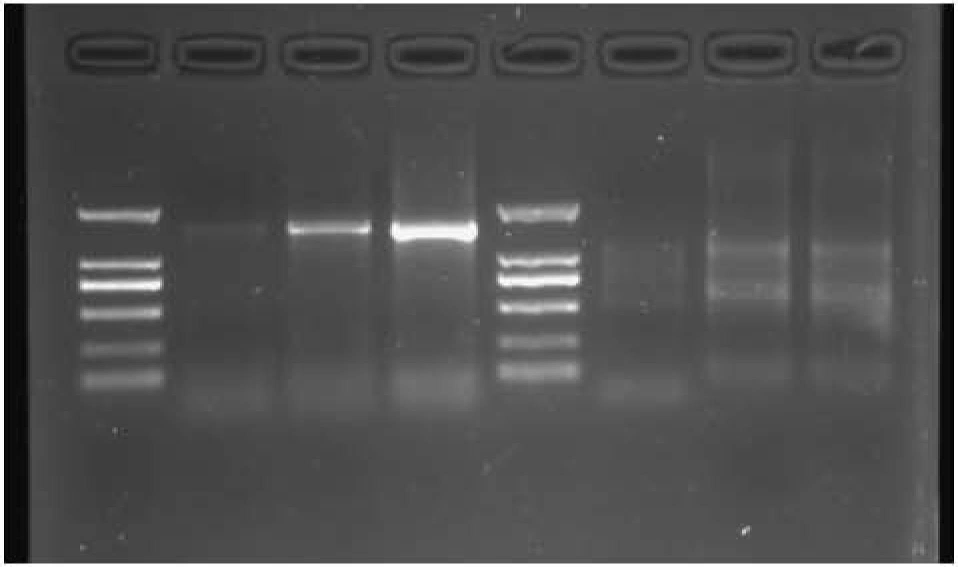

Specimen 5（gel image）

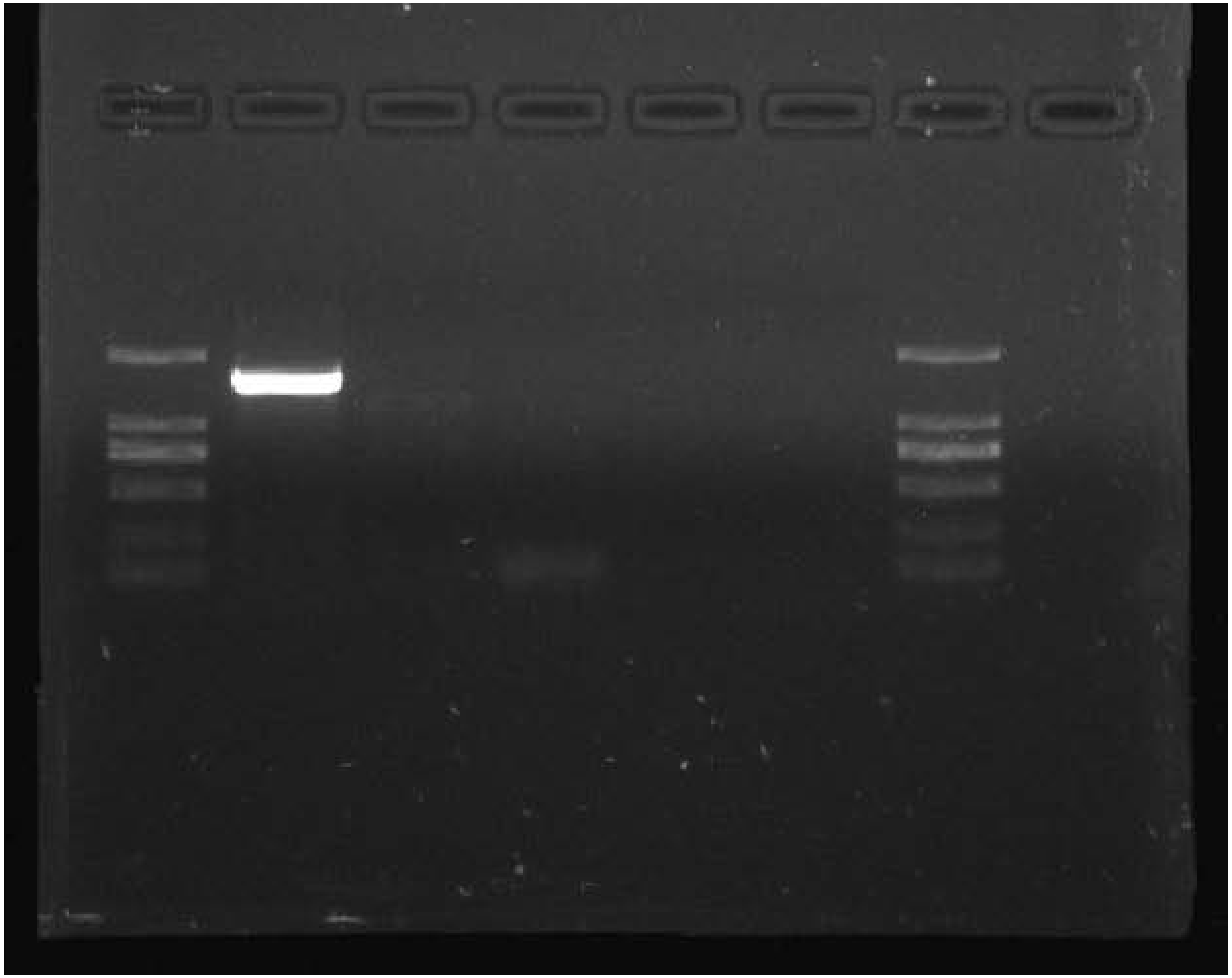

Specimen 13（gel image）

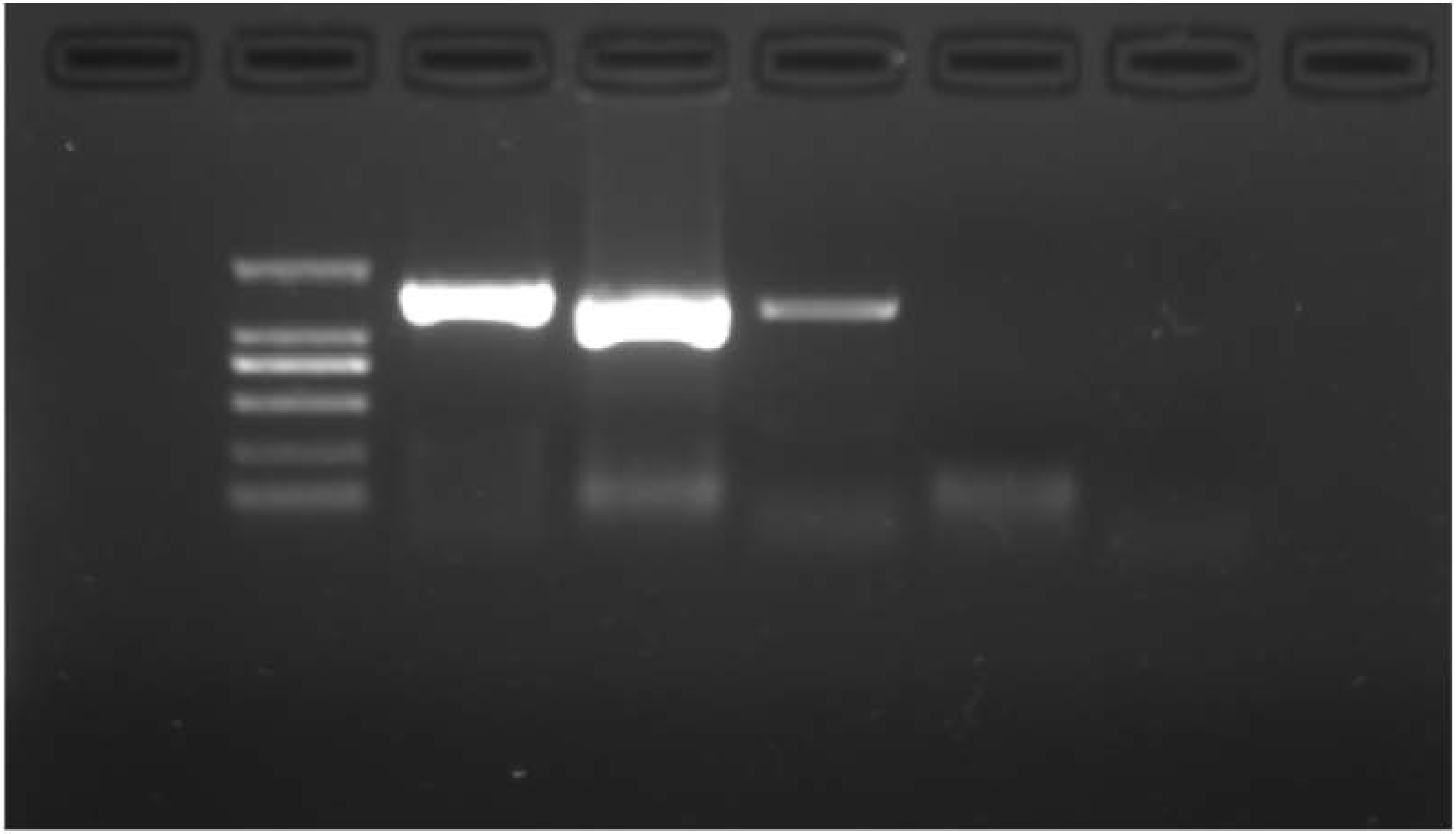

NC Specimen 1

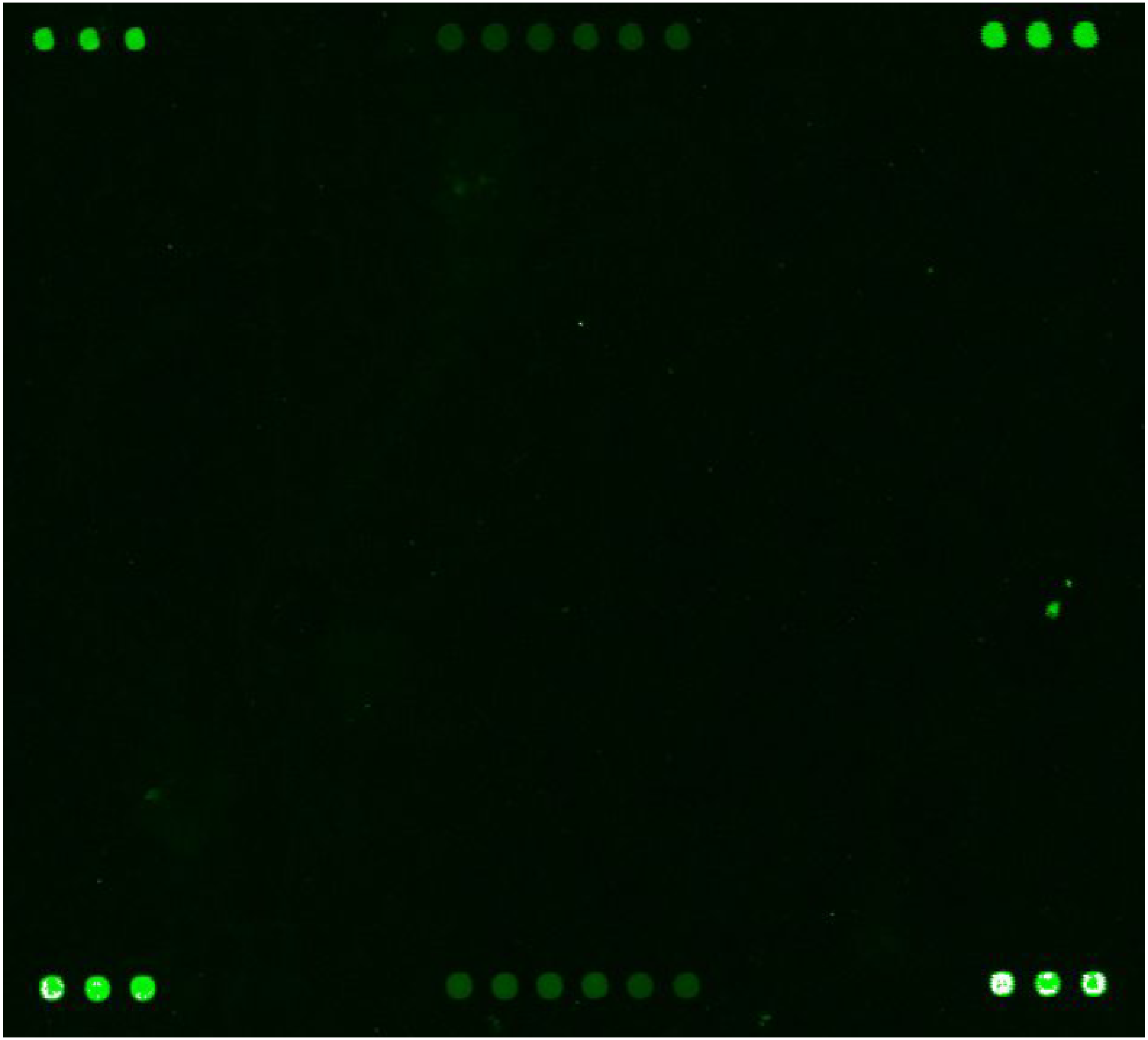

NC Specimen 2

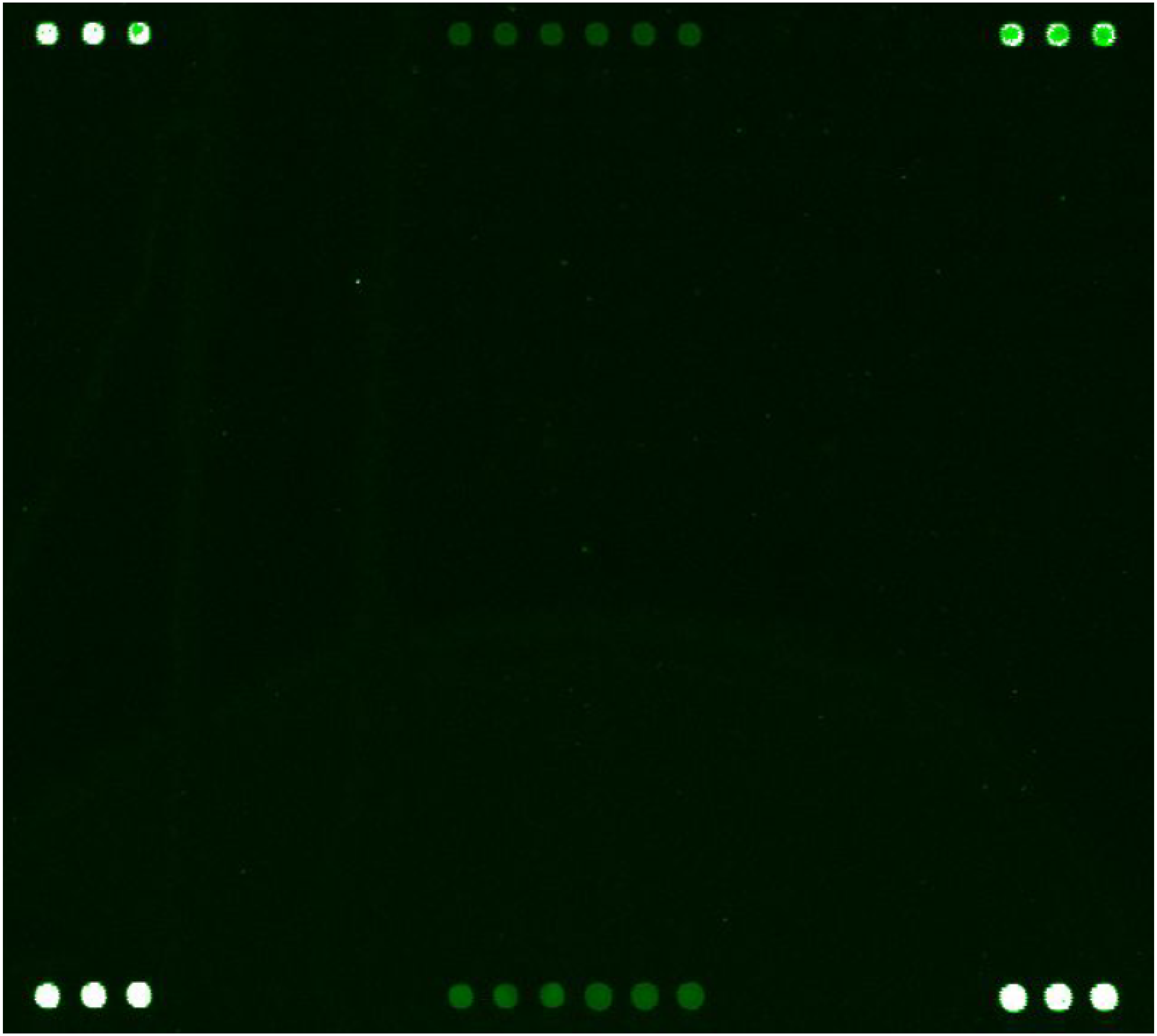

NC Specimen 3

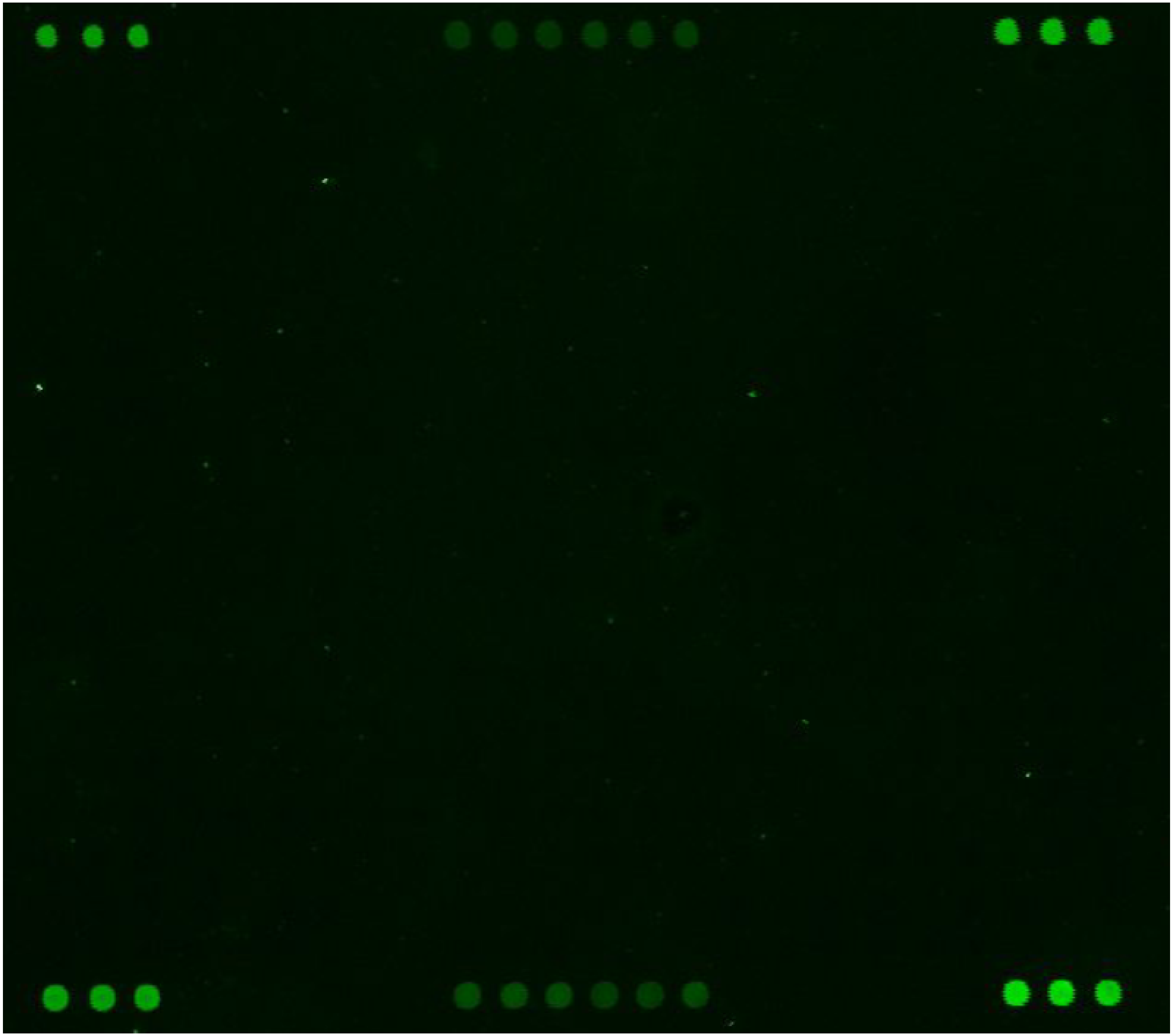

NC Specimen 4

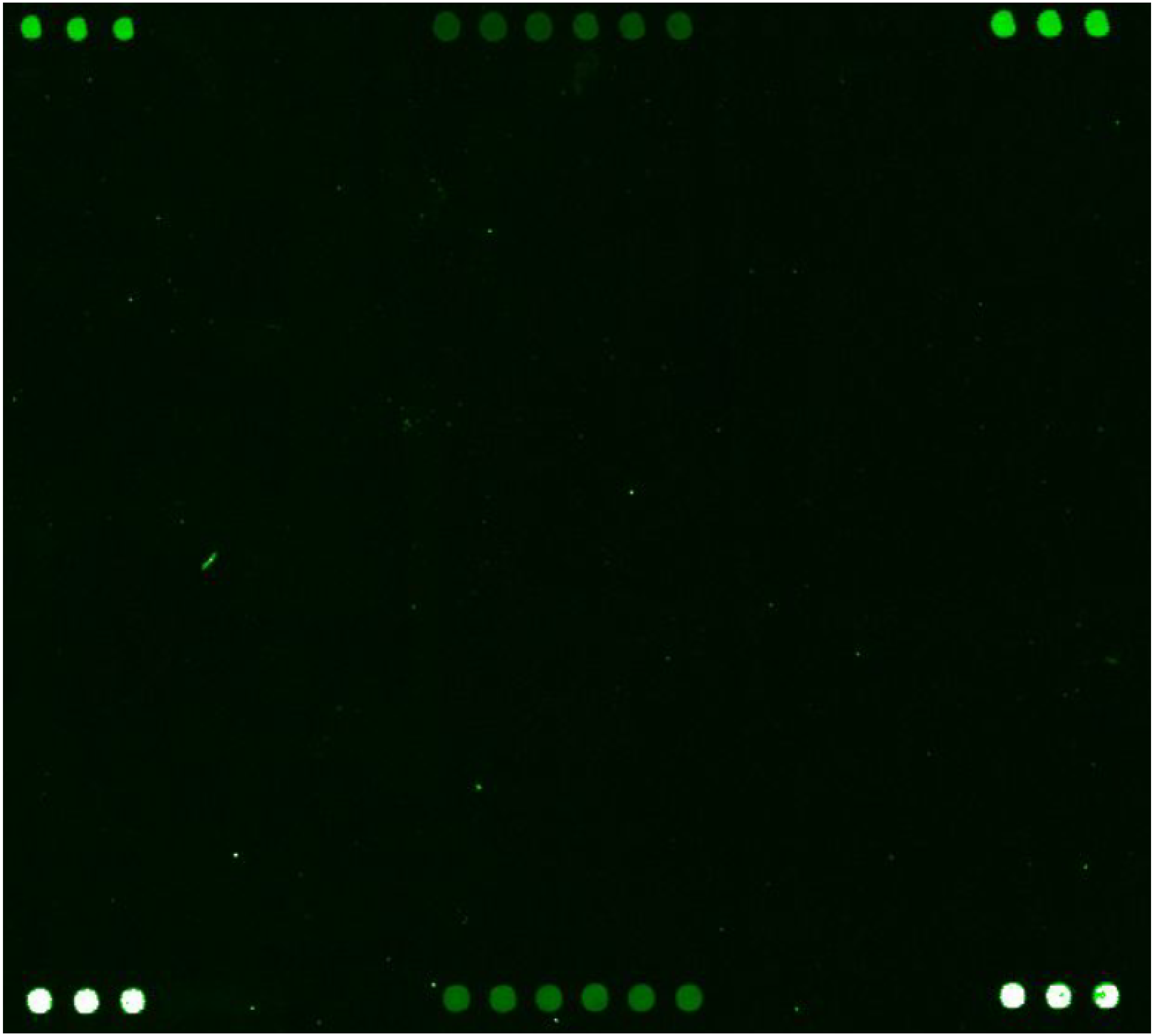

NC Specimen 5

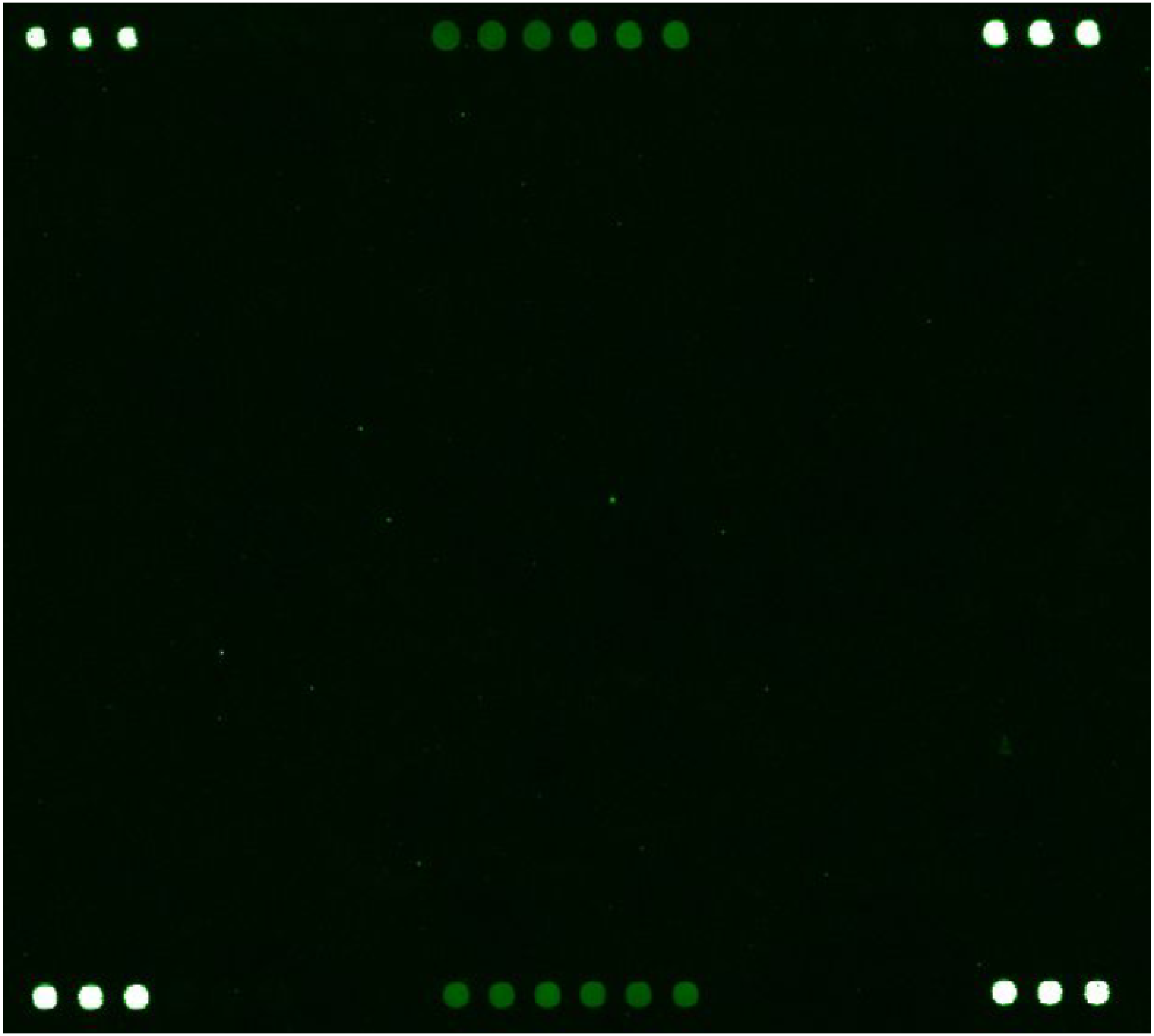

NC Specimen 6

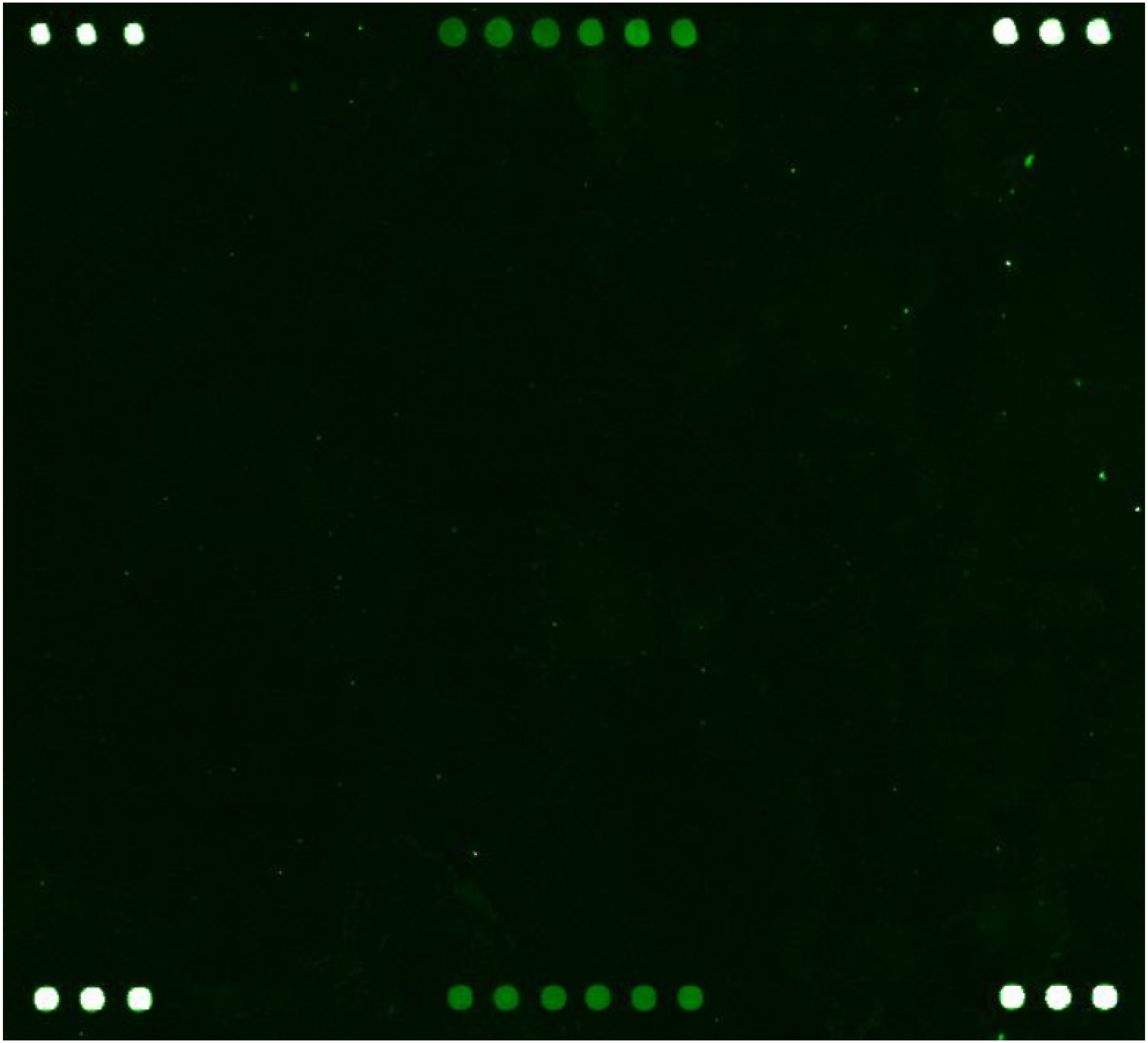

NC Specimen 7

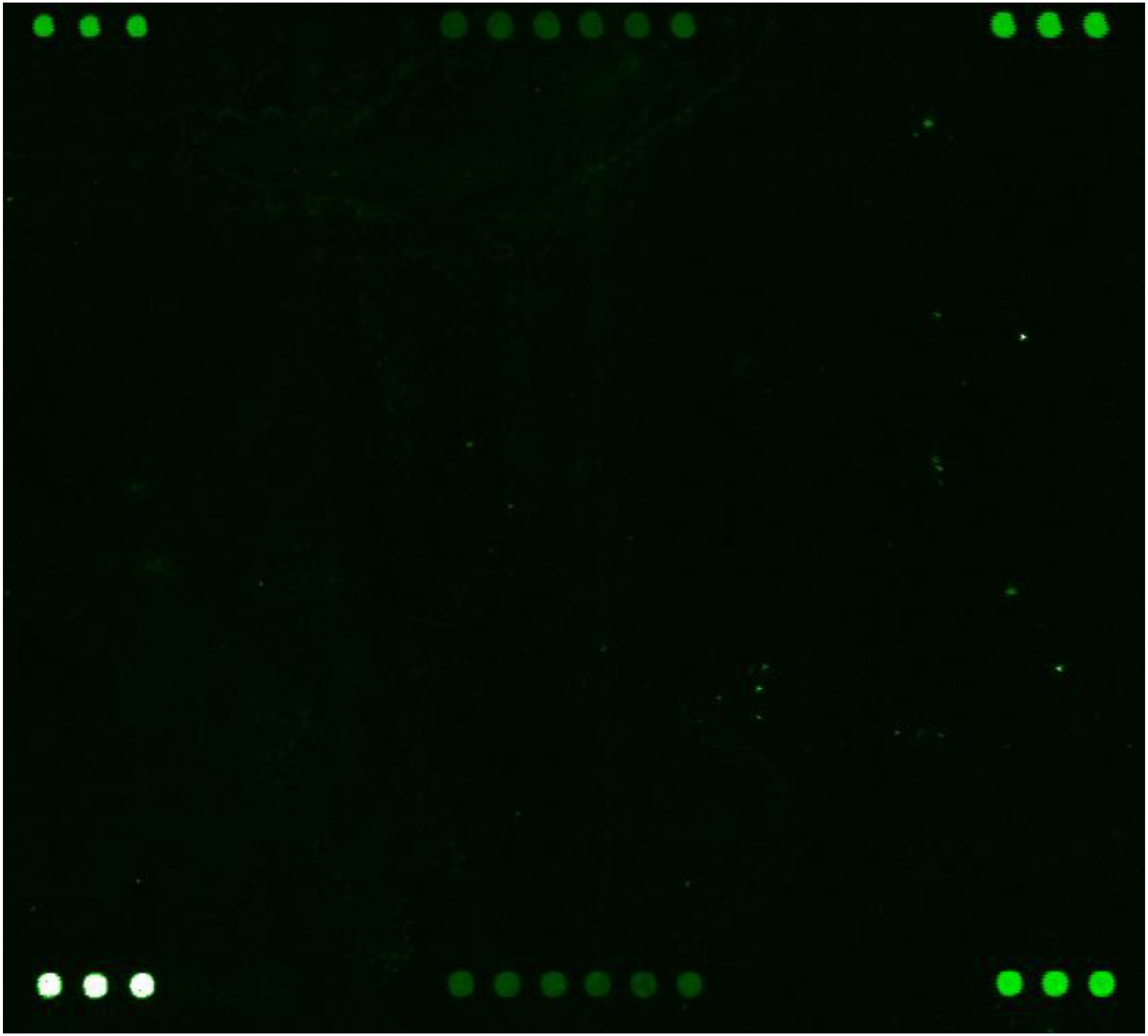

NC Specimen 8

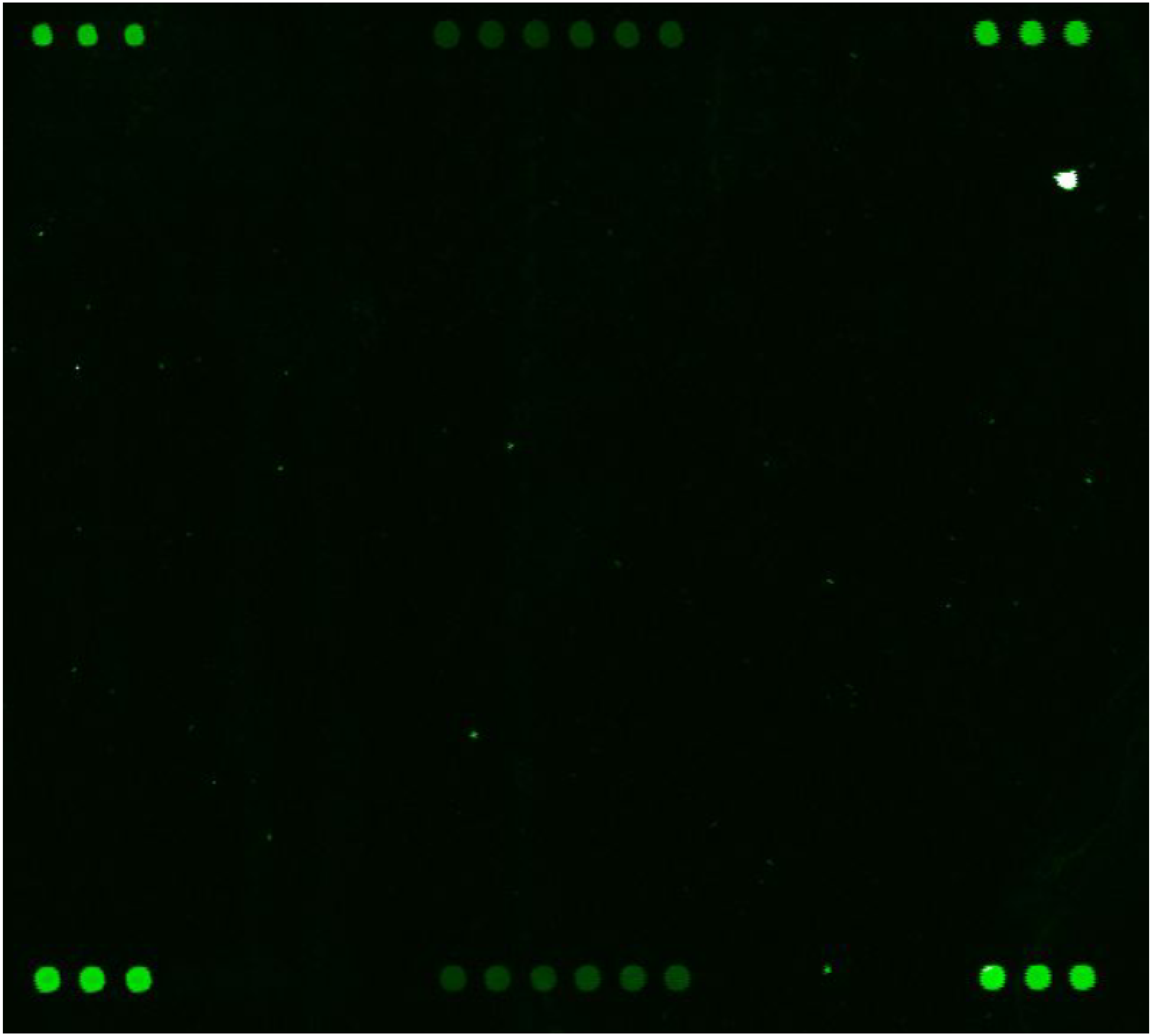

NC Specimen 9

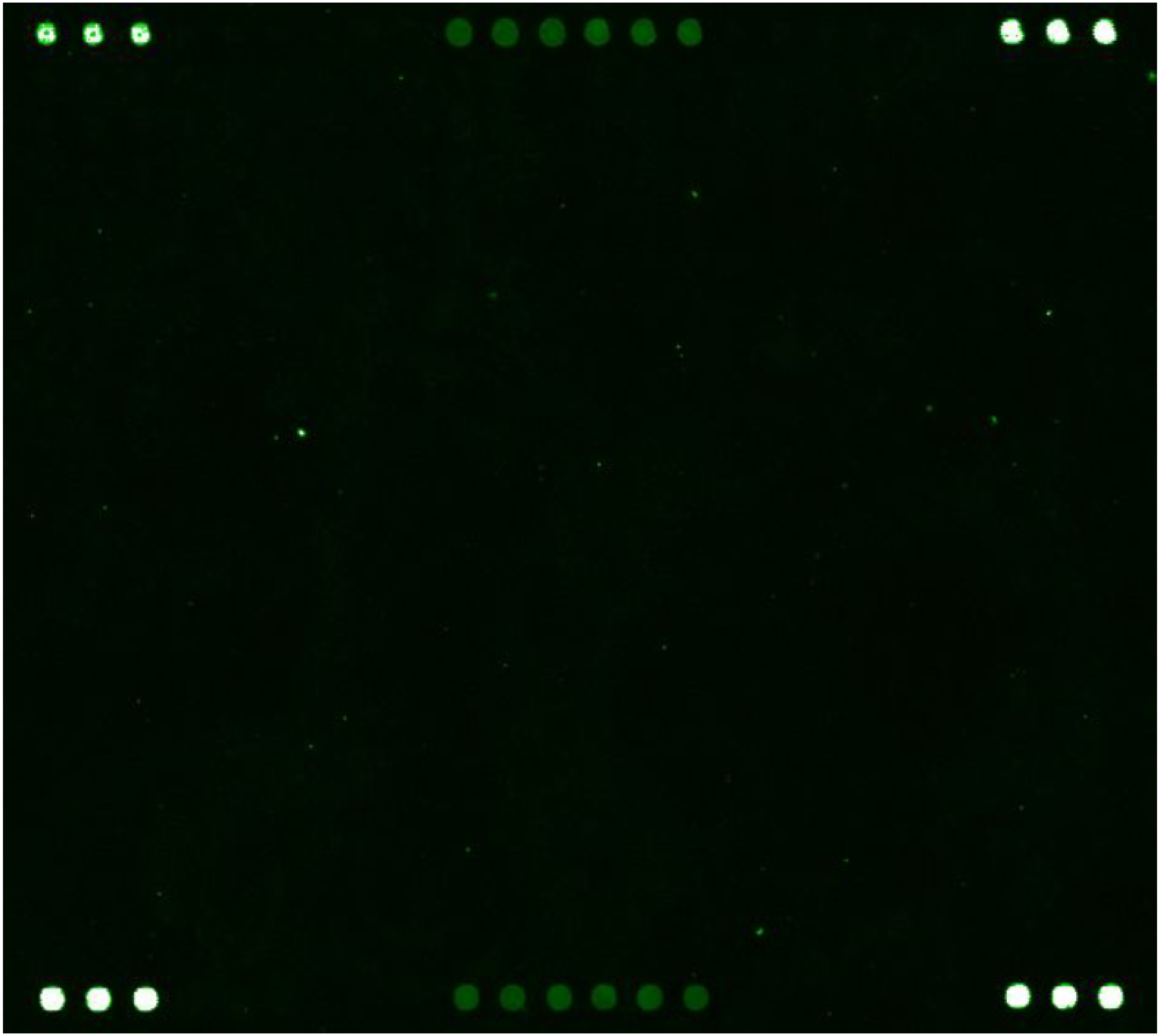

NC Specimen 10

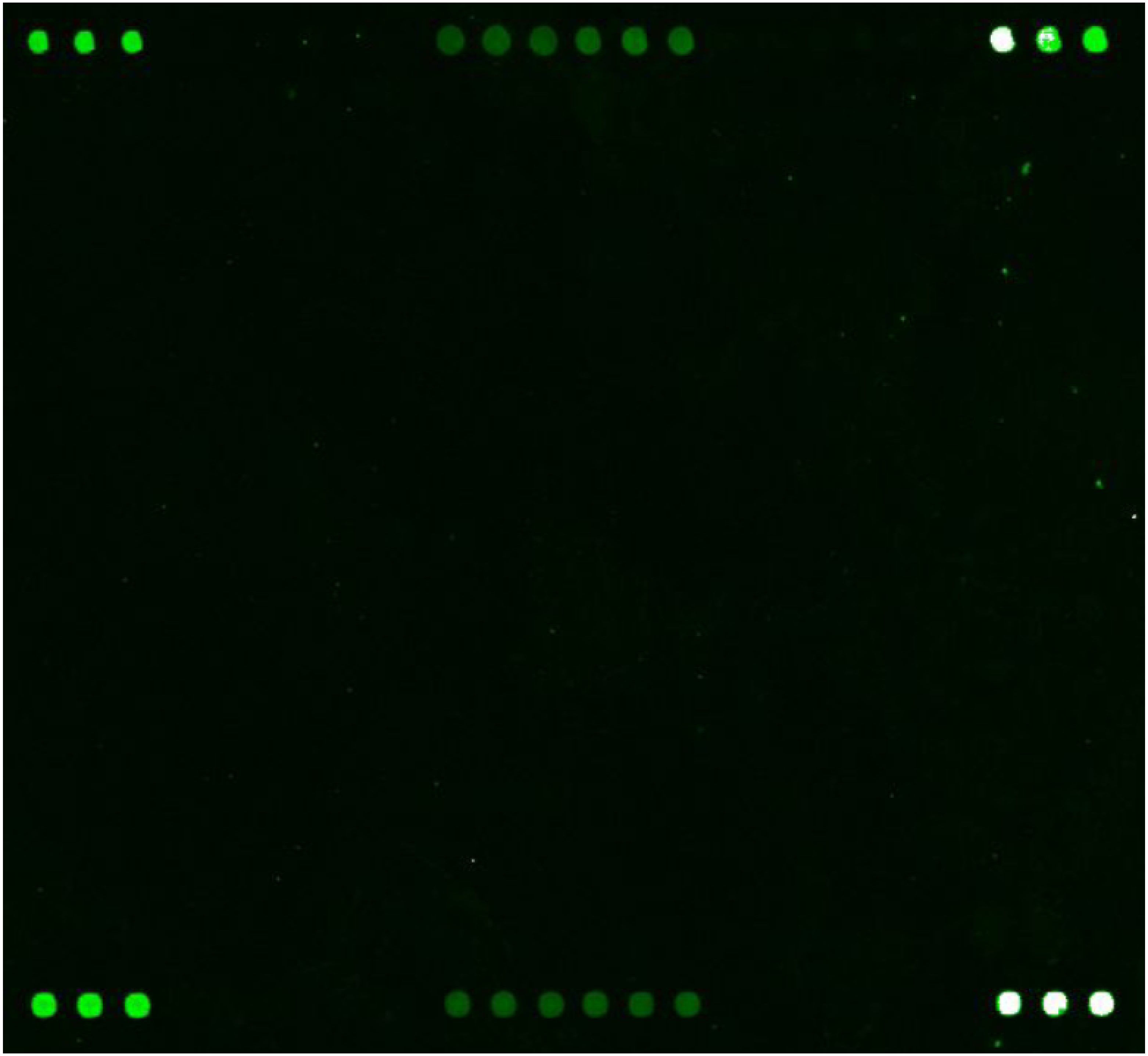

NC Specimen 11

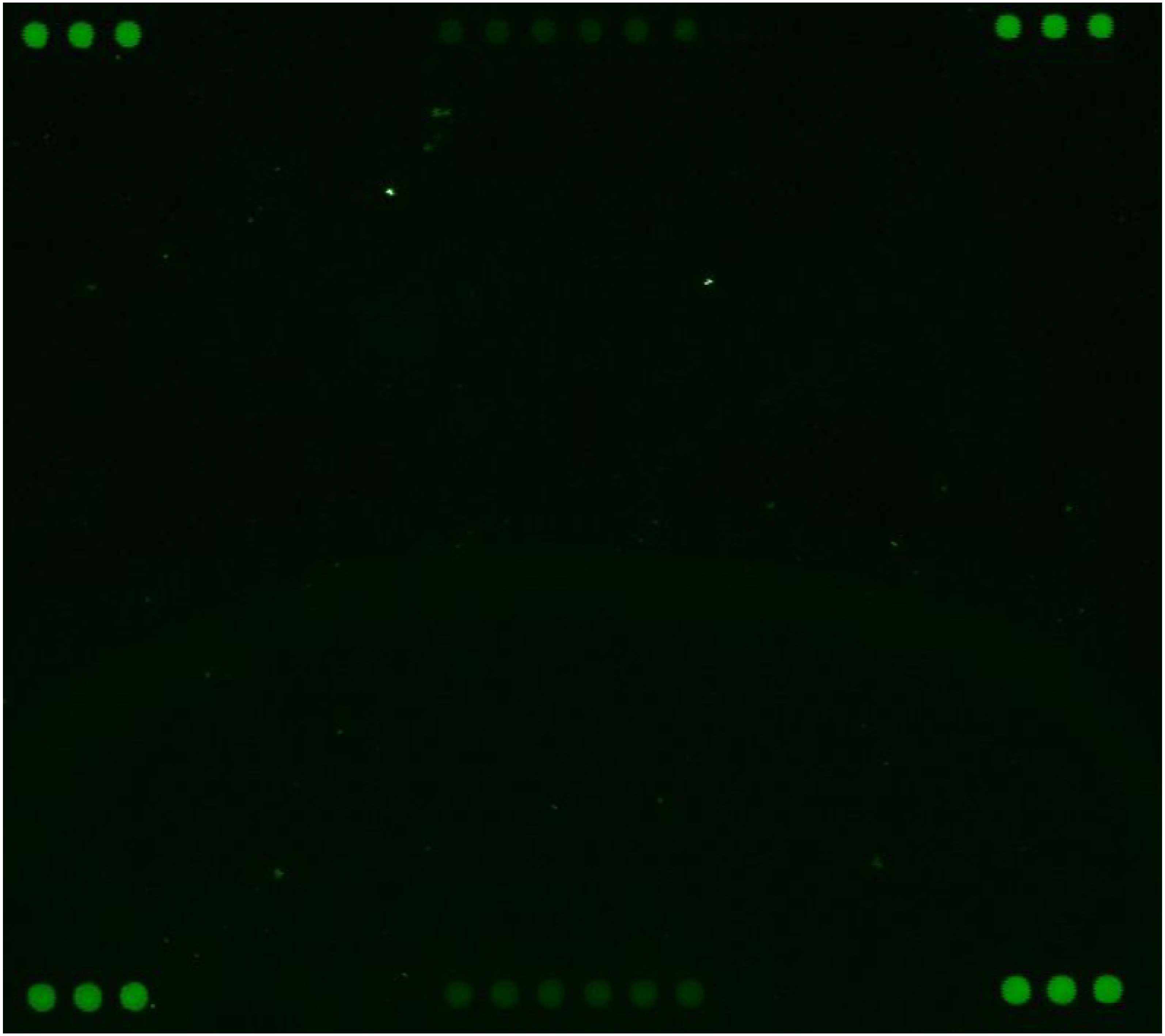

NC Specimen 12

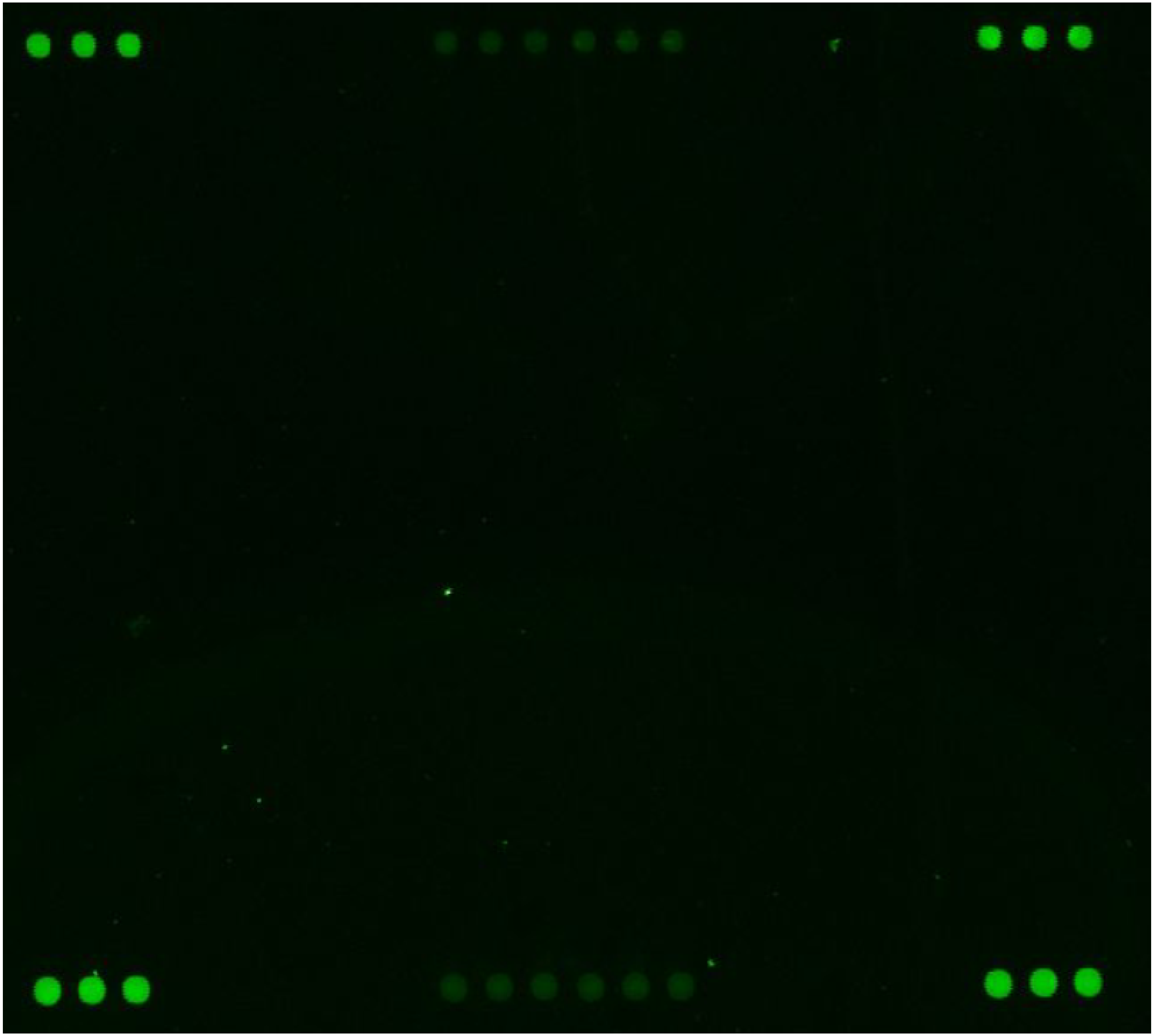

NC Specimen 13

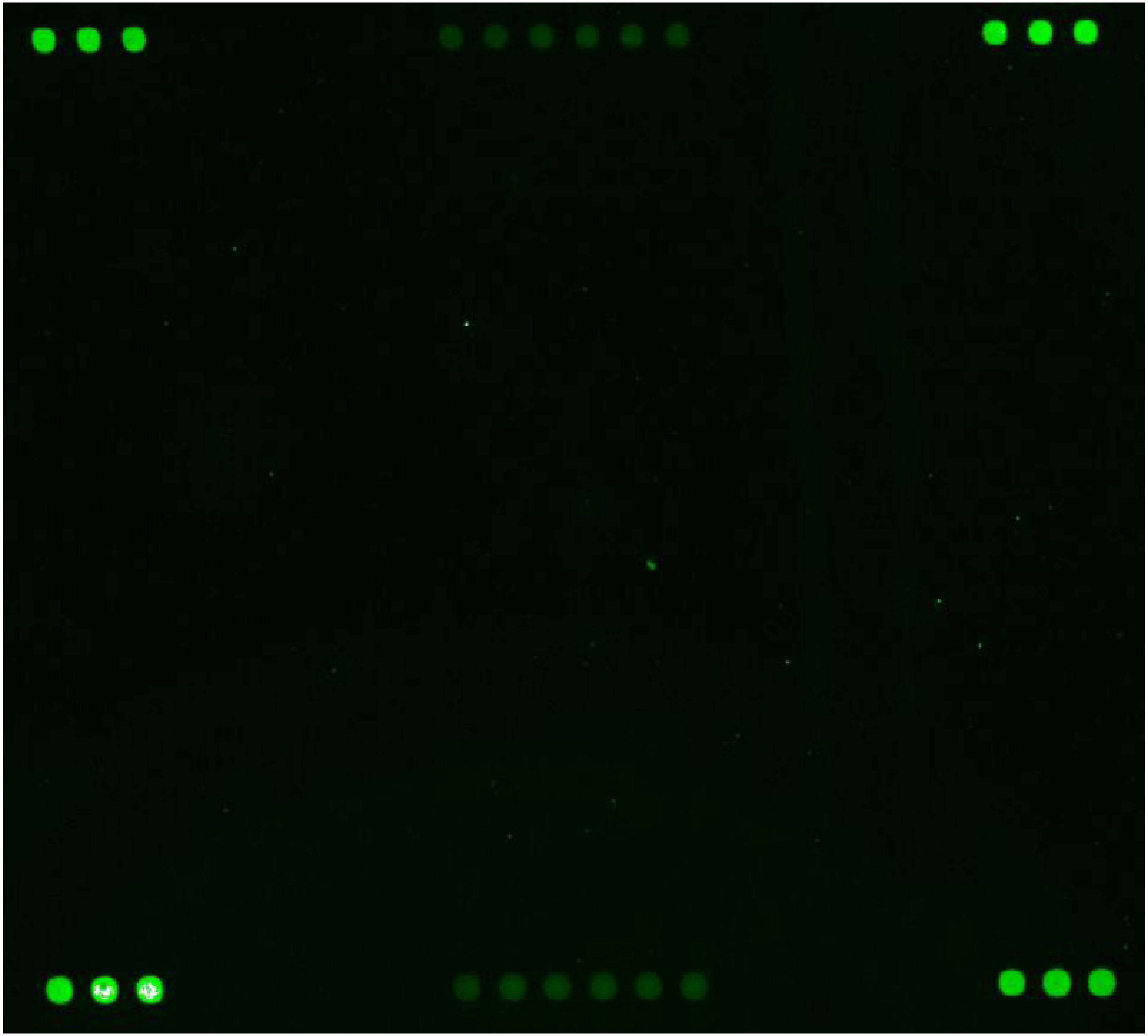

NC Specimen 14

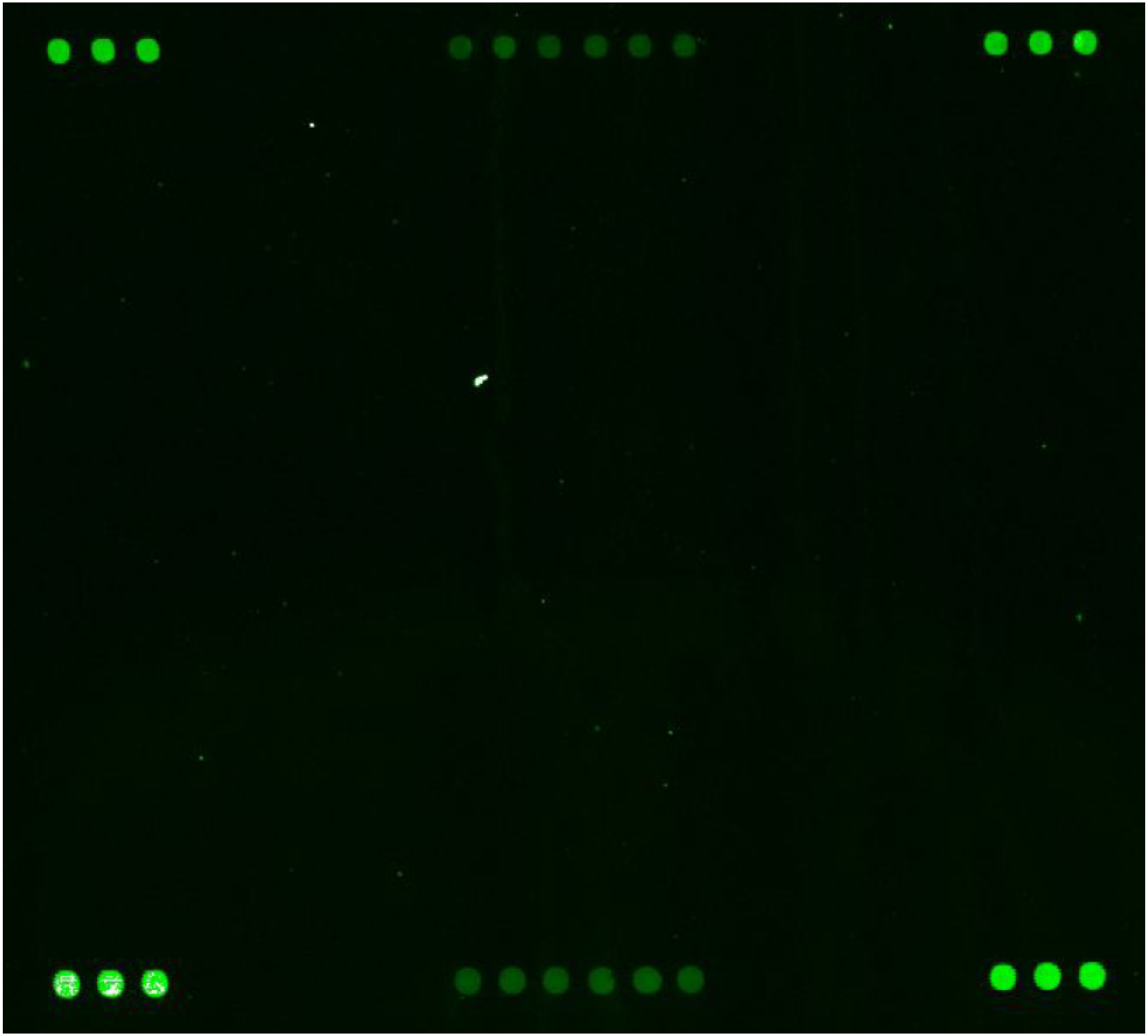

NC Specimen 15

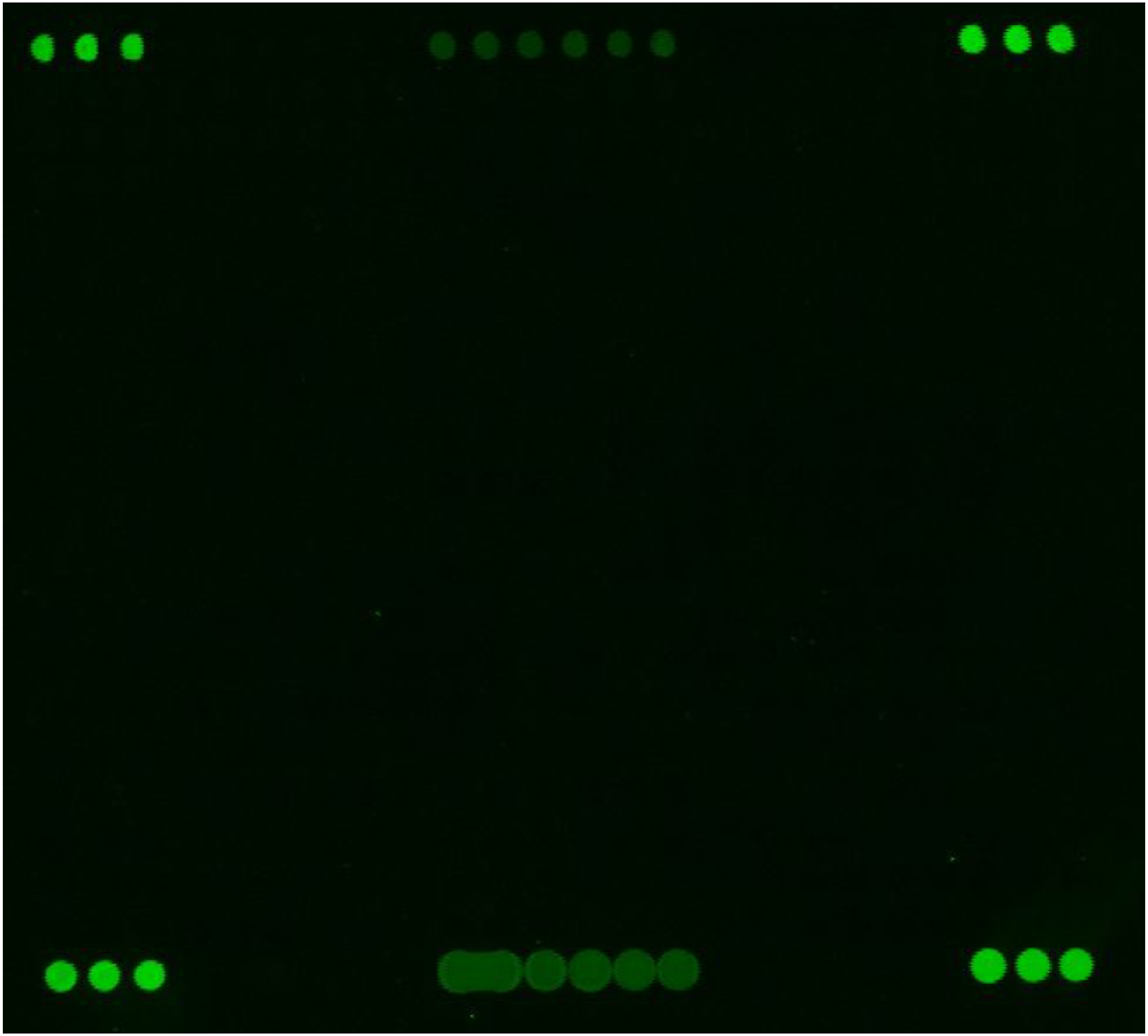

NC Specimen 16

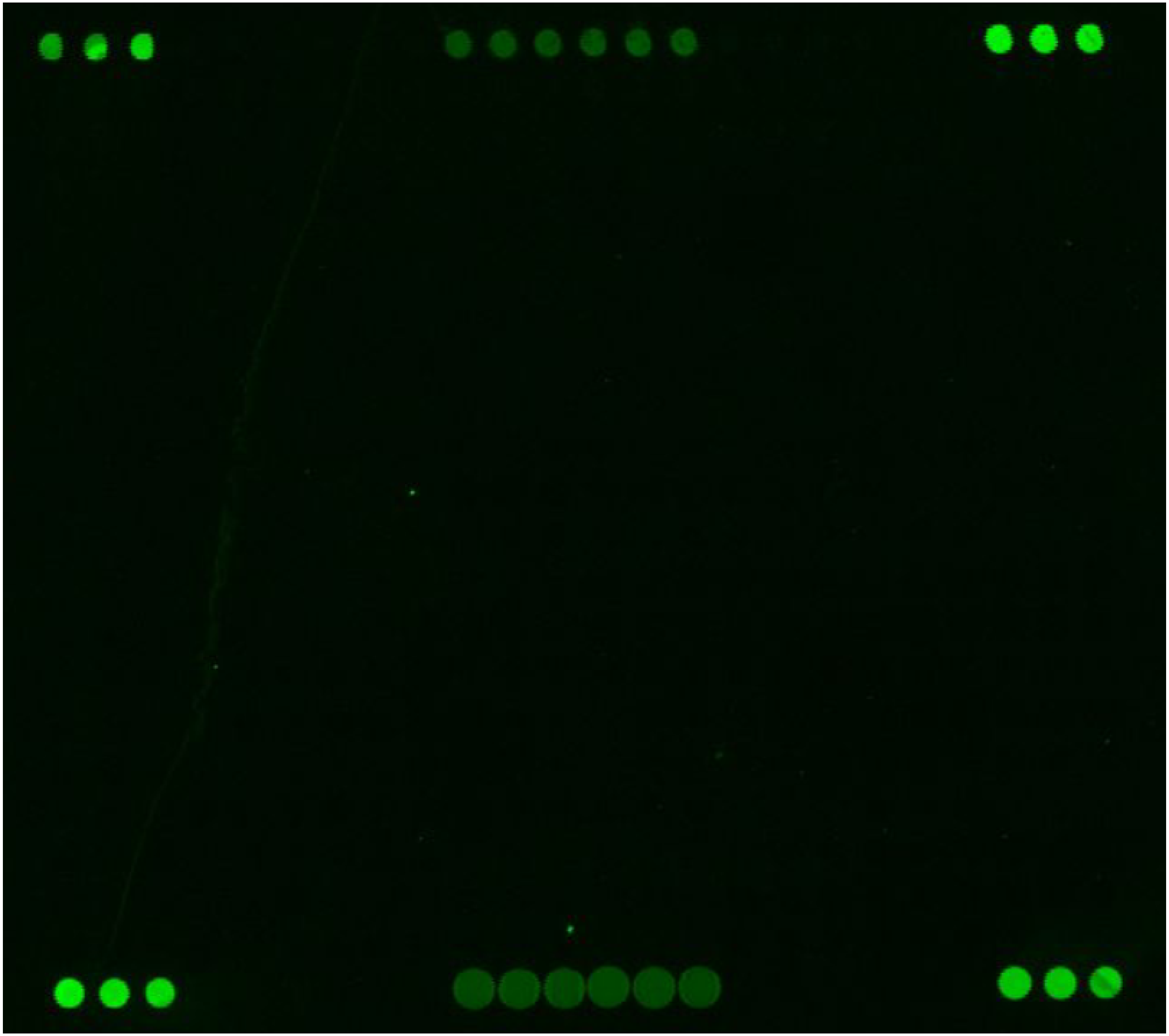

NC Specimen 17

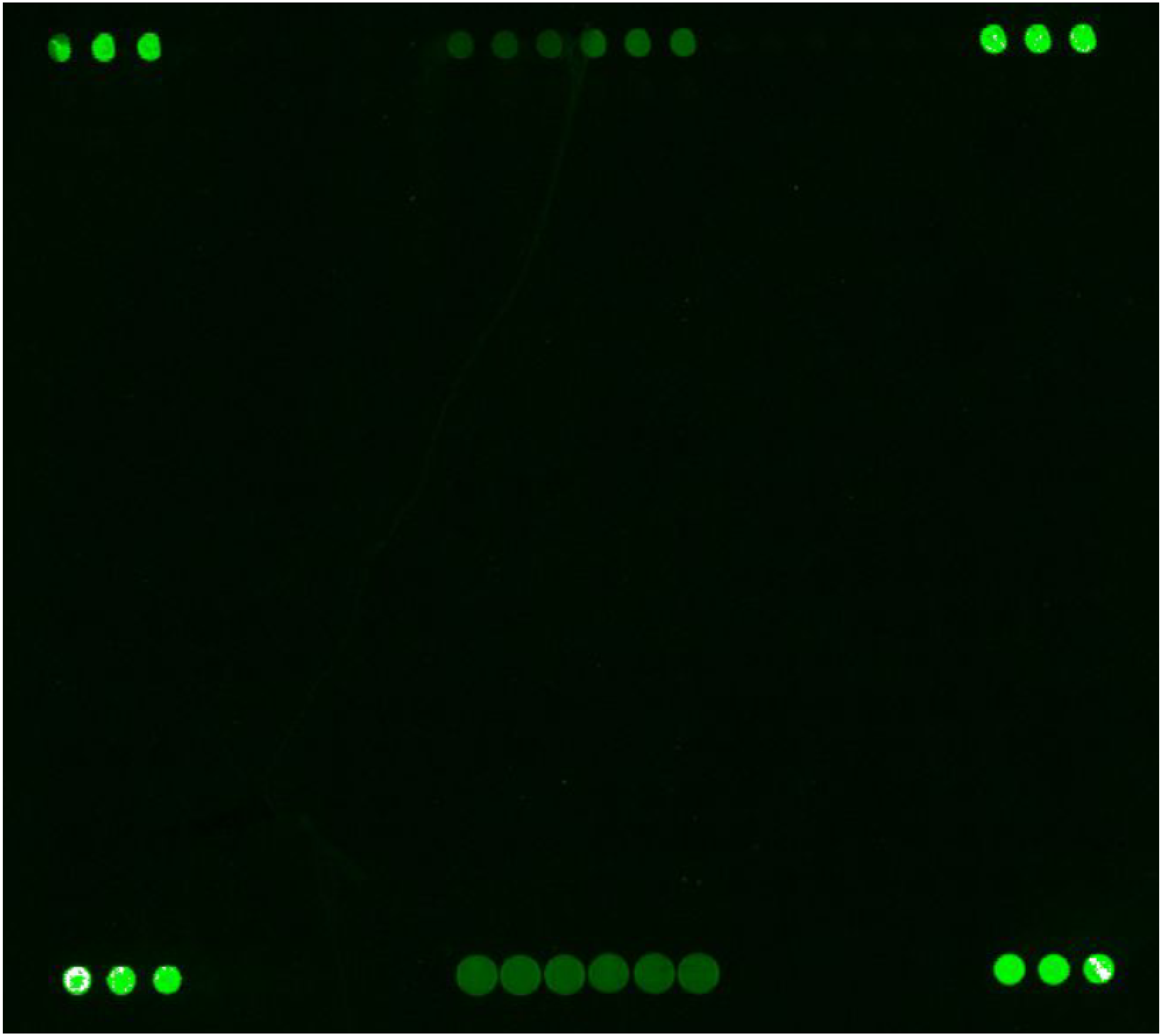

NC Specimen 18

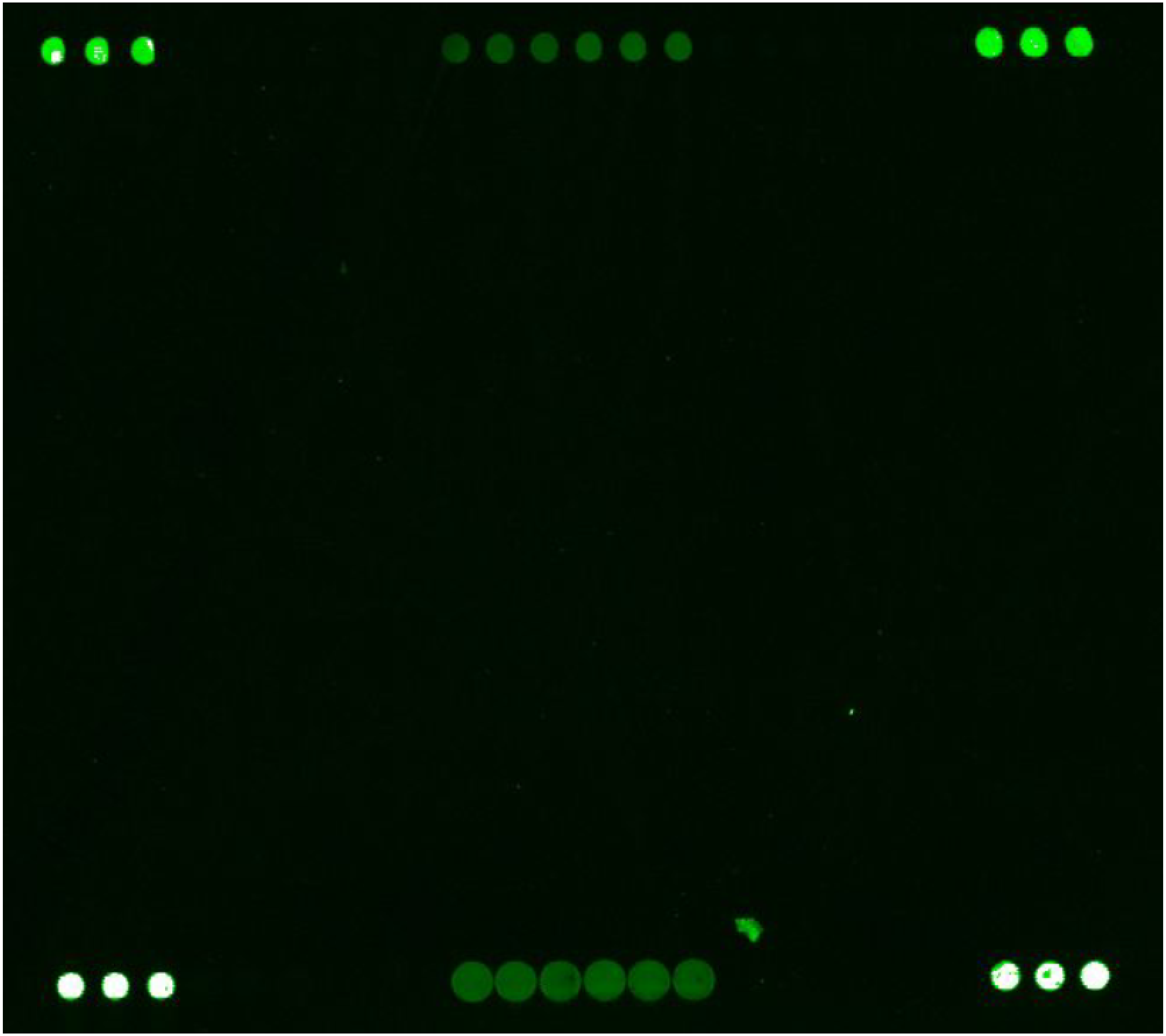

NC Specimen 19

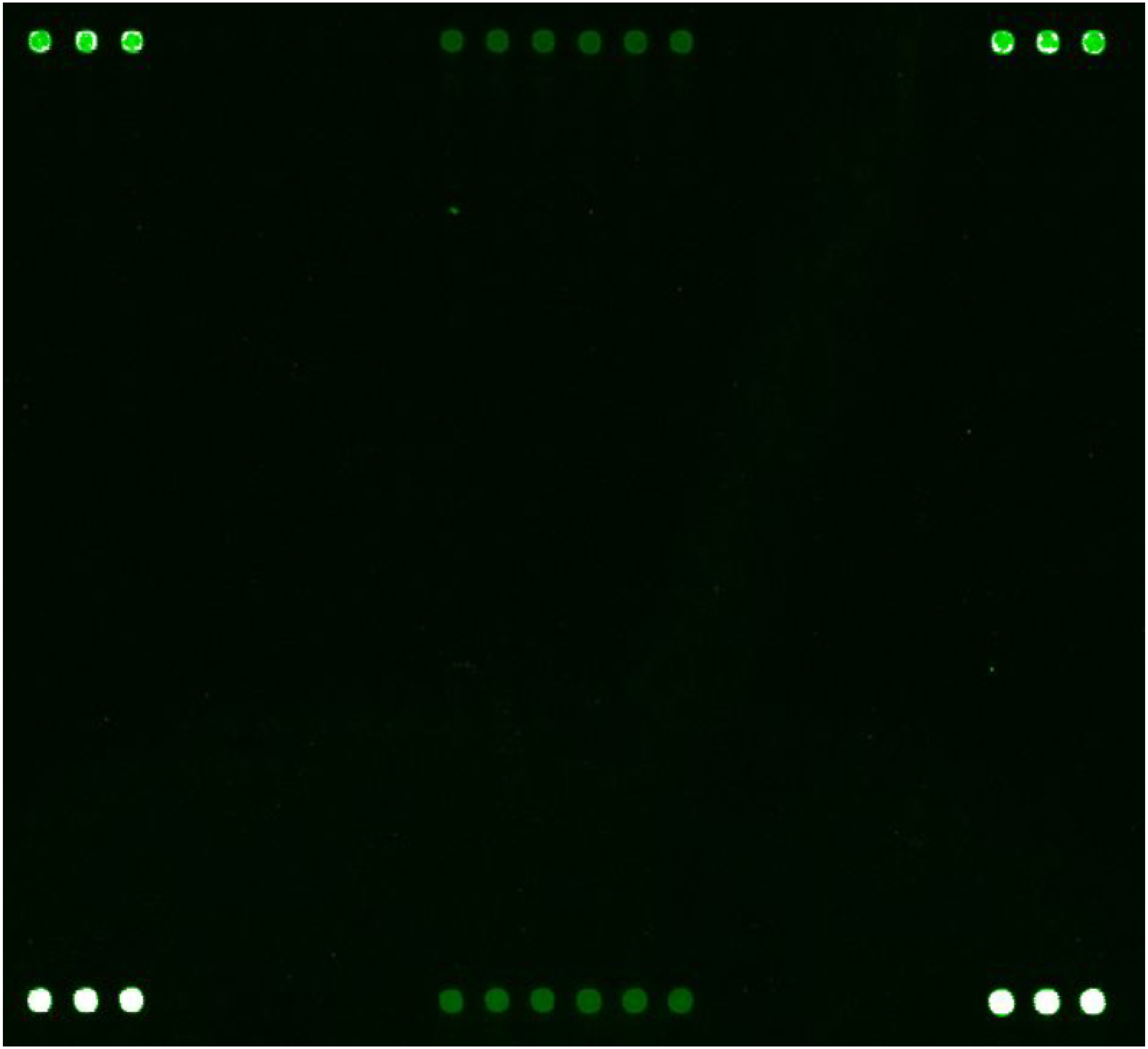

NC Specimen 20

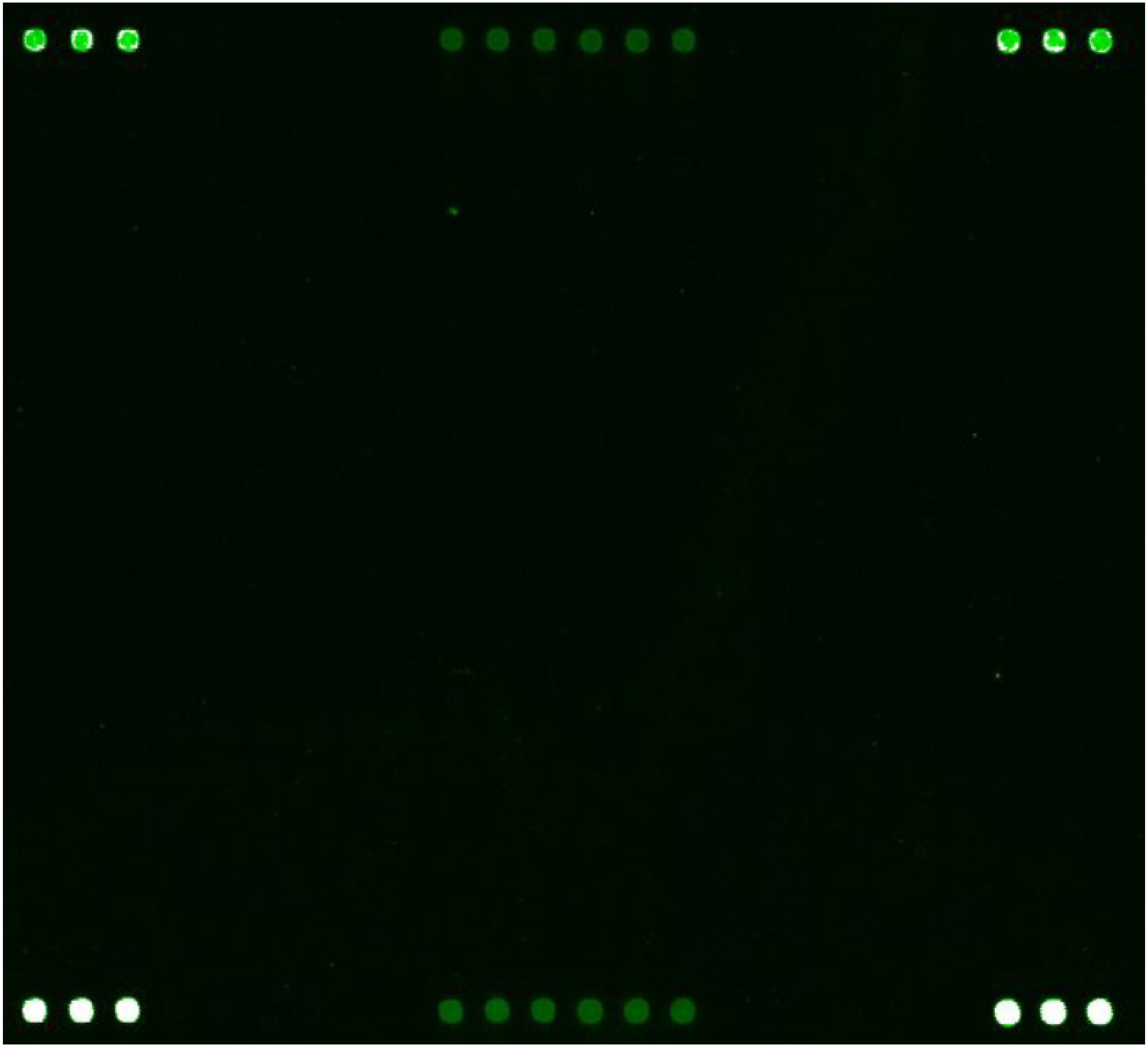

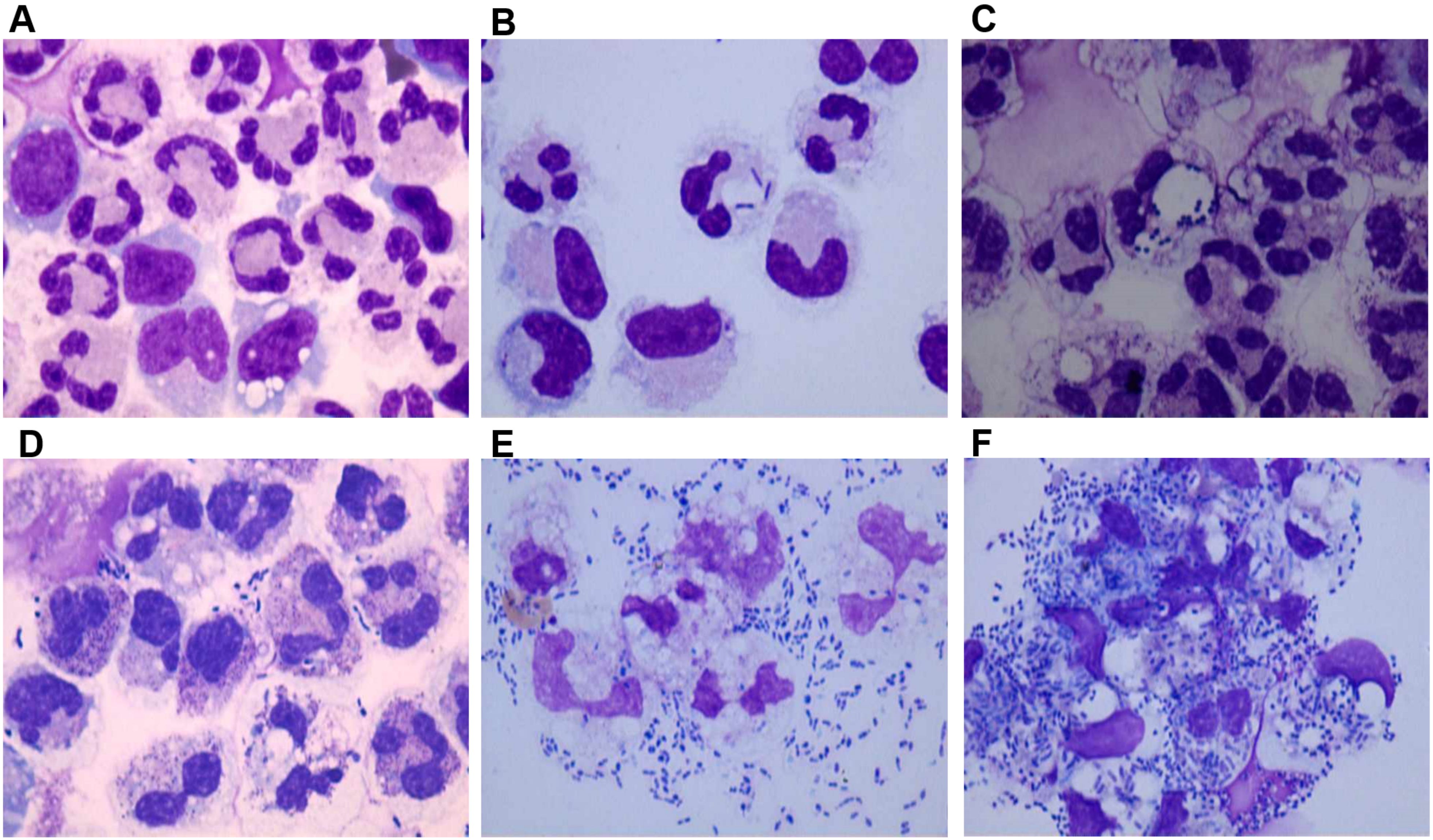

Figure S1. Cytological examination of CSF（1000×）A. Negative of infection; B. Neutrophils with intracellular bacterial infection(Bacillus) ;C. Neutrophils with intracellular bacterial infection(Coccus); D. Extracellular bacterial infection(Bacillus) ; E. Extracellular bacterial infection(Coccus); F. Extracellular and intracellular bacterial infection

Figure S2 Negative control specimens` microarry analysis results

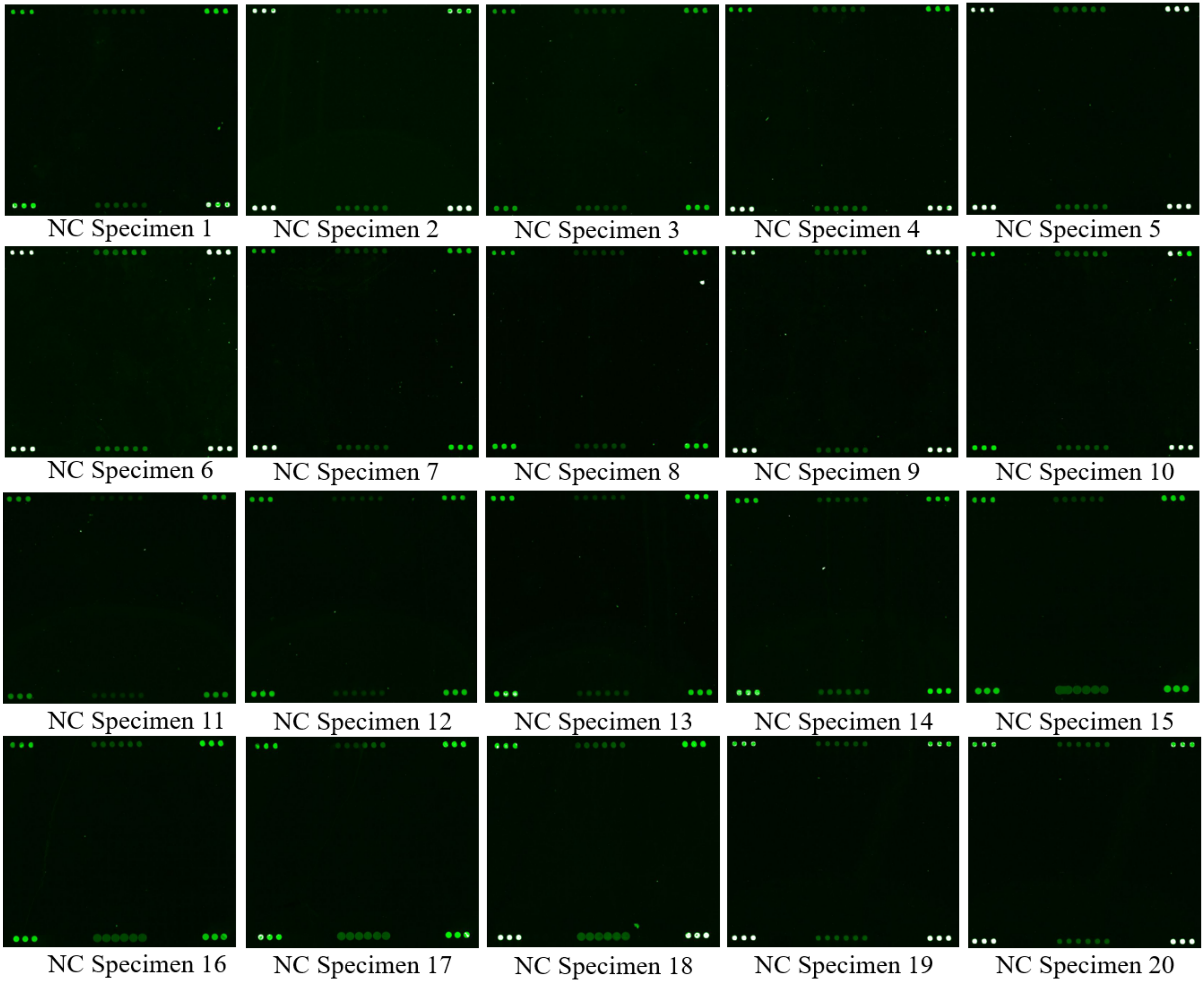

Table S1. The results of negative control specimens` CSF culture testing, related clinical testing

Sample ID	Age/sex (years)	Symptom	The chemical analysis of CSF			CSF cytology examination					The results of CSF culture testing	The results of CSF microarry analysis	Diagnosis
			Pro (g/L)	Glu (mmol/L)	Cl (mmol/L)	WBC (/mm^3)	L (%)	M (%)	N (%)	Bacteria			
NC Specimen 1	45/F	Bladder difficulties, Limbs numbness	0.63	3.0	123	27	92	6	4	NO	Negative	Negative	Neuromyelitis optica
NC Specimen 2	32/M	Limbs numbness	0.39	2.7	128	2	-	-	-	NO	Negative	Negative	Neuromyelitis optica
NC Specimen 3	16/F	Headache, Tic	0.41	3.2	125	37	91	6	3	NO	Negative	Negative	Anti-NMDA receptor encephalitis
NC Specimen 4	23/M	Headache, Tic, Unconsciousness	0.41	2.9	121	22	90	4	6	NO	Negative	Negative	Anti-NMDA receptor encephalitis
NC Specimen 5	63/M	Fever Unconsciousness	0.66	3.4	117	315	38	21	41	NO	Negative	Negative	Epidemic encephalitis B
NC Specimen 6	49/M	Fever, Headache,	0.89	2.7	121	25	99	1	-	NO	Negative	Negative	Epidemic encephalitis B
NC Specimen 7	82/M	Aphasia	0.90	4.0	115	70	80	18	2	NO	Negative	Negative	Cerebral infarction
NC Specimen 8	52/F	Headache,	0.33	2.7	126	7	-	-	-	NO	Negative	Negative	Cerebral infarction
NC Specimen 9	48/M	Headache, Limbs weakness	0.77	3.1	125	10	89	11	-	NO	Negative	Negative	Cerebral infarction
NC Specimen 10	31/F	Weakness of the muscles of the face, Limbs numbness	4.53	2.6	119	10	65	33	2	NO	Negative	Negative	Guillain-barre syndrome
NC Specimen 11	67/M	Problems with speech, Unconsciousness	0.78	3.4	114	162	81	17	2	NO	Negative	Negative	Guillain-barre syndrome
NC Specimen 12	32/F	Limbs numbness	0.44	3.1	120	5	-	-	-	NO	Negative	Negative	Multiple sclerosis
NC Specimen 13	30/F	Blurred vision, Limbs numbness	0.43	4.9	124	12	97	3	-	NO	Negative	Negative	Multiple sclerosis

NC Specimen 14	24/F	Headache	0.88	1.7	121	67	96	4	-	NO	Negative	Negative	Viral meningitis
NC Specimen 15	34/F	Headache	1.01	2.3	125	222	97	3	-	NO	Negative	Negative	Viral meningitis
NC Specimen 16	34/F	Headache, Fever	0.56	1.9	124	127	88	12	-	NO	Negative	Negative	Viral meningitis
NC Specimen 17	29/M	Headache	0.98	2.5	122	177	92	8	-	NO	Negative	Negative	Viral meningitis
NC Specimen 18	8/M	Headache，Anemia	-	-	-	2	-	-	-	NO	Negative	Negative	Acute lymphoblastic leukemia
NC Specimen 19	62/M	Fever, Feeling tired	0.66	2.9	122	10	25	3	69	NO	Negative	Negative	Central nervous system leukemia
NC Specimen 20	19/M	Blurred vision	0.49	2.5	122	87	-	-	-	NO	Negative	Negative	Central nervous system leukemia

Notes: CSF-cerebrospinal fluid; Pro-protein contents of cerebrospinal fluid; Glu-glucose contents of cerebrospinal fluid; Cl-chloride contents of cerebrospinal fluid; WBC-white blood cells ofcerebrospinal fluid; L-lymphocyte count of cerebrospinal fluid; M-monocyte count of cerebrospinal fluid; N-neutrophils count of cerebrospinal fluid.
